# Click Chemistry Approach to Derivatisation of Fluconazole

**DOI:** 10.3390/molecules31142545

**Published:** 2026-07-22

**Authors:** Michał Janowski, Oleg M. Demchuk, Sylwia Andrzejczuk, Angelika Pawlicka, Urszula Kosikowska, Marta Struga, Marcin Feldo, Monika Wujec

**Affiliations:** 1 Doctoral School, Medical University of Lublin, Chodzki 7, 20-093 Lublin, Poland; 2 Faculty of Medicine, The John Paul II Catholic University of Lublin, Konstantynów 1J, 20-708 Lublin, Poland; 3 Department of Pharmaceutical Microbiology, Faculty of Pharmacy, Medical University of Lublin, 1 Chodzki St., 20-093 Lublin, Poland; sylwia.andrzejczuk@umlub.edu.pl (S.A.);; 4 Department of Biochemistry, Faculty of Medicine, Medical University of Warsaw, Banacha St. 1, 02-097 Warsaw, Poland; 5 Department of Vascular Surgery, Medical University of Lublin, Staszica 11 St., 20-081 Lublin, Poland; 6 Department of Organic Chemistry, Faculty of Pharmacy, Medical University of Lublin, 4a Chodzki St., 20-093 Lublin, Poland; monika.wujec@umlub.edu.pl

**Keywords:** fluconazole, 1,2,3-triazoles, 1,2,4-triazoles, CuAAC, click chemistry, azide-alkyne Huisgen cycloaddition, copper catalysis, antifungal agents, drug modification

## Abstract

The emergence of antimicrobial resistance necessitates the development of structurally novel agents with improved biological profiles. In this study, a series of new derivatives of fluconazole were designed, synthesized, and evaluated for antifungal activity. The synthetic approach involved esterification of hydroxyl group of fluconazole with an azide-group-containing benzoyl chloride, followed by copper-catalyzed azide–alkyne cycloaddition (CuAAC). The optimized catalytic system was based on a copper(I) catalyst generated from copper(II) palmitate and ascorbic acid. The transformations of the azide-containing derivatives were then performed. The obtained compounds were tested against reference strains of clinically relevant *Candida* spp., including *C. albicans* ATCC 10231, *C. parapsilosis* ATCC 22019, *C. krusei* ATCC 14243, *C. auris* CDC B11903, *C. tropicalis* ATCC 1369, *C. glabrata* ATCC 15126, and *C. lusitaniae* ATCC 3449. Some of the obtained derivatives showed very low toxicity in the MTT-based cell viability assay while maintaining high antifungal activity against selected strains.

## 1. Introduction

The increasing prevalence of antimicrobial resistance poses a significant threat to the effective treatment of infectious diseases worldwide [1]. In recent years, antifungal resistance has emerged as a particularly serious concern, especially in the case of opportunistic fungi such as *Candida* spp. and *Cryptococcus* spp. [2,3]. The reduced susceptibility or resistance of these microorganisms to azole antifungals is associated with multiple mechanisms, including mutations in target enzymes, overexpression of efflux pumps, and alterations in membrane sterol composition [4,5]. These limitations underscore the urgent need for novel antifungal agents [6,7] or chemically modified derivatives of established drugs [8,9].

Fluconazole is one of the most extensively used triazole antifungal agents and remains a cornerstone in the treatment of systemic and superficial fungal infections. Structurally, fluconazole is characterized by a symmetrical bis-heteroaromatic framework containing two 1,2,4-triazole rings linked to a difluorophenyl moiety via a hydroxylated carbon center. Its antifungal activity arises from selective inhibition of lanosterol 14α-demethylase (CYP51), a cytochrome P450 enzyme essential for ergosterol biosynthesis in fungal cell membranes [10]. Disruption of this pathway leads to impaired membrane integrity and inhibition of fungal growth [11].

Fluconazole was developed in the late 1980s as part of efforts to improve the pharmacological limitations of earlier imidazole-based antifungals [12]. The incorporation of triazole rings resulted in improved selectivity toward fungal enzymes and enhanced pharmacokinetic properties, including high oral bioavailability and low toxicity [13]. Despite these advantages, the extensive clinical use of fluconazole has contributed to the emergence of resistant strains, significantly reducing its long-term therapeutic efficacy [4,5,14]. As a result, fluconazole continues to serve as an attractive lead compound for structural modification aimed at restoring or enhancing antifungal activity.

Among modern synthetic approaches, click chemistry has gained considerable attention as an efficient strategy for the functionalization of biologically active molecules [15,16,17]. In particular, the copper(I)-catalyzed azide–alkyne cycloaddition (CuAAC), independently reported by Sharpless and Meldal, enables the regioselective formation of 1,4-disubstituted 1,2,3-triazoles under mild conditions [8,9]. This reaction is distinguished by its high yields, excellent functional group tolerance, operational simplicity, and compatibility with a wide range of solvents, including water. These features make CuAAC an especially valuable tool for late-stage diversification of complex molecular scaffolds [18,19].

**Figure 1:**
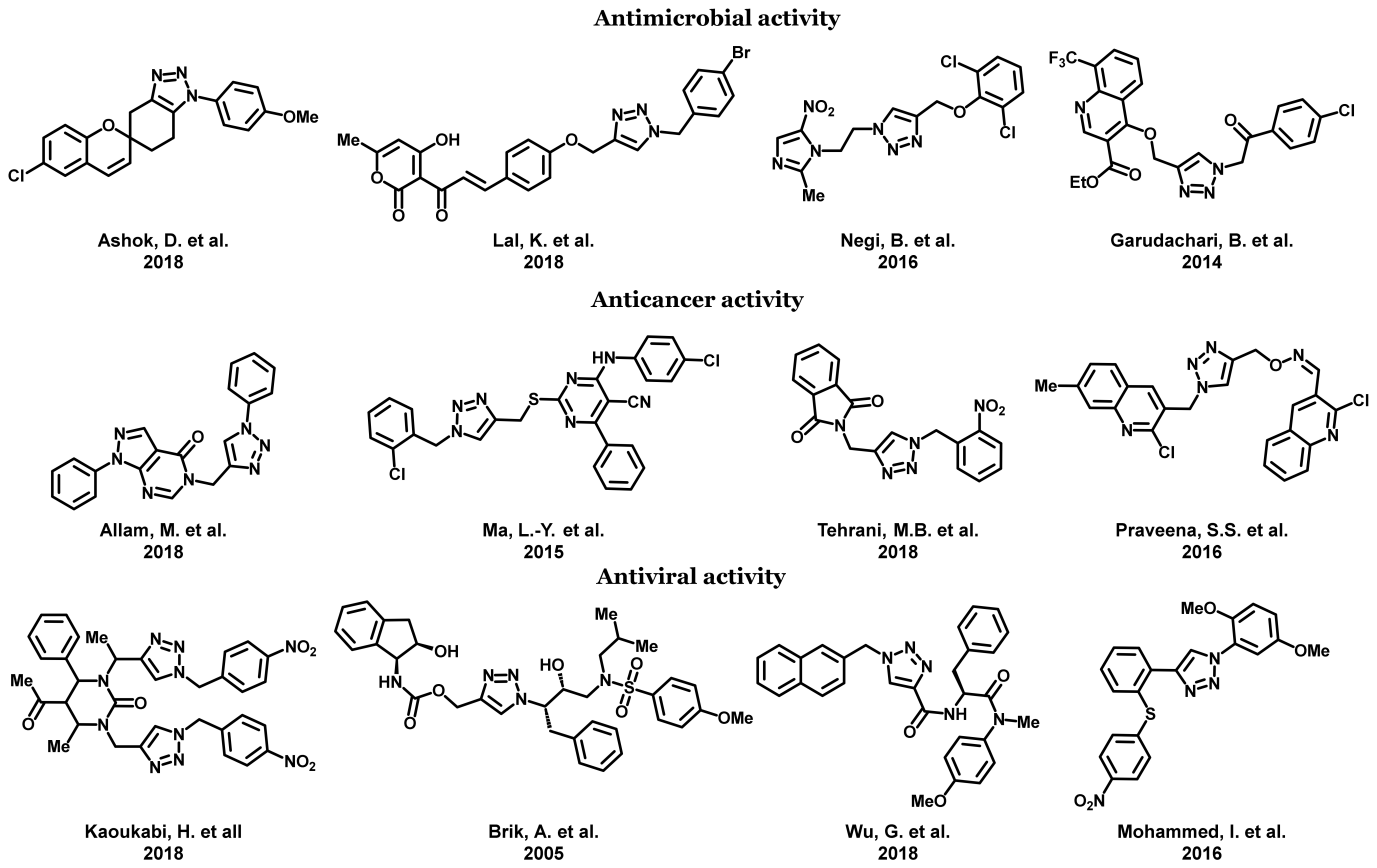
Representative biologically active compounds containing the 1,2,3-triazole scaffold [27,28,29,30,31,32,33,34,35,36,37,38].

The 1,2,3-triazole ring is considered a privileged structural motif in medicinal chemistry because of possible bioorthogonality of its formation [20,21] and its biological activity [22,23]. It is chemically stable, resistant to metabolic degradation, and capable of engaging in hydrogen bonding and dipole–dipole interactions with biological targets [24]. Particularly, the 1,2,3-triazole moiety often acts as a bioisostere for amide or heteroaromatic groups, allowing modulation of electronic properties and molecular conformation without compromising structural integrity [25,26]. Numerous studies have demonstrated that compounds bearing 1,2,3-triazole rings exhibit a broad spectrum of biological activities, including antifungal, antibacterial, antiviral, and anticancer effects (Figure 1).

In this context, the modification of the fluconazole scaffold through the introduction of a 1,2,3-triazole moiety via CuAAC represents a rational and modular synthetic strategy. This approach allows for the incorporation of diverse substituents at a late stage of synthesis while preserving the core pharmacophoric elements responsible for antifungal activity. Such structural diversification provides an opportunity to fine-tune physicochemical properties, explore structure–activity relationships (SAR), and potentially overcome existing resistance mechanisms. Therefore, the present study focuses on the synthesis of novel fluconazole derivatives functionalized with 1,2,3-triazole rings, using CuAAC methodology and their structural characterization as potential antifungal agents.

The obtained derivatives were evaluated for antifungal activity against clinically relevant *Candida* species, including *C. albicans*, *C. parapsilosis*, *C. krusei*, *C. tropicalis*, *C. glabrata*, and *C. lusitaniae* from reference American Type Culture Collection (ATCC) and *C. auris* from the Centers for Disease Control and Prevention (CDC) collection. Some compounds showed comparable activity relative to the parent drug.

## 2. Results and Discussion

### 2.1. Chemistry

**Scheme 1:**
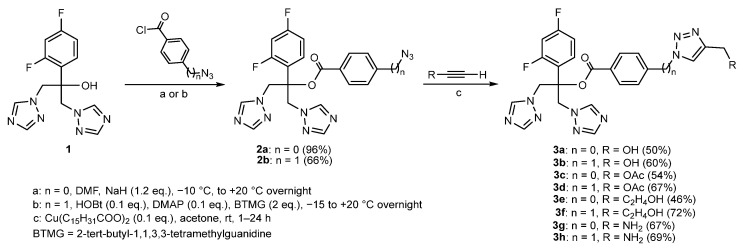
Synthesis of fluconazole derivatives **2** and **3.**

Synthesis of a fluconazole derivative bearing additional heterocyclic ring proceeded in two steps, as depicted in Scheme 1.

To obtain derivatives of fluconazole suitable for subsequent CuAAC transformations several known esterification approaches, run under mild conditions, were assessed. Attempts to employ a carbodiimide-mediated activation in the presence of 1-hydroxybenzotriazole (HOBt) and 4-dimethylaminopyridine (DMAP) [39] did not provide the desired products or result in trace yields (~1%). These outcomes highlighted the low nucleophilicity of the hydroxyl group in fluconazole and necessitated the development of more forcing reaction conditions.

Consequently, a two-step activation strategy was adopted. Fluconazole was first converted into the corresponding alkoxide using sodium hydride in DMF. In parallel, 4-azidobenzoic acid was transformed into the corresponding acid chloride in reaction with thionyl chloride. The acid chloride was generated in situ and used immediately in the subsequent esterification without purification. Under these strongly basic conditions, the esterification proceeded smoothly, affording the desired 4-azidobenzoate derivative in excellent yield of 96%.

The same protocol was initially applied to the synthesis of the fluconazole ester with 4-(azidomethyl)benzoic acid. However, this approach proved ineffective. To overcome this limitation, 2-*tert*-butyl-1,1,3,3-tetramethylguanidine (BTMG) was employed as a strong, non-nucleophilic base instead of sodium hydride. In the synthesis performed in dry THF, the target ester was obtained in a satisfactory yield of 66%.

Eventually, copper-catalyzed azide-alkyne cycloaddition (CuAAC) reactions were performed to access the corresponding 1,2,3-triazole derivatives **3a**–**h**. The usual catalytic system (CuSO_4_/sodium ascorbate) in a 1:1 (*v*/*v*) mixture of water and tert-butanol enabled efficient cycloaddition with propargyl alcohol and pent-4-yn-1-ol. In contrast, no formation of the desired products **3g** and **3h** was observed with propargylamine under these conditions.

To address this limitation, a modified catalytic system was developed based on copper(II) palmitate, prepared according to a modified procedure [40] which was subsequently reduced to Cu(I) palmitate by ascorbic acid added in dry acetone in sealed vial. The reaction was sonicated in an ultrasonic bath at 30 °C until complete disappearance of the characteristic blue color of Cu(II) ions. The resulting off-white suspension containing the required amount of catalyst was transferred via syringe to a separate vial containing the CuAAC substrates dissolved in dry acetone.

Application of this catalytic system resulted in a markedly accelerated reaction rate and improved the yields. Importantly, it enabled successful cycloaddition with propargylamine, affording the previously inaccessible 1,2,3-triazole products **3g**, **3h**.

The structures of the newly obtained compounds were determined by ^1^H NMR, ^13^C NMR, ^19^F spectroscopy, and high-resolution mass spectrometry. These results demonstrate that careful adjustment of activation strategies and enhancement of the nucleophilicity of the oxygen atom of fluconazole as well as the proper selection of the catalytic system are essential for efficient functionalization of fluconazole and its derivatives.

High-resolution mass spectrometry confirmed the expected atomic composition, while the NMR spectroscopy confirmed structures of all investigated compounds. The analysis of the ^1^H NMR and HPLC-HRMS (Supplementary Materials) data demonstrated that all newly synthesized compounds had a purity exceeding 95%.

### 2.2. Bioactivity Studies

#### Antifungal Activity

The antifungal activity of the fluconazole derivatives was evaluated for seven different *Candida* spp. reference strains using the microdilution method in accordance with EUCAST guidelines. As summarized in Table 1, the tested compounds displayed varying antifungal activity with the most favorable activity profiles, with compounds **2a** and **2b** exhibiting the lowest MIC values against *C. albicans* ATCC 10231 (MIC = 1.95 µg/mL), *C. parapsilosis* ATCC 22019 (MIC = 7.81 µg/mL), *C. tropicalis* ATCC 1369 (MIC = 15.625 µg/mL) and *C. lusitaniae* ATCC 3449 (MIC = 0.48 µg/mL). This derivative displayed fungicidal effects against *C. lusitaniae* (MFC = 0.98 µg/mL). The same effect was also observed for **2b** which was fungicidal against *C. lusitaniae* (MFC = 7.81 µg/mL).

Compounds **3b** and **3f** exhibited MIC values of 125 µg/mL for *C. albicans*, *C. parapsilosis* and *C. lusitaniae*, with MIC > 125 µg/mL for the other species and uniformly high MFC values (>125 µg/mL), suggesting weak fungistatic activity at the upper limit of the tested concentration range.

These data indicate that the introduction of a benzoate ester moiety onto the fluconazole scaffold allows the retention of measurable antifungal activity, particularly for **2a** and **2b**; however, the newly synthesized derivatives do not exceed the overall potency of the parent drug across the tested spectrum of *Candida* species. The most pronounced effects were observed against *C. parapsilosis* and *C. lusitaniae*, where several compounds exhibited low MIC values and, in selected cases, fungicidal activity, suggesting species-dependent differences in yeast susceptibility toward the modified molecules.

Preliminary analysis may suggest that both the length of the benzylic spacer and the nature of the terminal substituent play a significant role in modulating biological activity, including antimicrobial activity. Within the studied series, **2a** and **2b** are azide-containing derivatives, where the azide group represents a small, moderately polar functional moiety. In **2b**, the azide is introduced via a methylene linker, whereas in **2a** it is directly attached to the aromatic ring. Notably, **2a** exhibits higher antifungal activity than **2b**, indicating that direct attachment of the azide group to the aromatic core is favorable for biological potency among these compounds. Both compounds consistently show lower MIC values compared to more heavily substituted analogues such as **3b**, **3c**, and **3e**. However, among the compounds bearing a 1,2,3-triazole moiety, **3g** and **3h** exhibited the highest potency. Both compounds feature an amine group attached to the carbon atom adjacent to the 1,2,3-triazole ring. The most active compound in this group possesses a methylene group connecting the core structure to the 1,2,3-triazole ring, which may suggest that this type of linkage modulates the biological activity of the molecule. Furthermore, among the less active derivatives, compounds containing a methylene linker generally exhibited higher activity (**3b** and **3f**) than their analogues lacking this structural feature (**3a** and **3e**), indicating a possible positive contribution of the methylene spacer to biological activity. This trend points to a delicate balance between increased polarity (favouring solubility and hydrogen bonding) and steric bulk (potentially impairing optimal binding to lanosterol 14α-demethylase) as a key factor governing antifungal potency.

The observation that several derivatives retain good activity against *C. parapsilosis* and *C. lusitaniae*, but are markedly less effective toward *C. krusei* and *C. auris*, also indicates a pronounced species-dependent response. This pattern may reflect differences in intrinsic susceptibility, membrane composition, or efflux capacity among *Candida* spp., and suggests that the triazole-modified fluconazole scaffold could be selectively optimized for particular clinical targets.

Fluconazole remains an attractive lead structure for structural optimization because its antifungal mechanism is well established, while multiple studies have shown that chemical modification of the hydroxyl group, aromatic ring, or azole fragments can substantially alter biological activity [9].

In particular, incorporation of a 1,2,3-triazole unit has been widely explored in antifungal medicinal chemistry, both as a metabolically stable linker and as a pharmacophore capable of modulating target binding and physicochemical properties [41]. Previous reports on fluconazole-derived 1,2,3-triazole analogues demonstrated that selected compounds may exhibit activity comparable or even superior to fluconazole against *Candida* spp., although such improvements appear to depend strongly on side-chain architecture and substitution pattern.

The relatively weak activity observed here against *C. krusei* and especially *C. auris* is consistent with the well-recognized reduced azole susceptibility of these species and the high prevalence of fluconazole resistance reported for *C. auris* and *C. lusitaniae* [42].

Although our data are limited to phenotypic MIC and MFC parameters, the current SAR trends are consistent with a scenario in which subtle steric and electronic modifications in the side chain modulate access to, and interactions within, the CYP51 active site, as well as compound permeability and efflux.

### 2.3. Cytotoxic Evaluation

In vitro cytotoxicity of the synthesized compounds was evaluated using the MTT assay on human immortalized keratinocytes (HaCaT) and Chinese hamster lung fibroblasts (V79-4). The obtained results indicate a generally low cytotoxic potential of the tested molecules toward normal cell lines. For the majority of compounds, IC_50_ values exceeded 100 μM after 72 h of exposure, demonstrating limited impact on cell viability within the tested concentration range.

Only two derivatives showed a measurable reduction in cell viability within the tested range, with an IC_50_ value below 100 μM, while all remaining compounds did not reach 50% inhibition even at the highest tested concentration. These findings suggest that the synthesized compounds are well tolerated by non-cancerous cells under the applied experimental conditions.

Overall, the low cytotoxicity observed in both HaCaT and V79-4 cell lines supports a favorable safety profile of the investigated compounds in vitro. When considered alongside previously obtained antifungal activity data, these results may indicate selectivity in their biological effects (Table 2).

## 3. Materials and Methods

### 3.1. Chemistry

#### 3.1.1. General Remarks

All chemicals were purchased from Sigma-Aldrich (Munich, Germany) and used without further purification. ^1^H, ^13^C and ^19^F NMR spectra were recorded on a JEOL ECZL500 (Jeol Ltd., 1-2, Mu-sashino 3-Chome Akishima, Tokyo 196-8558, Japan) in CDCl_3_ as a solvent. The NMR spectra are provided in the Supplementary Materials. IR spectrum was recorded on Nicolet 6700 FT-IR Thermo Scientific, Waltham, MA, USA). The melting points were determined on the Stuart SMP50 melting point apparatus (Cole Parmer Ltd., Stone, UK) and are uncorrected. The purity of the compounds and the reaction progress were tracked by TLC, using an aluminum sheet with 60 F254 plates (Merck Co., Kenilworth, NJ, USA) with a CHCl_3_/MeOH solvent system. The HPLC and MS analyses were performed on a Shimadzu LC-MS Q-Tof 9030 Supplementary Materials (Kyoto, Japan) instrument equipped with a column Dr. Maisch ReproSil-Pur Basic-C18 3 µm 100 mm, which was eluted at 40 °C by MeOH/H_2_O = 70/30 with a flow rate of 0.3 mL/min.

#### 3.1.2. Propargyl Acetate

The synthesis of propargyl acetate was carried out using a modified procedure described in the literature [43].

In a 1 L round-bottom flask, dichloromethane (DCM) (667 mL) was charged with propargyl alcohol (18.5 g, 18 mL, 0.33 mol, 1.0 equiv.), triethylamine (120 g, 167 mL, 1.2 mol, 3.6 equiv.), and 4-dimethylaminopyridine (DMAP) (6.67 g, 0.055 mol, 0.16 equiv.). The reaction mixture was cooled to 0 °C in an ice bath, and acetic anhydride (40 g, 45 mL, 0.36 mol, 1.1 equiv.) was added dropwise with stirring. The reaction mixture was allowed to warm to room temperature and stirred for 40 h. After completion of the reaction, the organic phase was washed successively with water (3 × 500 mL), 0.5 M hydrochloric acid (3 × 500 mL), and brine (3 × 500 mL). The organic layer was dried over anhydrous magnesium sulfate and filtered, and the solvent was removed under reduced pressure. The crude product was purified by vacuum distillation (50 mmHg, b.p. 52 °C). The product was obtained as a colorless liquid, 24.7 g (76%), and its physicochemical and spectral characteristics were identical to those reported in the literature.

#### 3.1.3. Synthesis of 4-Azidobenzoic Acid

The synthesis of 4-azidobenzoic acid was carried out using a modified procedure described in the literature [44].

A three-necked round-bottom flask was charged with H_2_O (425 mL) and cooled in an ice–salt bath. Concentrated HCl (54.5 mL) was then added, followed by *p*-aminobenzoic acid (24 g, 175 mmol). When the temperature of the mixture dropped below 0 °C, a solution of NaNO_2_ (14.5 g, 210 mmol in 24 mL H_2_O) was added dropwise via a dropping funnel, maintaining the temperature below 0 °C throughout the addition. The reaction mixture was stirred for 30 min at a temperature below 0 °C, after which a solution of urea (1 g, 16.65 mmol in 5 mL H_2_O) was added and stirring was continued until gas evolution ceased.

Subsequently, cooled dichloromethane (500 mL) was added to the reaction mixture, and a solution of NaN_3_ (17 g, 262.5 mmol in 50 mL H_2_O) was slowly added under the surface of the liquid using a syringe, keeping the temperature below 0 °C. Vigorous foaming was observed during this step; however, slow addition under the surface with efficient stirring minimized foam accumulation and facilitated phase separation.

After completion of the addition, the reaction mixture was allowed to warm to room temperature and stirred for 24 h. The resulting beige precipitate was collected by filtration, washed thoroughly with water, and dried by lyophilization to afford product.

The product was insoluble in water and only sparingly soluble in dichloromethane. Yield: 25.7 g (90%).

M.p. 179–180 °C, 1H NMR (500 MHz, DMSO-*d*_6_) δ (ppm): 7.16 (d, *J* = 8.7 Hz, 2H, Ar-H), 7.91 (d, *J* = 8.7 Hz, 2H, Ar-H). ^13^C NMR (126 MHz, DMSO-*d*_6_) δ (ppm): 119.2, 127.3, 131.2, 144.0, 166.6.

#### 3.1.4. Synthesis of 4-Azidobenzoyl Chloride

4-azidobenzoic acid (8.15 g, 50 mmol) and three drops of DMF were placed in a 50 mL round-bottom flask. Thionyl chloride (35.4 g/21.7 mL, 300 mmol) was added through a water condenser, and the reaction mixture was heated in an oil bath at 75 °C with stirring for 4 h. After completion, excess thionyl chloride was removed under reduced pressure (10 mmHg) to dryness. The resulting product was used in the subsequent reaction without further purification.

#### 3.1.5. Synthesis of 2-(2,4-Difluorophenyl)-1,3-Di(1H-1,2,4-Triazol-1-Yl)Propan-2-Yl 4-Azidobenzoate (**2a**)

Fluconazole (12.75 g, 41.7 mmol) was placed in a round-bottom flask and dissolved in DMF (200 mL). Sodium hydride (2.0 g, 60% oil dispersion, 50 mmol) was then added. After gas evolution had ceased, the flask was equipped with a septum, thermometer, and balloon, and the reaction mixture was cooled in an ice–salt bath to below −10 °C. 4-azidobenzoyl chloride, prepared as described above, was dissolved in DMF (10 mL), and then added dropwise via syringe, maintaining the temperature below −5 °C. Upon completion of the addition, the reaction mixture was stirred overnight at room temperature.

After completion of the reaction, the solvent (DMF) was removed under reduced pressure using a rotary evaporator (Heidolph Scientific Products GmbH, Schwabach, Germany). The resulting residue was dissolved in chloroform and the organic phase was washed three times with 1 M aqueous sodium carbonate solution, followed by washing with distilled water. The organic layer was dried over anhydrous magnesium sulfate and filtered and the solvent was removed under reduced pressure. The crude product was purified by crystallization from 100 mL of a hexane–acetone mixture (3:1 *v*/*v*). Yield: 16.5 g (96%).

#### 3.1.6. Synthesis of 4-(Azidomethyl)Benzoic Acid

The synthesis of 4-(azidomethyl)benzoic acid was carried out using a modified procedure described in the literature [45].

To a round-bottom flask, we added sodium azide (30.0 g, 400 mmol) and DMF (550 mL). The mixture was cooled to 0 °C using an ice bath, and 4-(chloromethyl)benzoic acid (30 g, 176.5 mmol) was added portion wise with stirring. The reaction mixture was then allowed to warm to room temperature and stirred for 48 h. After completion, 1 M HCl (700 mL) was added. A white precipitate formed, which was collected by vacuum filtration, washed thoroughly with 1 M HCl and then with water, and allowed to air-dry at room temperature to afford the crude product. Yield 27 g (86%).

M.p. 130–131 °C, ^1^H NMR (500 MHz, CDCl_3_) δ (ppm): 4.44 (s, 2H, CH_2_), 7.43 (d, *J* = 8.3 Hz, 2H, Ar-H), 8.13 (d, *J* = 8.3 Hz,2H, Ar-H). ^13^C NMR (126 MHz, DMSO-*d*_6_) δ (ppm): 53.1, 128.4, 129.7, 130.4, 140.6, 167.0.

#### 3.1.7. Synthesis of 4-(Azidomethyl)Benzoyl Chloride

4-(azidomethyl)benzoic acid (5.5 g, 30 mmol) and three drops of DMF were placed in a 50 mL round-bottom flask. Thionyl chloride (20 g/11 mL, 174 mmol) was added through a water condenser, and the reaction mixture was heated in an oil bath at 75 °C with stirring for 4 h. After completion, excess thionyl chloride was removed under reduced pressure (10 mmHg) to dryness, affording the crude product as an amber-red liquid, which was used in the subsequent reaction without further purification.

#### 3.1.8. Synthesis of 2-(2,4-Difluorophenyl)-1,3-Di(1H-1,2,4-Triazol-1-Yl)Propan-2-Yl 4-(Azidomethyl)Benzoate (**2b**)

Fluconazole (6.0 g, 20 mmol) was dissolved in THF (140 mL) in a round-bottom flask. To this solution, HOBt (0.4 g), DMAP (0.4 g) and 2-tert-butyl-1,1,3,3-tetramethylguanidine (6.84 g/8.07 mL, 40 mmol) were added, and the reaction mixture was cooled to −15 °C. The corresponding acid chloride was then added dropwise while maintaining the temperature below −10 °C. During the addition, an orange precipitate began to form. The reaction mixture was allowed to warm to room temperature and stirred overnight.

After completion of the reaction, approximately 80% of THF was removed under reduced pressure. The residue was dissolved in 200 mL of 0.5 M hydrochloric acid, and 300 mL of chloroform was added. The organic phase was washed four times with 0.5 M hydrochloric acid, followed by two washes with 1 M sodium carbonate solution and one wash with brine. The organic layer was then collected, dried over anhydrous magnesium sulfate, and filtered, and the solvent was removed under reduced pressure. The crude product was purified by flash column chromatography using a gradient of CHCl_3_/MeOH from 40:1 to 20:1 (*v*/*v*), affording the desired compound as a white solid. Yield 6 g (66%).

#### 3.1.9. Synthesis of Copper(I) Palmitate

Palmitic acid (11.1 g, 43.3 mmol) was placed in a round-bottom flask and dissolved in 2 M aqueous NaOH (25 mL) by heating. Anhydrous CuSO_4_ (3.5 g, 26.6 mmol) was then added, and the mixture was heated at 75 °C for 30 min, during which a blue precipitate formed. After completion, the reaction mixture was allowed to cool to room temperature. The solid product was collected by vacuum filtration, washed thoroughly with distilled water, and dried to afford copper(II) palmitate as a blue solid (11.0 g, 82.6%). Copper(II) palmitate (573 mg, 1.0 mmol) and ascorbic acid (100 mg, 1.14 mmol) were placed in a sealed vial, and acetone (20 mL) was added. The mixture was sonicated at 30 °C until complete decolorization. The resulting mixture was used as the catalyst without further purification.

#### 3.1.10. Synthesis of 1,2,3-Triazole Derivatives (**3a**–**3h**)

The CuAAC reactions were performed in sealed vials under anhydrous conditions. Either 2-(2,4-difluorophenyl)-1,3-di(1*H*-1,2,4-triazol-1-yl)propan-2-yl- 4-azidobenzoate (**2a**) or 2-(2,4-difluorophenyl)-1,3-di(1*H*-1,2,4-triazol-1-yl)propan-2-yl- 4-(azidomethyl)benzoate (**2b**) was dissolved in dry acetone. The appropriate terminal alkyne was then added in a slight excess, followed by a suspension of the previously prepared copper catalyst in an amount corresponding to 10 mol%, relative to the azide substrate.

The reaction mixtures were stirred magnetically at room temperature for 1 h in the case of derivatives **3a**–**3f** and for 24 h in the case of derivatives **3g** and **3h**. Reaction progress was monitored by thin-layer chromatography (TLC). When complete consumption of the starting material or no further progress was observed, the reaction was terminated. The solvent was removed under reduced pressure, and the crude products were purified by flash column chromatography, initially using chloroform/methanol (40:1, *v*/*v*), followed by chloroform/methanol (20:1, *v*/*v*) to afford the corresponding 1,2,3-triazole.

#### Physicochemical Characterization of Copper(II) Palmitate

Yield: 82.6%

Mp.: 119–120 °C

IR (cm^−1^) KBr: 2914, 1441 (CH aliph), 1583 (C=O).

#### Physicochemical Characterization of Compounds **2a**, **2b**

2-(2,4-difluorophenyl)-1,3-di(1*H*-1,2,4-triazol-1-yl)propan-2-yl-4-azidobenzoate (**2a**)

Yield 96%

Mp.: 151–152 °C

^1^H NMR (500 MHz, CDCl_3_) δ (ppm): 5.26 (s, 4H, CH_2_), 6.72–6.79 (m, 1H, ArH), 6.80–6.93 (m, 2H, ArH), 7.06 (d, *J* = 8.8 Hz, 2H, ArH), 7.84 (s, 2H, ArH), 7.89 (d, *J* = 8.8 Hz, 2H, ArH), 7.94 (s, 2H, ArH).

^13^C NMR (126 MHz, CDCl_3_) δ (ppm): 51.8 (d, *J* = 4.8 Hz), 81.6 (d, *J* = 4.8 Hz), 105.3 (dd, *J* = 27.3, 25.5 Hz), 112.3 (dd, *J* = 21.4, 4.6 Hz), 119.2, 119.8 (dd, *J* = 11.8, 4.6 Hz), 125.3, 128.4 (dd, *J* = 10.0, 4.6 Hz), 131.7, 144.8, 146.1, 152.2, 159.2 (d, *J* = 260.4 Hz), 163.2 (dd, *J* = 253.1, 12.6 Hz), 164.5.

^19^F NMR (471 MHz, CDCl_3_) δ (ppm) −108.2–−108.1 (m, 1F, ArF), −107.23 (m, 1F, ArF).

HRMS (Supplementary Materials): *m*/*z* = calcd. 452.13895 (M^+^H^+^, C_20_H_15_N_9_O_2_F_2_), found. 452.13939, diff. 0.964 ppm.

2-(2,4-difluorophenyl)-1,3-di(1*H*-1,2,4-triazol-1-yl)propan-2-yl-4-(azidomethyl)benzoate (**2b**)

Yield 66%

Mp.: 113–114 °C

^1^H NMR (500 MHz, CDCl_3_) δ (ppm) 4.43 (s, 2H, CH_2_), 5.28 (s, 4H, CH_2_), 6.74–6.82 (m, 1H, ArH), 6.83–6.95 (m, 2H, ArH), 7.36–7.44 (m, 2H, ArH), 7.85 (s, 2H, ArH), 7.88–7.96 (m, 2H, ArH), 7.96 (s, 2H, ArH).

^13^C NMR (126 MHz, CDCl_3_) δ (ppm) 51.8 (d, *J* = 6.0 Hz), 54.2, 81.7 (d, *J* = 4.8 Hz), 105.3 (dd, *J* = 25.2, 25.2 Hz), 112.4 (dd, *J* = 21.6, 2.4 Hz), 119.7 (dd, *J* = 10.8, 3.6 Hz), 128.2, 128.4 (dd, *J* = 9.6, 4.8 Hz), 128.9, 130.3, 141.9, 144.8, 152.2, 159.3 (dd, *J* = 248.4, 10.8 Hz), 163.2 (dd, *J* = 253.2, 13.2 Hz), 164.9.

^19^F NMR (471 MHz, CDCl_3_) δ (ppm) −108.2–−108.1 (m, 1F, ArF), −107.27–−107.13 (m, 1F, ArF).

HRMS (Supplementary Materials): *m*/*z* = calcd. 466.15460 (M^+^H^+^, C_21_H_17_N_9_O_2_F_2_), found. 466.15506, diff. 0.978 ppm.

#### Physicochemical Characterization of Compounds **3a**–**3h**

2-(2,4-difluorophenyl)-1,3-di(1*H*-1,2,4-triazol-1-yl)propan-2-yl-4-(4-(hydroxymethyl)-1*H*-1,2,3-triazol-1-yl)benzoate (**3a**)

Yield: 50%

Mp.: 95–96 °C

^1^H NMR (500 MHz, CDCl_3_) δ (ppm) 4.87 (s, 2H, CH_2_), 5.21–5.34 (m, 4H, CH_2_), 6.75–6.83 (m, 1H, ArH), 6.85–6.95 (m, 2H, ArH), 7.81–7.85 (m, 2H, ArH), 7.85 (s, 2H, ArH), 7.99 (s, 2H, ArH), 8.03–8.10 (m, 3H, ArH).

^13^C NMR (126 MHz, CDCl_3_) δ(ppm) 51.6 (d, *J* = 4.8 Hz), 56.3, 81.8 (d, *J* = 4.8 Hz), 105.4 (dd, *J* = 26.4, 26.4 Hz), 112.4 (dd, *J* = 24.0, 2.4 Hz), 119.5 (dd, *J* = 10.8, 3.6 Hz), 119.7, 120.1, 128.3 (dd, *J* = 9.0, 5.4 Hz), 129.1, 131.6, 140.8, 144.9, 149.1, 152.2, 159.2 (dd, *J* = 249.6, 9.8 Hz), 163.2 (dd, *J* = 254.4, 13.2 Hz), 164.2.

^19^F NMR (471 MHz, CDCl_3_) δ (ppm) −108.27–−108.16 (m, 1F, ArF), −106.84–−106.75 (m, 1F, ArF).

HRMS (Supplementary Materials): *m*/*z* = calcd. 508.16517 (M^+^H^+^, C_23_H_19_N_9_O_3_F_2_), found. 508.16532, diff. 0.299 ppm.

2-(2,4-difluorophenyl)-1,3-di(1*H*-1,2,4-triazol-1-yl)propan-2-yl-4-((4-(hydroxymethyl)-1*H*-1,2,3-triazol-1-yl)methyl)benzoate (**3b**)

Yield: 60%

Mp.: 86–87 °C

^1^H NMR (500 MHz, CDCl_3_) δ (ppm) 4.78 (s, 2H, CH_2_), 5.20–5.33 (m, 4H, CH_2_), 5.58 (s, 2H, CH_2_), 6.73–6.80 (m, 1H, ArH), 6.80–6.94 (m, 2H, ArH), 7.31 (d, *J* = 8.4 Hz, 2H, ArH), 7.50 (s, 1H, ArH), 7.84 (s, 2H, ArH), 7.90 (d, *J* = 8.4 Hz, 2H, ArH), 7.95 (s, 2H, ArH).

^13^C NMR (126 MHz, CDCl_3_) δ (ppm) 51.8 (d, *J* = 4.8 Hz), 53.6, 56.6, 81.8 (d, *J* = 4.8 Hz), 105.5 (dd, *J* = 27.6, 27.6 Hz), 112.4 (dd, *J* = 20.4, 2.4 Hz), 119.6 (dd, *J* = 12.0, 3.6 Hz), 121.8, 128.2, 128.3 (dd, *J* = 9.6, 6.0 Hz), 129.5, 130.6, 140.7, 144.8, 148.4, 152.1, 159.2 (dd, *J* = 248.4, 10.8 Hz), 163.2 (dd, *J* = 253.2, 13.3 Hz), 164.6.

^19^F NMR (471 MHz, CDCl_3_) δ (ppm) −108.19 (dt, *J* = 12.6, 8.8 Hz, 1F, ArF), −107.03 (qd, *J* = 8.8, 6.1 Hz, 1F, ArF).

HRMS (Supplementary Materials): *m*/*z* = calcd. 522.18082 (M^+^H^+^, C_24_H_21_N_9_O_3_F_2_), found. 522.18145, diff. 1.210 ppm.

2-(2,4-difluorophenyl)-1,3-di(1*H*-1,2,4-triazol-1-yl)propan-2-yl-4-(4-(acetoxymethyl)-1*H*-1,2,3-triazol-1-yl)benzoate (**3c**)

Yield: 54%

Mp.: 81–82 °C

^1^H NMR (500 MHz, CDCl_3_) δ (ppm) 2.10 (s, 3H, CH_3_), 5.24–5.35 (m, 6H, CH_2_), 6.77–6.84 (m, 1H, ArH), 6.87–6.96 (m, 2H, ArH), 7.83–7.88 (m, 4H, ArH), 8.00 (s, 2H, ArH), 8.05–8.11 (m, 3H, ArH).

^13^C NMR (126 MHz, CDCl_3_) δ (ppm) 20.9, 51.6 (d, *J* = 4.8 Hz), 57.4, 81.9 (d, *J* = 3.6 Hz), 105.4 (m), 112.5 (m), 119.5 (m), 120.2, 121.9, 128.3 (dd, *J* = 10.8, 4.8 Hz), 129.3, 131.7, 140.7, 144.2, 144.9, 152.3, 159.1 (d, *J* = 246.0 Hz), 163.3 (dd, *J* = 243.6, 12.0 Hz), 164.1, 170.9.

^19^F NMR (471 MHz, CDCl_3_) δ (ppm) −108.23–−108.11 (m, 1F, ArF), −107.04–−106.91 (m, 1F, ArF).

HRMS (Supplementary Materials): *m*/*z* = calcd. 550.17573 (M^+^H^+^, C_25_H_21_N_9_O_4_F_2_), found. 550.17570, diff. −0.060 ppm.

2-(2,4-difluorophenyl)-1,3-di(1*H*-1,2,4-triazol-1-yl)propan-2-yl-4-((4-(acetoxymethyl)-1*H*-1,2,3-triazol-1-yl)methyl)benzoate (**3d**)

Yield: 67%

Mp.: 88–89 °C

^1^H NMR (500 MHz, CDCl_3_) δ (ppm) 2.06 (s, 3H, CH_3_), 5.21 (s, 2H, CH_2_), 5.24–5.34 (m, 4H, CH_2_), 5.57 (s, 2H, CH_2_), 6.74–6.82 (m, 1H, ArH), 6.84–6.93 (m, 2H, ArH), 7.32 (d, *J* = 8.2 Hz, 2H, ArH), 7.55 (s, 1H, ArH), 7.88 (s, 2H, ArH), 7.91 (d, *J* = 8.3 Hz, 2H, ArH), 8.09 (s, 2H, ArH).

^13^C NMR (126 MHz, CDCl_3_) δ (ppm) 20.9, 52.0 (d, *J* = 4.8 Hz), 53.6, 57.5, 81.5 (d, *J* = 4.8 Hz), 105.5 (dd, *J* = 26.4, 26.4 Hz), 112.5 (dd, *J* = 21.6, 2.4 Hz), 119.3 (dd, *J* = 10.8, 3.6 Hz), 123.9, 128.2, 128.3 (m), 129.4, 130.6, 140.6, 143.6, 144.6, 150.9, 159.2 (dd, *J* = 249.6, 12.0 Hz), 163.7 (dd, *J* = 253.2, 13.2 Hz), 164.6, 170.9.

^19^F NMR (471 MHz, CDCl_3_) δ (ppm) −108.27–−108.17 (m, 1F, ArF), −106.84–−106.75 (m, 1F, ArF).

HRMS (Supplementary Materials): *m*/*z* = calcd. 564.19138 (M^+^H^+^, C_26_H_23_N_9_O_4_F_2_), found. 564.19155, diff. 0.296 ppm.

2-(2,4-difluorophenyl)-1,3-di(1*H*-1,2,4-triazol-1-yl)propan-2-yl-4-(4-(3-hydroxypropyl)-1*H*-1,2,3-triazol-1-yl)benzoate (**3e**)

Yield: 46%

Mp.: 85–86 °C

^1^H NMR (500 MHz, CDCl_3_) δ (ppm) 2.00 (tt, *J* = 7.4, 6.2 Hz, 2H, CH_2_), 2.93 (t, *J* = 7.4 Hz, 2H, CH_2_), 3.75 (t, *J* = 6.2 Hz, 2H, CH_2_), 5.22–5.33 (m, 4H, CH_2_), 6.76–6.83 (m, 1H, ArH), 6.86–6.94 (m, 2H, ArH), 7.80–7.85 (m, 3H, ArH), 7.86 (s, 2H, ArH), 7.97 (s, 2H, ArH), 8.03–8.08 (m, 2H, ArH).

^13^C NMR (126 MHz, CDCl_3_) δ (ppm) 22.0, 31.8, 51.6 (d, *J* = 4.8 Hz), 61.7, 81.8 (d, *J* = 4.8 Hz), 105.3 (dd, *J* = 26.4, 26.4 Hz), 112.4 (dd, *J* = 21.6, 3.6 Hz), 118.9, 119.6 (dd, *J* = 11.4, 4.2 Hz), 119.9, 128.3 (dd, *J* = 9.6, 4.8 Hz), 128.8, 131.6, 140.9, 144.9, 149.0, 152.2, 159.2 (dd, *J* = 249.6, 10.8 Hz), 163.2 (dd, *J* = 254.4, 12.0 Hz), 164.3.

^19^F NMR (471 MHz, CDCl_3_) δ (ppm) −108.27–−108.14 (m, 1F, ArF), −106.95–−106.82 (m, 1F, ArF).

HRMS (Supplementary Materials): *m*/*z* = calcd. 536.19647 (M^+^H^+^, C_25_H_23_N_9_O_3_F_2_), found. 536.19664, diff. 0.321 ppm.

2-(2,4-difluorophenyl)-1,3-di(1*H*-1,2,4-triazol-1-yl)propan-2-yl-4-((4-(3-hydroxypropyl)-1*H*-1,2,3-triazol-1-yl)methyl)benzoate (**3f**)

Yield: 72%

Mp.: 79–80 °C

^1^H NMR (500 MHz, CDCl_3_) δ (ppm) 1.87–1.98 (m, 2H, CH_2_), 2.83 (t, *J* = 7.3 Hz, 2H, CH_2_), 3.68 (t, *J* = 6.1 Hz, 2H, CH_2_), 5.21–5.31 (m, 4H, CH_2_), 5.55 (s, 2H, CH_2_), 6.73–6.80 (m, 1H, ArH), 6.80–6.94 (m, 2H, ArH), 7.26 (s, 1H, ArH), 7.29 (d, *J* = 8.3 Hz, 2H, ArH), 7.84 (s, 2H, ArH), 7.89 (d, *J* = 8.3 Hz, 2H, ArH), 7.93 (s, 2H, ArH).

^13^C NMR (126 MHz, CDCl_3_) δ (ppm) 22.1, 31.7, 51.8 (d, *J* = 6.0 Hz), 53.4, 61.8, 81.8 (d, *J* = 4.8 Hz), 105.3 (dd, *J* = 27.6, 27.6 Hz), 112.4 (dd, *J* = 21.4, 2.5 Hz), 119.6 (dd, *J* = 12.0, 4.8 Hz), 121.1, 128.1, 128.3 (dd, *J* = 10.2, 5.4 Hz), 129.3, 130.5, 141.1, 144.8, 148.3, 152.2, 157.2 (dd, *J* = 277.2, 10.8 Hz), 163.2 (dd, *J* = 253.2, 12.0 Hz), 164.8.

^19^F NMR (471 MHz, CDCl_3_) δ (ppm) −108.29–−108.10 (m, 1F, ArF), −107.14–−107.01 (m, 1F, ArF).

HRMS (Supplementary Materials): *m*/*z* = calcd. 550.21212 (M^+^H^+^, C_26_H_25_N_9_O_3_F_2_), found. 550.21251, diff. 0.711 ppm.

2-(2,4-difluorophenyl)-1,3-di(1H-1,2,4-triazol-1-yl)propan-2-yl 4-(4-(aminomethyl)-*1H*-1,2,3-triazol-1-yl)benzoate (**3g**)

Yield: 67%

Mp.: 83–84 °C

^1^H NMR (500 MHz, CDCl_3_) δ (ppm) 4.14 (s, 2H, CH_2_), 5.22–5.32 (m, 4H, CH_2_), 6.76–6.83 (m, 1H, ArH), 6.85–6.95 (m, 2H, ArH), 7.83 (d, *J* = 8.9 Hz, 2H, ArH), 7.86 (s, 2H, ArH), 7.97 (s, 2H, ArH), 8.04 (s, 1H, ArH), 8.05 (d, *J* = 8.9 Hz, 2H, ArH).

^13^C NMR (126 MHz, CDCl_3_) δ (ppm) 37.4, 51.7 (d, *J* = 4.8 Hz), 81.8 (d, *J* = 4.8 Hz), 105.3 (dd, *J* = 26.4, 26.4 Hz), 112.4 (dd, *J* = 21.6, 3.6 Hz), 119.1, 119.7 (dd, *J* = 12.0, 3.6 Hz), 120.0, 128.3 (dd, *J* = 9.6, 6.0 Hz), 128.9, 131.6, 140.9, 144.9, 147.4, 152.3, 161.3 (dd, *J* = 240.0, 13.2 Hz), 161.3 (dd, *J* = 240.0, 13.2 Hz), 164.2.

^19^F NMR (471 MHz, CDCl_3_) δ (ppm) −108.29–−108.13 (m, 1F, ArF), −106.98–−106.81 (m, 1F, ArF).

HRMS (Supplementary Materials): *m*/*z* = calcd. 507.18115 (M^+^H^+^, C_23_H_20_N_10_O_2_F_2_), found. 507.18199, diff. 1.650 ppm.

2-(2,4-difluorophenyl)-1,3-di(1H-1,2,4-triazol-1-yl)propan-2-yl 4-((4-(aminomethyl)-*1H*-1,2,3-triazol-1-yl)methyl)benzoate (**3h**)

Yield: 69%

Mp.: 89–90 °C

^1^H NMR (500 MHz, CDCl_3_) δ (ppm) 3.98 (s, 2H, CH_2_), 5.21–5.31 (m, 4H, CH_2_), 5.57 (s, 2H, CH_2_), 6.73–6.80 (m, 1H, ArH), 6.80–6.95 (m, 2H, ArH), 7.31 (d, *J* = 8.5 Hz, 2H, ArH), 7.39 (s, 2H, ArH), 7.84 (s, 2H, ArH), 7.89 (d, *J* = 8.4 Hz, 2H, ArH), 7.93 (s, 2H, ArH).

^13^C NMR (126 MHz, CDCl_3_) δ (ppm) 37.7, 51.7 (d, *J* = 6.0 Hz), 53.5, 81.8 (d, *J* = 4.8 Hz), 105.3 (dd, *J* = 26.4, 26.4 Hz), 112.4 (dd, *J* = 20.4, 2.4 Hz), 119.6 (dd, *J* = 11.4, 4.2 Hz), 120.8, 128.1, 128.3 (dd, *J* = 9.8, 5.2 Hz), 129.4, 130.5, 140.9, 144.8, 150.4, 152.2, 159.2 (dd, *J* = 249.6, 12.0 Hz), 163.2 (dd, *J* = 253.2, 12.0 Hz), 164.8.

^19^F NMR (471 MHz, CDCl_3_) δ (ppm) −108.43–−108.07 (m, 1F, ArF), −107.21–−106.97 (m, 1F, ArF).

HRMS (Supplementary Materials): *m*/*z* = calcd. 521.19680 (M^+^H^+^, C_24_H_22_N_10_O_2_F_2_), found. 521.19770, diff. 1.721 ppm.

### 3.2. Microbiological Assay

The antifungal activity of the obtained fluconazole derivatives was determined by assessing their ability to inhibit the growth of *Candida* spp. yeast from international culture collections, using the broth microdilution method, in accordance with the European Committee on Antimicrobial Susceptibility Testing (EUCAST) guidelines [46] in Mueller–Hinton broth with 2% glucose [47].

This involved preparing microbial suspensions with a standardized density of 0.5 on the McFarland scale (equivalent to 1.5 × 10^8^ colony-forming units (CFU)/mL). Next, a solution of the test substances was prepared with an initial concentration of 125 µg/mL and diluted in sterile Mueller–Hinton broth with 2% glucose. The test was performed in 96-well, flat-bottomed polystyrene plates (Medlab, Poland), with 100 µL of sterile microbiological medium added to each well. The first wells were filled with 200 µL of the test substance diluted to the initial concentration. Then, double dilutions were performed under aseptic conditions using an automatic pipette (Eppendorf SE, Hamburg, Germany). The final step was to add a 10-fold diluted microbial suspension in a volume of 2 µL. The following controls were included at the same time: (a) a medium control (100 µL of sterile medium without the addition of microorganisms); (b) a microorganism viability control (without the addition of the test compound); (c) a test substance control (dilutions of each fluconazole derivative without the addition of microorganisms); and (d) a positive control of the clinically used drug, fluconazole.

The prepared plates were incubated for 18–24 h at 35 ± 2 °C, which was optimal for the growth of the microorganisms under study. After incubation, the spectrophotometric absorbance at 570 nm was measured for each well on the plate. This part of the study aimed to determine two parameters: the minimum inhibitory concentration (MIC) and the minimum fungicidal concentration (MFC) of the tested compound. The MIC value was determined in the well where the absorbance was significantly lower than in the next lower-concentration well. The MFC value was determined by adding 5 µL of the contents of each well to a sterile solid MHA + 2% glucose medium for fungi, respectively. The area on the plate where 99.9% inhibition of microorganism growth was observed indicated the lethal concentration. Additionally, the MFC/MIC ratio could be determined to evaluate the nature of the test substance’s action. If the MFC:MIC ratio was less than 4, the test substance had a fungicidal effect; if the ratio was equal to or greater than 4, the effect was fungistatic. Fluconazole (Glentham Life Sciences, Corsham, UK) was used as a control drug in this test.

### 3.3. In Vitro Cytotoxicity Determination

#### 3.3.1. Cell Viability Assay

Compounds **2a**, **2b** and **3a**–**3h** were tested on the human immortalized keratinocyte cell line (HaCaT) and Chinese hamster lung fibroblasts (V79-4). All the cell lines were purchased from the American Type Culture Collection (ATCC, Manassas, VA, USA) and cultured in strictly defined conditions according to the recommended ATCC protocol. Cells were cultured in Dulbecco’s Modified Eagle Medium (DMEM) supplemented with 10% fetal bovine serum (FBS), penicillin (100 U/mL), and streptomycin (100 μg/mL) and maintained at 37 °C in a humidified incubator with 5% CO_2_. Cells were passaged at 80–90% confluence using 0.25% trypsin (Gibco Life Technologies, Thermo Fisher Scientific, Waltham, MA, USA) and subsequently used for experiments.

#### 3.3.2. MTT Assay

Cell viability was assessed using the MTT assay, based on the conversion of MTT salt [3-(4,5-dimethylthiazol-2-yl)-2,5-diphenyltetrazolium bromide] to insoluble formazan crystals by mitochondrial dehydrogenases in metabolically active cells. Cells were seeded in 96-well plates at a density of 1 × 10^4^ cells per well and allowed to adhere for 24 h at 37 °C in a CO_2_ incubator. After this period, the culture medium was replaced with fresh medium, and cells were treated with various concentrations of the tested compounds (1–100 μM) for 72 h under the same conditions. Compounds were dissolved in dimethyl sulfoxide (DMSO) (Sigma-Aldrich, St. Louis, MO, USA). Untreated cells served as the control. Following treatment, MTT solution (0.5 mg/mL in serum-free medium) was added, and the cells were incubated for an additional 3 h at 37 °C. The medium was then removed, and the formed formazan crystals were dissolved in an isopropanol–DMSO mixture (1:1, *v*/*v*). Absorbance was measured at 570 nm using a BioTek Synergy H1 microplate reader (BioTek Instruments, USA). The IC_50_ values were calculated using Prism software (version 9.1.0).

## 4. Conclusions

In this study, a series of fluconazole-derived compounds bearing benzoate ester linkers and 1,2,3-triazole moieties was successfully synthesized via optimized esterification and CuAAC strategies, including the development of an efficient catalytic system enabling access to previously unattainable derivatives. Structural confirmation was achieved by ^1^H NMR, ^13^C NMR, ^19^F NMR, and HRMS, with all compounds obtained at high purity.

Biological evaluation demonstrated that some of the investigated derivatives retained antifungal activity, exhibiting a pronounced species-dependent profile. In particular, compounds **2a** and **2b** showed relatively low MIC values compared to the more heavily substituted analogues. The introduction of an additional 1,2,3-triazole ring resulted in most compounds becoming inactive, with compounds **3b, 3f, 3g** and **3h** retaining measurable antifungal activity. Importantly, all compounds, including the active derivatives, displayed low cytotoxicity toward HaCaT and V79-4 cell lines in the MTT assay, indicating a favorable in vitro safety profile.

The observed structure–activity relationships suggest that both the nature of the terminal substituent and the length and type of the linker substantially influence biological activity. In particular, the presence of a methylene spacer between the fluconazole core and the 1,2,3-triazole ring appeared to positively affect activity modulation. Although the introduction of additional 1,2,3-triazole fragments did not lead to a marked enhancement of antifungal potency, the obtained results indicate that this modification strategy remains a viable approach for tuning the biological properties of fluconazole derivatives.

Collectively, these findings support the 1,2,3-triazole-modified fluconazole scaffold as a promising platform for further optimization, particularly toward the development of selective antifungal agents with improved safety and activity profiles against clinically relevant *Candida* species.

## Figures and Tables

**Table 1:** Microbiological parameters defining antifungal activity of the fluconazole derivatives.

Comp.	Antimicrobial Parameters [µg/mL] MIC ^c^ MFC ^d^ (+) Fungicidal/(−) Fungistatic Activity						
	*C. albicans* ATCC ^a^ 10231	*C. parapsilosis* ATCC 22019	*C. krusei* ATCC 14243	*C. auris* CDC ^b^ B11903	*C. tropicalis* ATCC 1369	*C. glabrata* ATCC 15126	*C. lusitaniae* ATCC 3449
**2a**	1.95	7.81	>125	>125	15.625	31.25	0.48
	>125	125	>125	>125	>125	>125	0.98
	n.d.	−	n.d.	n.d.	−	−	+
**2b**	125	62.5	>125	>125	>125	>125	1.95
	>125	>125	>125	>125	>125	>125	7.81
	n.d.	n.d.	n.d.	n.d.	n.d.	n.d.	+
**3a**	>125	>125	>125	>125	>125	>125	>125
	>125	>125	>125	>125	>125	>125	>125
	n.d.	n.d.	n.d.	n.d.	n.d.	n.d.	n.d.
**3b**	125	125	>125	>125	>125	>125	125
	>125	>125	>125	>125	>125	>125	>125
	n.d.	n.d.	n.d.	n.d.	n.d.	n.d.	n.d.
**3c**	>125	>125	>125	>125	>125	>125	>125
	>125	>125	>125	>125	>125	>125	>125
	n.d.	n.d.	n.d.	n.d.	n.d.	n.d.	n.d.
**3d**	>125	>125	>125	>125	>125	>125	>125
	>125	>125	>125	>125	>125	>125	>125
	n.d.	n.d.	n.d.	n.d.	n.d.	n.d.	n.d.
**3e**	>125	>125	>125	>125	>125	>125	125
	>125	>125	>125	>125	>125	>125	>125
	n.d.	n.d.	n.d.	n.d.	n.d.	n.d.	n.d.
**3f**	125	125	>125	>125	>125	>125	125
	>125	>125	>125	>125	>125	>125	>125
	n.d.	n.d.	n.d.	n.d.	n.d.	n.d.	n.d.
**3g**	125	125	>125	>125	>125	>125	62.5
	>125	>125	>125	>125	>125	>125	>125
	n.d.	n.d.	n.d.	n.d.	n.d.	n.d.	n.d.
**3h**	31.25	125	>125	>125	>125	>125	31.25
	>125	>125	>125	>125	>125	>125	62.5
	n.d.	n.d.	n.d.	n.d.	n.d.	n.d.	+
**1 ^e^**	0.98	0.98	31.25	0.97	3.91	3.91	0.122
	>125	3.91	125	>125	125	>125	0.122
	n.d.	+	+	−	−	−	+

Explanatory notes: ^a^ ATCC—American Type Culture Collection, ^b^ CDC—Centers for Disease Control and Prevention, ^c^ MIC—minimum inhibitory concentration [µg/mL], ^d^ MFC—minimum fungicidal concentration [µg/mL], ^e^ **1**—fluconazole.

**Table 2:** Cytotoxic activity (IC_50_ µM/µg/mL) of the studied compounds estimated by the MTT assay.

Compound	HaCaT ^a^ IC_50_^b^ [µM]/[µg/mL]	V79-4 ^c^ IC_50_ [µM]/[µg/mL]
**2a**	95.64/43.1	>100/>43.1
**2b**	88.58/41.2	96.31/44.8
**3a**	>100/>50.7	>100/>50.7
**3b**	>100/>52.1	>100/>52.1
**3c**	>100/>53.5	>100/>53.5
**3d**	>100/>54.9	>100/>54.9
**3e**	>100/>54.9	>100/>54.9
**3f**	>100/>56.3	>100/>56.3
**3g**	>100/>50.6	>100/>50.6
**3h**	>100/>52.0	>100/>52.0
**1 ^d^**	>100/>30.7	>100/>30.7

^a^ Human immortal keratinocyte cell line from adult human skin (HaCaT). ^b^ IC_50_ (µM/µg/mL)—the concentration of the compound that corresponds to 50% growth inhibition of the cell line (compared to the control) after culturing the cells for 72 h with the individual compound. ^c^ Chinese hamster lung fibroblasts. ^d^ Reference compound.

## Data Availability

The original contributions presented in this study are included in the article/Supplementary Materials. Further inquiries can be directed to the corresponding author(s).

## Supplementary Materials

The following supporting information can be downloaded at: https://www.mdpi.com/article/10.3390/molecules31142545/s1: ^1^H NMR, ^13^C NMR, ^19^F NMR and MS spectra for all compounds.
