# Supplementary material for: Click Chemistry Approach to Derivatisation of Fluconazole
Source: Molecules. 2026 Jul 22;31(14):2545. doi: 10.3390/molecules31142545 (PMC13415025; doi:10.3390/molecules31142545)
Supplement: Supplementary file 1 [file molecules-31-02545-s001.zip › molecules-4404980-supplementary.pdf]

Supplementary Materials

Click Chemistry Approach to Derivatisation of Fluconazole

Michał Janowski [1,*], Oleg M. Demchuk [2,*], Sylwia Andrzejczuk [3], Angelika Pawlicka[4], Urszula Kosikowska[3], Marta Struga[4], Marcin Feldo[5], Monika Wujec[6]

[1]Doctoral School, Medical University of Lublin, Chodzki 7, 20-093 Lublin, Poland
[2]Faculty of Medicine, The John Paul II Catholic University of Lublin, Konstantynów 1J, 20-708 Lublin, Poland
[3]Department of Pharmaceutical Microbiology, Faculty of Pharmacy, Medical University, 1 Chodzki Str., 20-093 Lublin, Poland
[4]Chair and Department of Biochemistry, Faculty of Medicine, Banacha Str. 1, 02-097 Warsaw, Poland
[5]Department of Vascular Surgery, Medical University of Lublin, Staszica 11 St., 20-081 Lublin, Poland
[6]Department of Organic Chemistry, Faculty of Pharmacy, Medical University, 4a Chodzki Str., 20-093 Lublin, Poland

* Author to whom correspondence should be addressed. (jjjmichal1@gmail.com)

Table of contents:

S1. 2a - Spectral data 2
S2. 2b - Spectral data 9
S3. 3a - Spectral data 16
S4. 3b - Spectral data 23
S5. 3c - Spectral data 30
S6. 3d - Spectral data 37
S7. 3e - Spectral data 44
S8. 3f - Spectral data 51
S9. 3g - Spectral data 58
S10. 3h - Spectral data 65
S11. Azidobenzoic acid - Spectral data 65
S12. 4-(azidomethyl)benzoic acid - Spectral data 65

S1. 2a

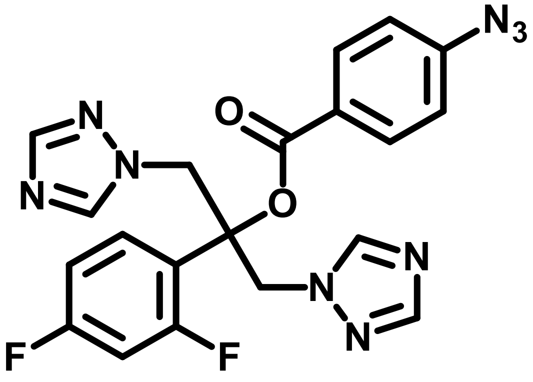

2-(2,4-difluorophenyl)-1,3-di(1H-1,2,4-triazol-1-yl)propan-2-yl 4-azidobenzoate

Figure S1: LC-MS (ESI)

Figure S2: HRMS (ESI), calc/found m/z, Δ ppm

Figure S3: ^{1}H NMR (500 MHz, $CDCl_3$)

Figure S4: ^{13}C NMR (125 MHz, $CDCl_3$)

Figure S5: DEPT-135 (125 MHz, $CDCl_3$)

Figure S6: ^{19}F NMR (471 MHz, $CDCl_3$)

Analysis Report

SHIMADZU LabSolutions

Sample Name	: MJ32		
Sample ID	:		
Data Filename	: MJ32_MeOH_70-15m-03_(150-1500da)_18-02-2026_3.lcd		
Method Filename	: MeOH_70-15m-03_(150-1500da).lcm		
Batch Filename	: 18-02-2026.lcb		
Vial #	: 3-7	Sample Type	: Unknown
Injection Volume	: 0.1 uL		
Date Acquired	: 2/18/2026 1:53:27 PM	Acquired by	: System Administrator
Date Processed	: 2/18/2026 2:08:28 PM	Processed by	: System Administrator

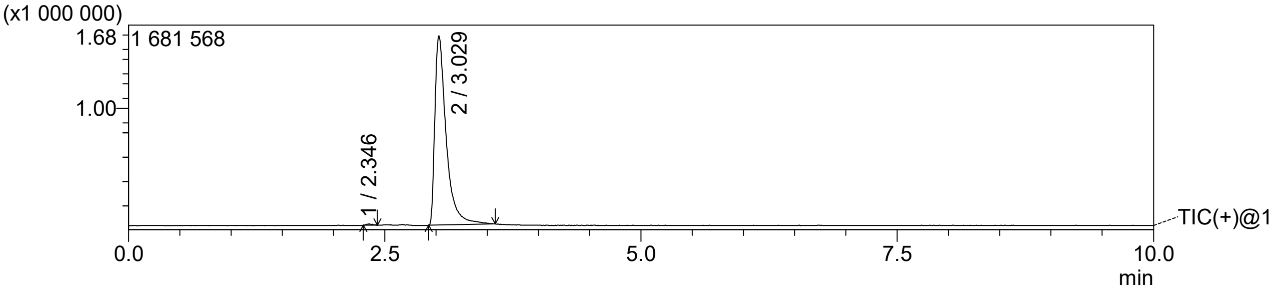

MASS Peak Table TIC

Peak#	Ret. Time	m/z	Area%
1	2.346	TIC	0.353
2	3.029	TIC	99.647
Total			100.000

MS Spectrum

Line#:1 R.Time:----(Scan#:----)
MassPeaks:206
Spectrum Mode:Averaged 3.020-3.030(605-607) Base Peak:452(719488)
BG Mode:Calc Segment 1 - Event 1

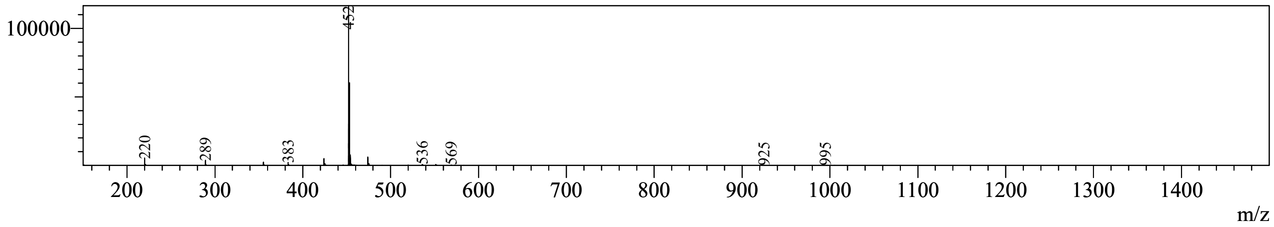

Figure S1. LC-MS (ESI)

Formula Predictor Report

Printed at 19.02.2026 14:09:58

Formula Predictor Result	**C20 H15 N9 O2 F2**
Mass	452.13939
Error Margin	60 ppm
DBE Range	Not Used
Electron Ions	Both configurations
HC Ratio	Not Used
Nitrogen Rule	Used

#	Score	Pred. (M)	Pred. m/z	Meas. m/z	Diff. (mDa)	Formulae (M)	Ion	Diff. (ppm)	Iso Score	DBE
1	97.55	451.13168	452.13895	452.13939	0.44	C20 H15 N9 O2 F2	[M+H]+	0.965	97.28	17.0

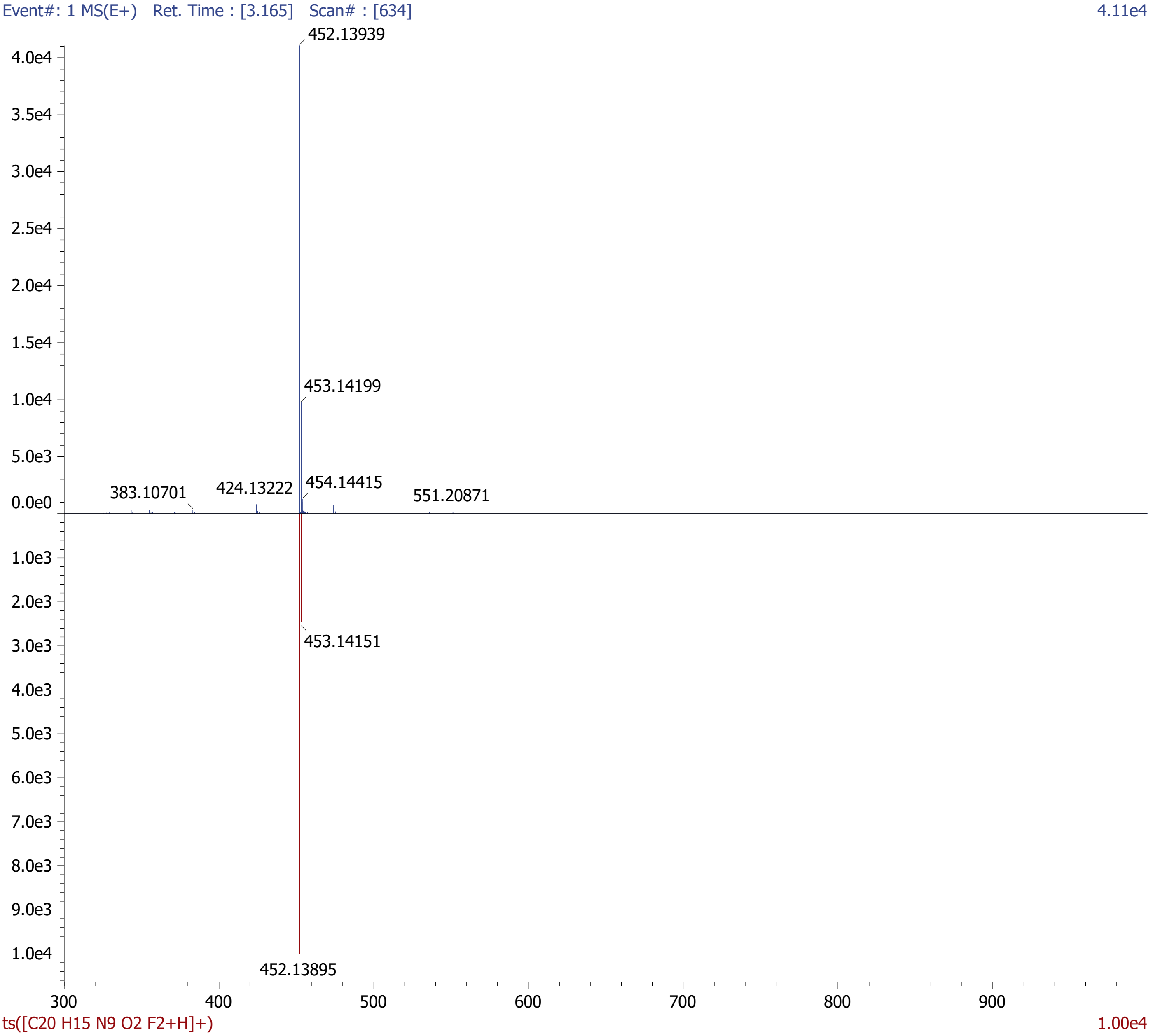

Figure S2. HRMS (ESI), calc/found m/z, Δ ppm

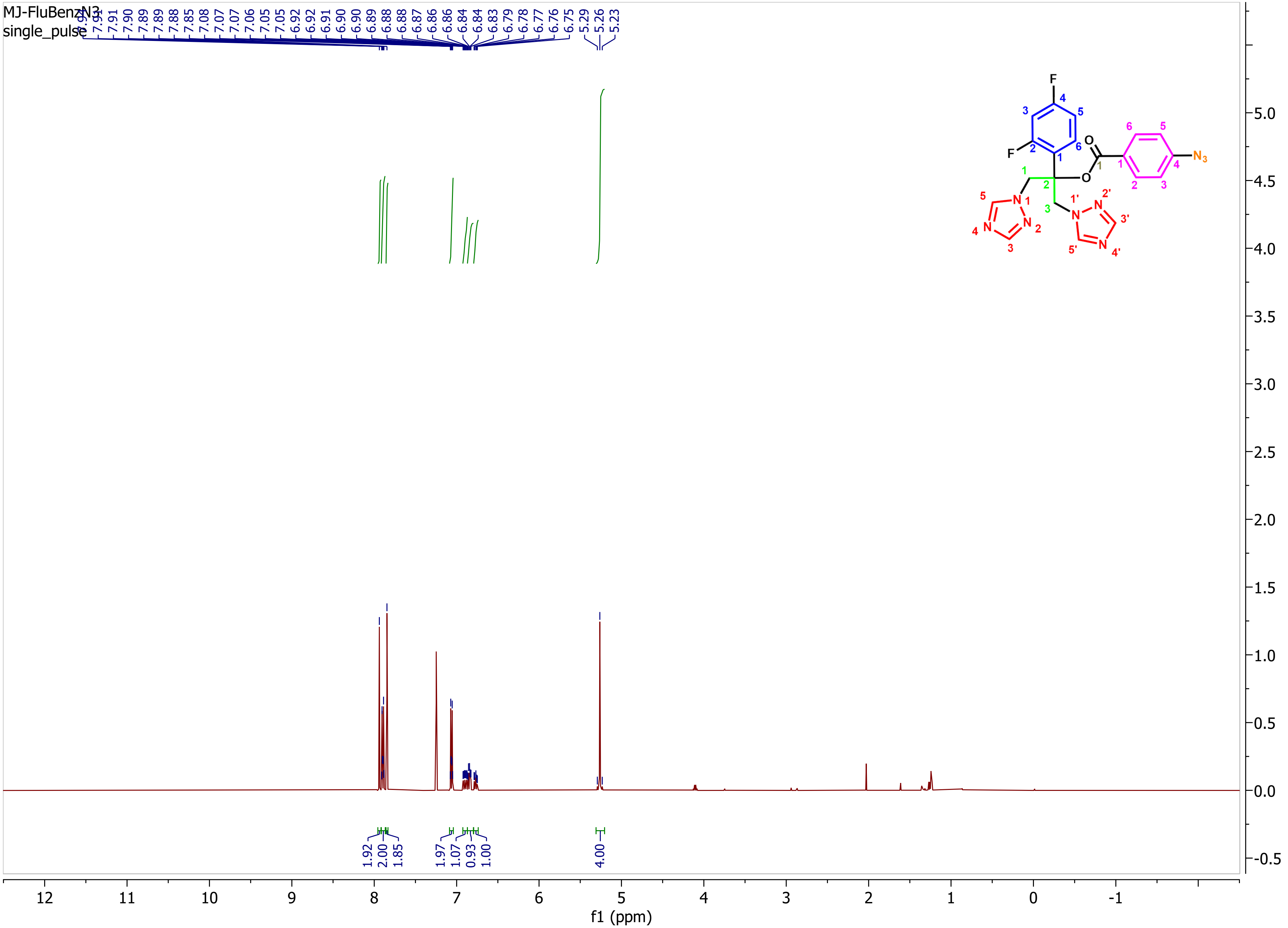

Figure S3. ^{1}H NMR (500 MHz, $CDCl_3$)

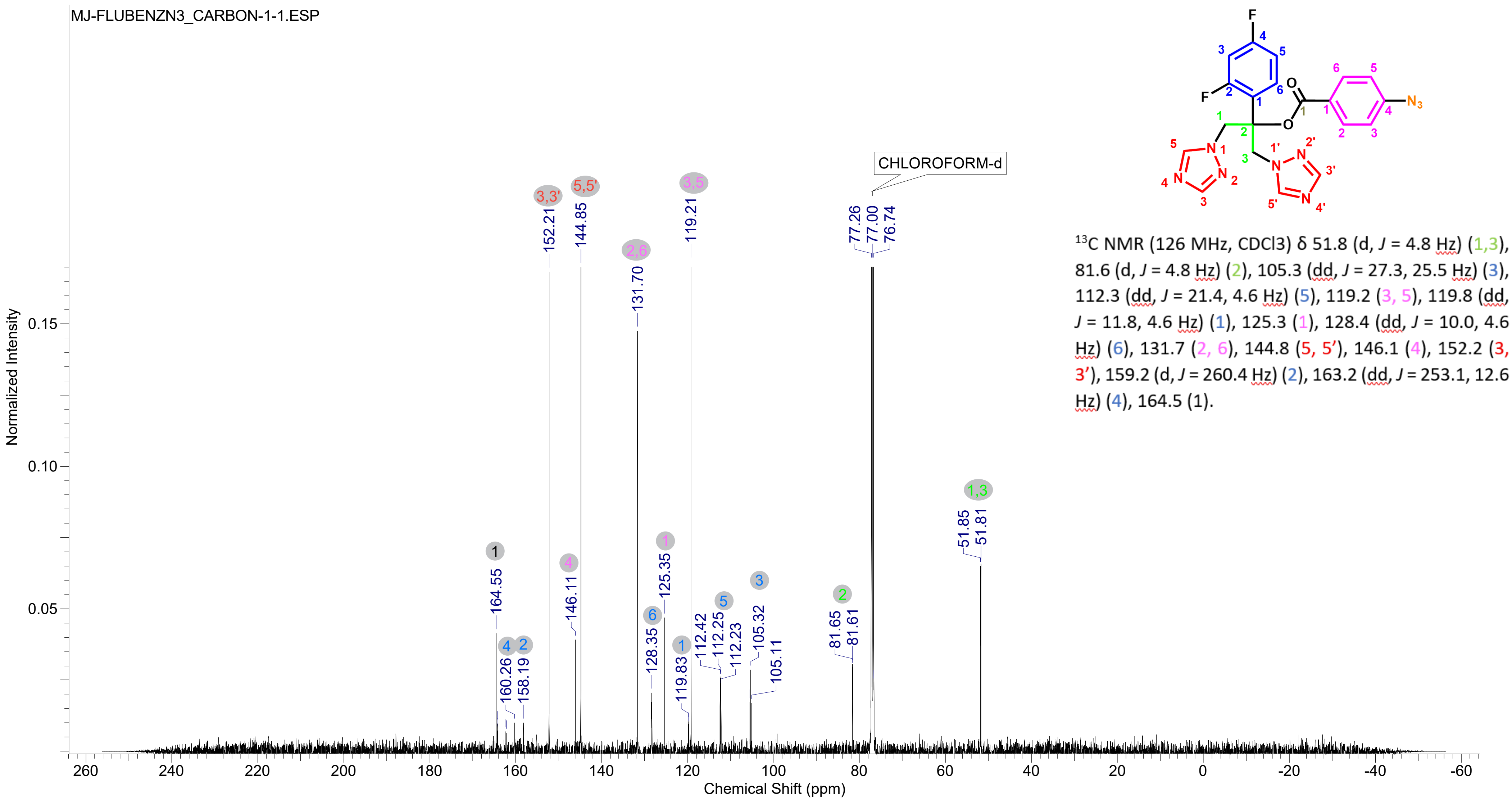

^{13}C NMR (126 MHz, $CDCl_3$) δ 51.8 (d, J = 4.8 Hz) (1,3), 81.6 (d, J = 4.8 Hz) (2), 105.3 (dd, J = 27.3, 25.5 Hz) (3), 112.3 (dd, J = 21.4, 4.6 Hz) (5), 119.2 (3, 5), 119.8 (dd, J = 11.8, 4.6 Hz) (1), 125.3 (1), 128.4 (dd, J = 10.0, 4.6 Hz) (6), 131.7 (2, 6), 144.8 (5, 5'), 146.1 (4), 152.2 (3, 3'), 159.2 (d, J = 260.4 Hz) (2), 163.2 (dd, J = 253.1, 12.6 Hz) (4), 164.5 (1).

Figure S4. ^{13}C NMR (125 Mhz, $CDCl_3$)

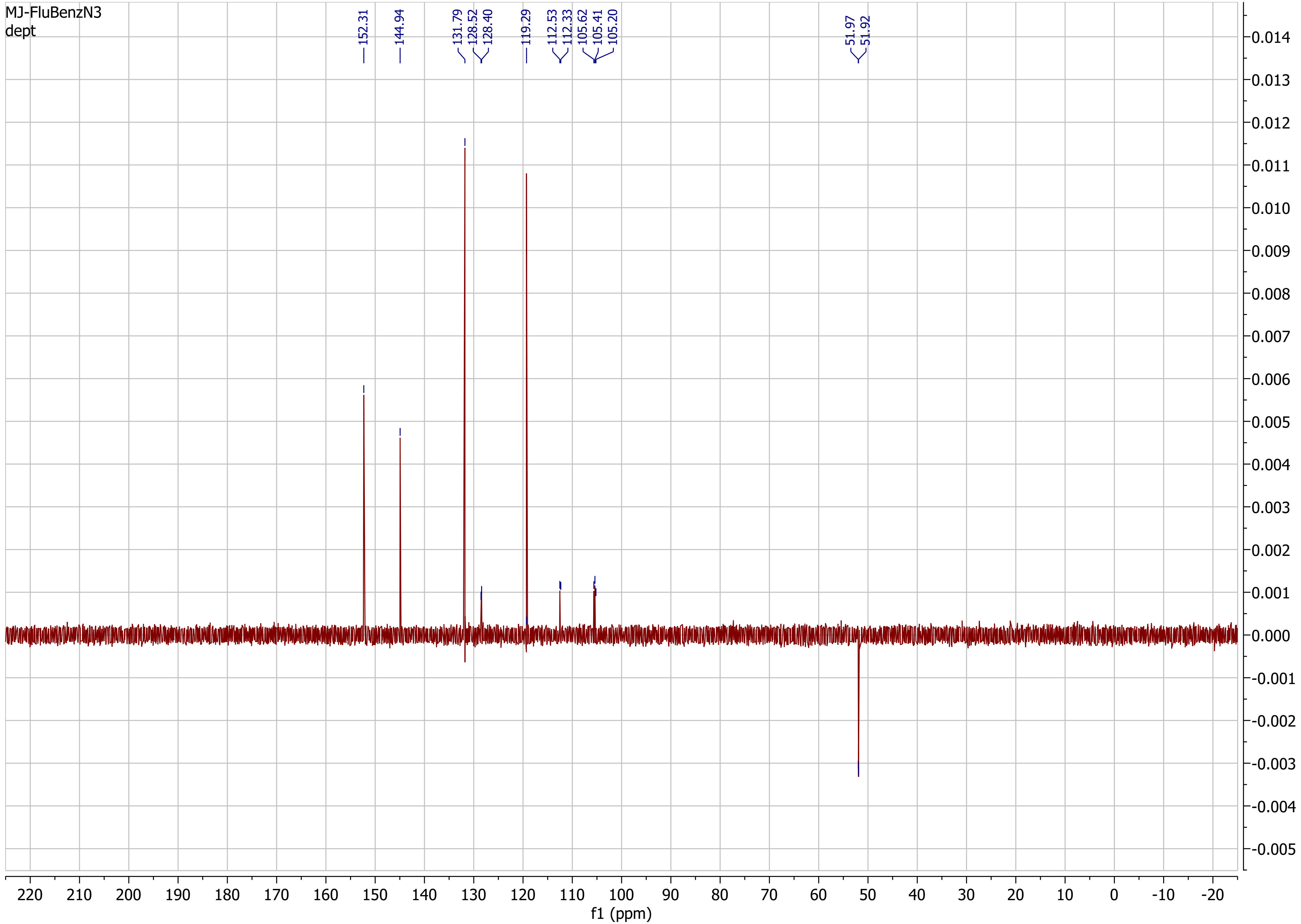

Figure S5. DEPT-135 (125 MHz, $CDCl_3$)

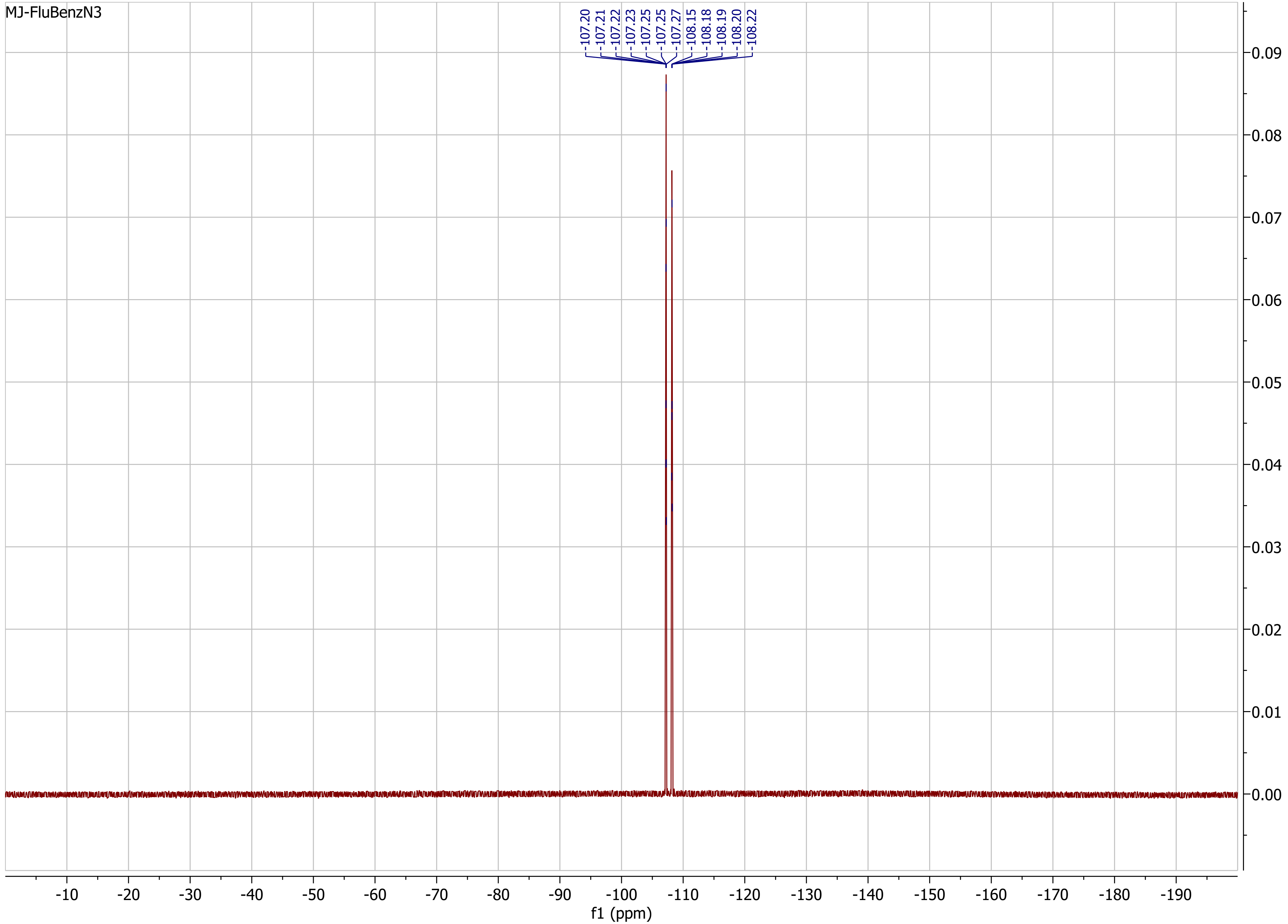

Figure S6. ^{19}F NMR (471 MHz, CDCl3)

S2. 2b

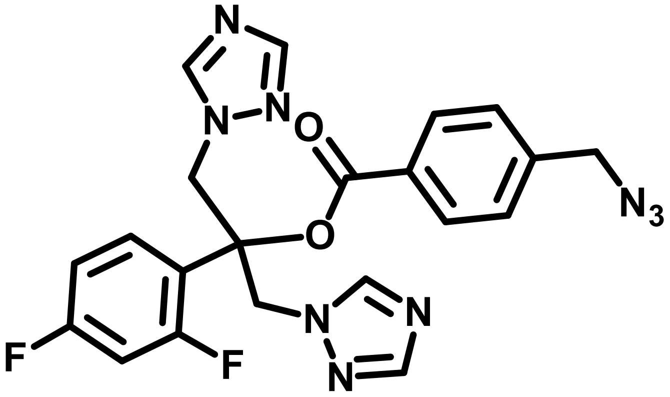

2-(2,4-difluorophenyl)-1,3-di(1H-1,2,4-triazol-1-yl)propan-2-yl 4-(azidomethyl)benzoate

Figure S7: LC-MS (ESI)

Figure S8: HRMS (ESI), calc/found m/z, Δ ppm

Figure S9: ^{1}H NMR (500 MHz, $CDCl_3$)

Figure S10: ^{13}C NMR (125 MHz, $CDCl_3$)

Figure S11: DEPT-135 (125 MHz, $CDCl_3$)

Figure S12: ^{19}F NMR (471 MHz, $CDCl_3$)

SHIMADZU LabSolutions

Analysis Report

Sample Name	: MJ90		
Sample ID	:		
Data Filename	: MJ90_MeOH_70-15m-03_(150-1500da)_18-02-2026_6.lcd		
Method Filename	: MeOH_70-15m-03_(150-1500da).lcm		
Batch Filename	: 18-02-2026.lcb		
Vial #	: 3-10	Sample Type	: Unknown
Injection Volume	: 0.1 uL		
Date Acquired	: 2/18/2026 2:39:44 PM	Acquired by	: System Administrator
Date Processed	: 2/18/2026 2:54:45 PM	Processed by	: System Administrator

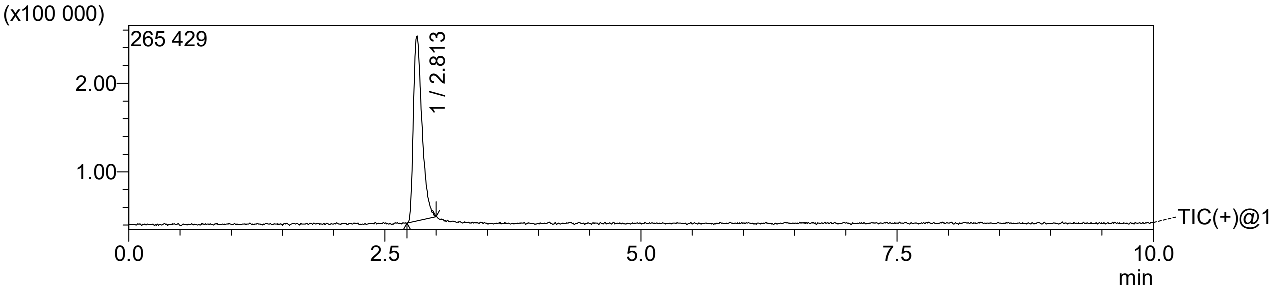

MASS Peak Table TIC

Peak#	Ret. Time	m/z	Area%
1	2.813	TIC	100.000
Total			100.000

MS Spectrum

Line#:1 R.Time:----(Scan#:----)
MassPeaks:75
Spectrum Mode:Averaged 2.805-2.815(562-564) Base Peak:466(131749)
BG Mode:Calc Segment 1 - Event 1

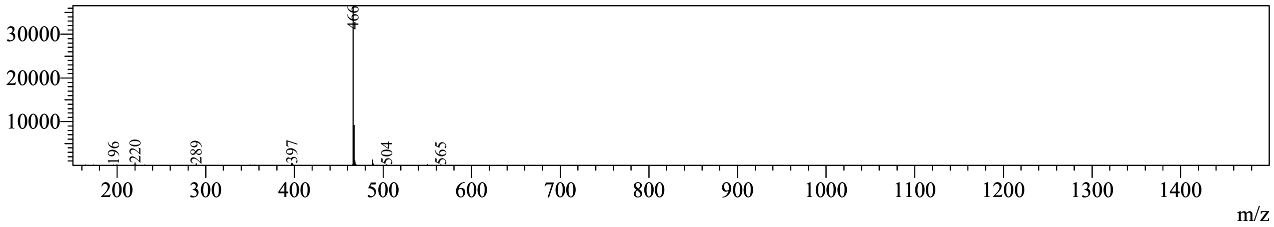

Figure S7. LC-MS (ESI)

Formula Predictor Report

Printed at 19.02.2026 14:18:44

Formula Predictor Result	**C21 H17 N9 O2 F2**
Mass	466.15506
Error Margin	60 ppm
DBE Range	Not Used
Electron Ions	Both configurations
HC Ratio	Not Used
Nitrogen Rule	Used

#	Score	Pred. (M)	Pred. m/z	Meas. m/z	Diff. (mDa)	Formulae (M)	Ion	Diff. (ppm)	Iso Score	DBE
1	86.14	465.14733	466.15460	466.15506	0.46	C21 H17 N9 O2 F2	[M+H]+	0.979	95.71	17.0

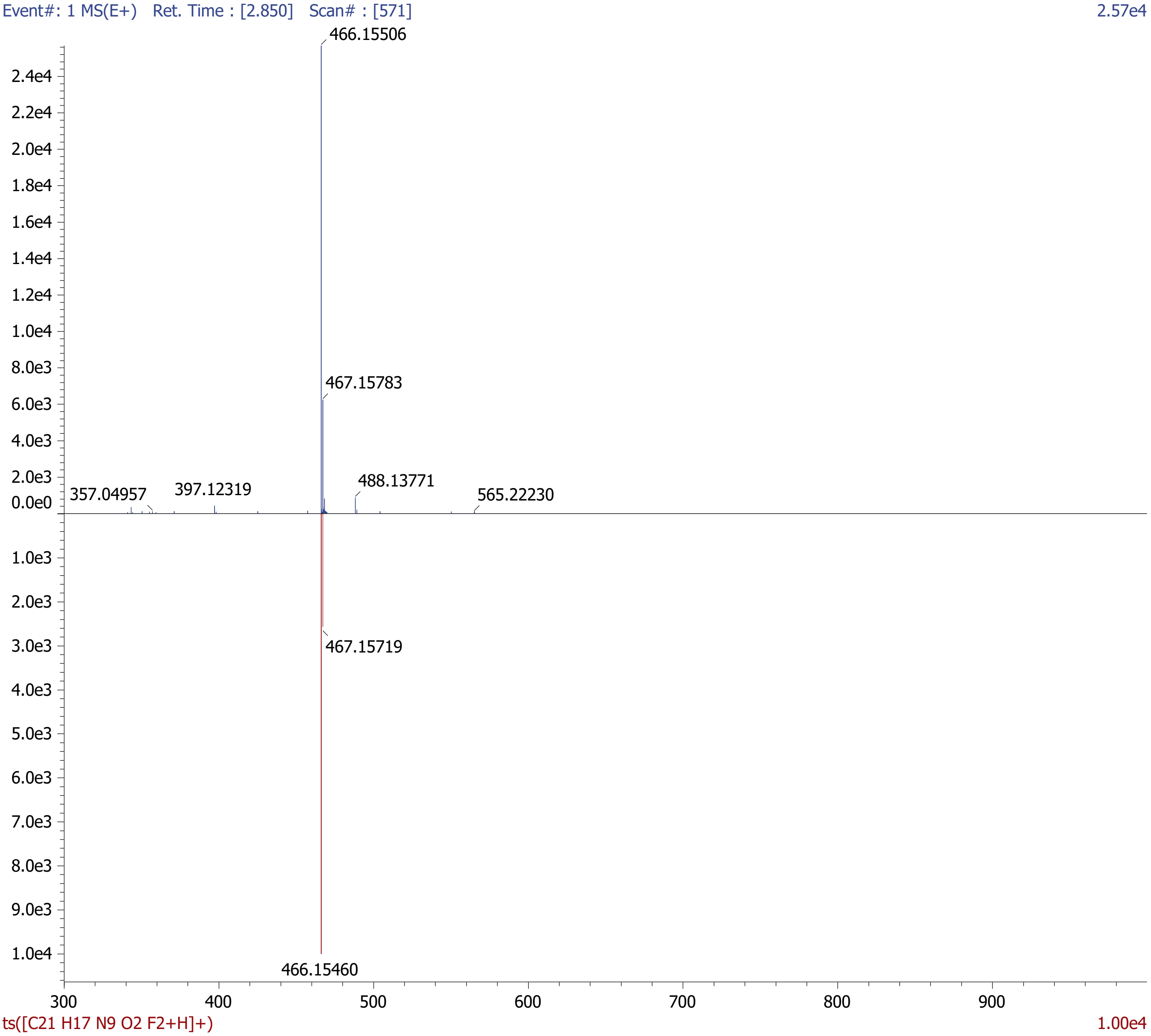

Figure S8. HRMS (ESI), calc/found m/z, Δ ppm

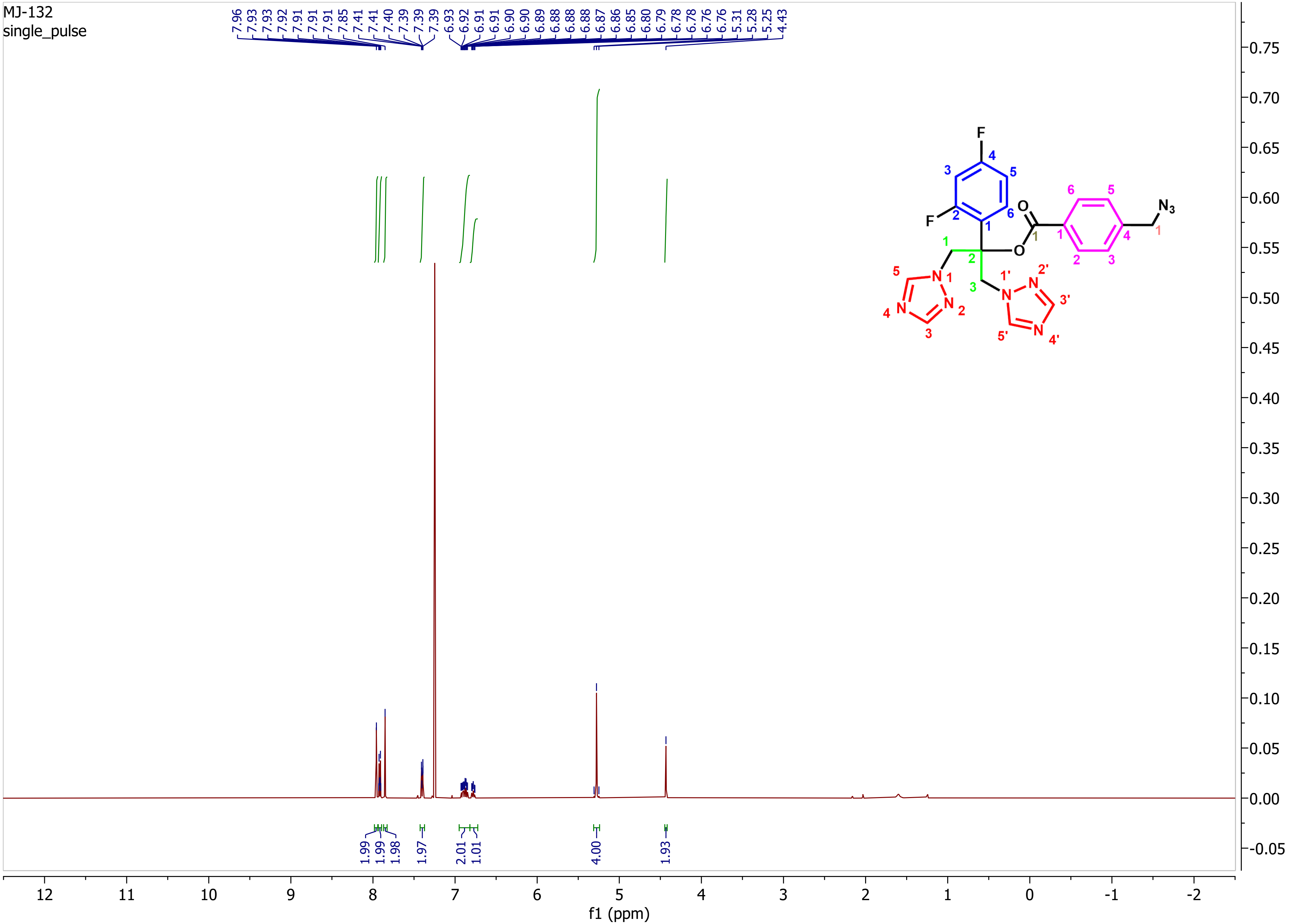

Figure S9. 1HNMR (500 MHz, CDCl3)

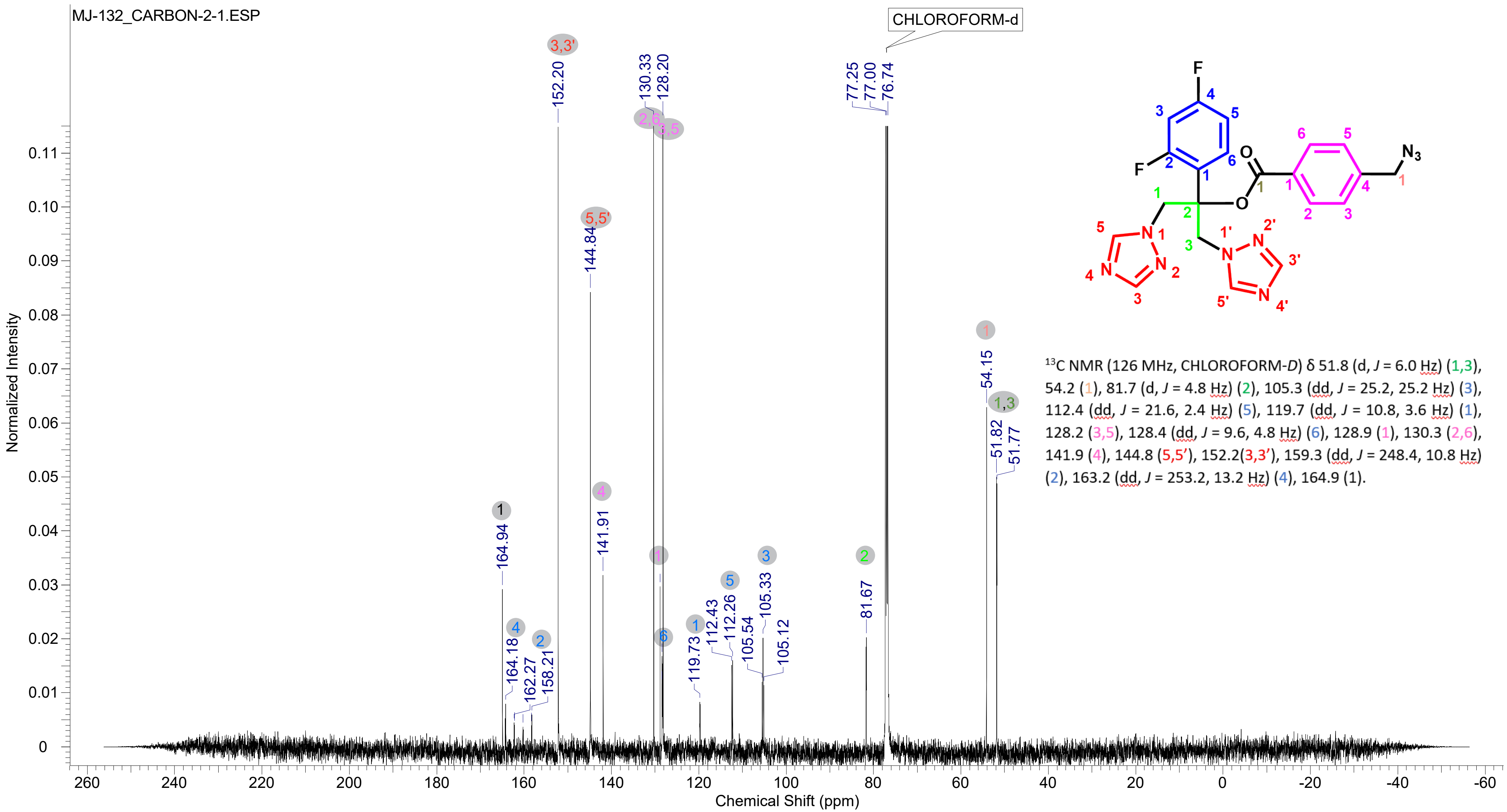

Figure S10. 13C NMR (125 Mhz, CDCl3)

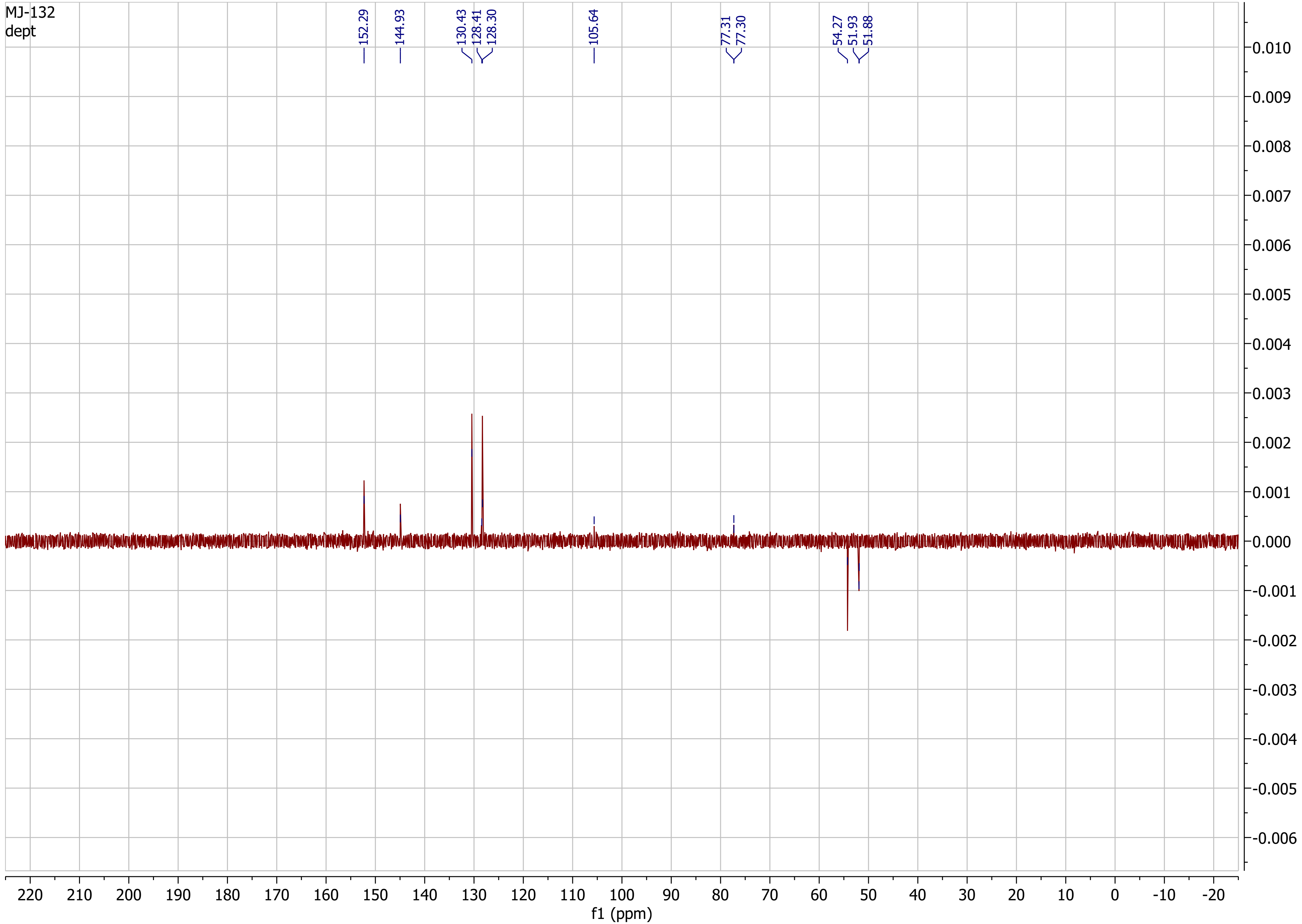

Figure S11. DEPT-135 (125 MHz, CDCl3)

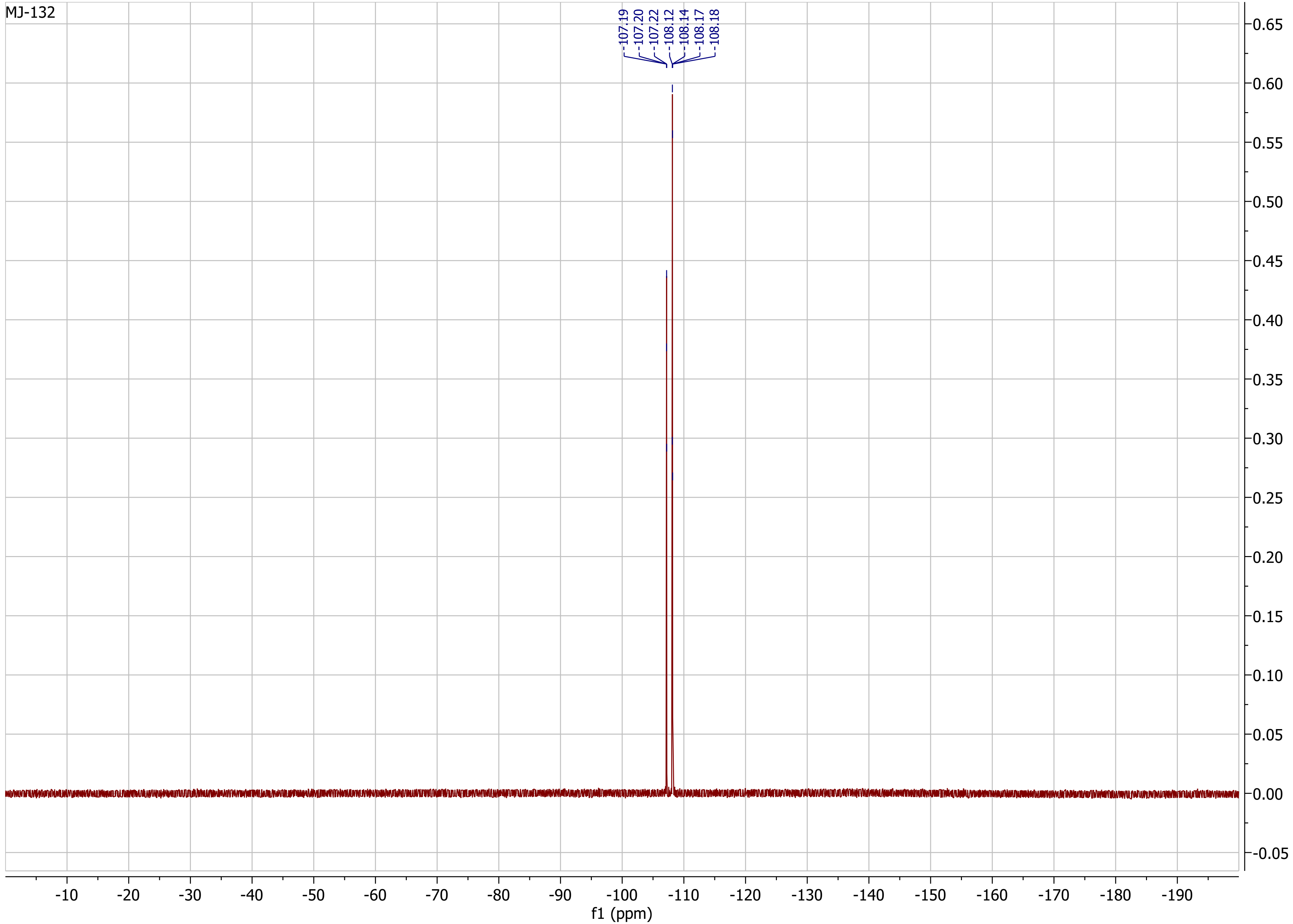

Figure S12. ^{19}F NMR (471 MHz, $CDCl_3$)

S3. 3a

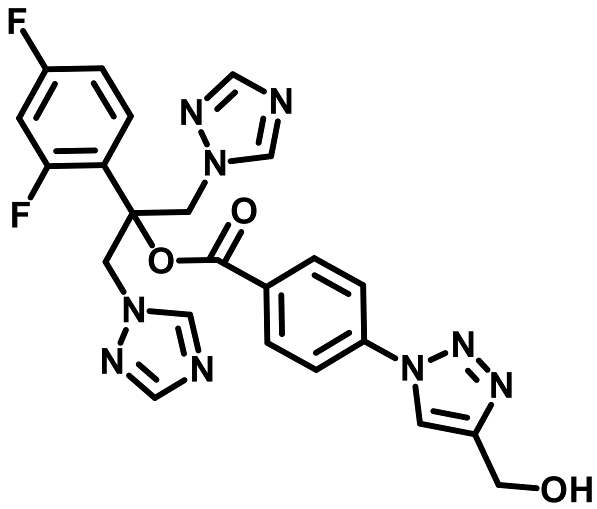

2-(2,4-difluorophenyl)-1,3-di(1H-1,2,4-triazol-1-yl)propan-2-yl 4-(4-(hydroxymethyl)-1H-1,2,3-triazol-1-yl)benzoate

Figure S13: LC-MS (ESI)

Figure S14: HRMS (ESI), calc/found m/z, Δ ppm

Figure S15: ^{1}H NMR (500 MHz, $CDCl_3$)

Figure S16: ^{13}C NMR (125 MHz, $CDCl_3$)

Figure S17: DEPT-135 (125 MHz, $CDCl_3$)

SHIMADZU LabSolutions

Analysis Report

Sample Name	: MJ43		
Sample ID	:		
Data Filename	: MJ43_MeOH_70-15m-03_(150-1500da)_18-02-2026_4.lcd		
Method Filename	: MeOH_70-15m-03_(150-1500da).lcm		
Batch Filename	: 18-02-2026.lcb		
Vial #	: 3-8	Sample Type	: Unknown
Injection Volume	: 0.1 uL		
Date Acquired	: 2/18/2026 2:08:53 PM	Acquired by	: System Administrator
Date Processed	: 2/18/2026 2:23:53 PM	Processed by	: System Administrator

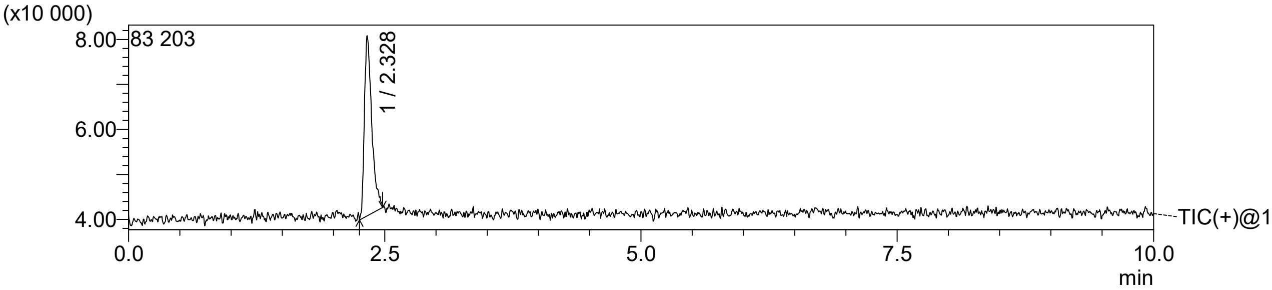

MASS Peak Table TIC

Peak#	Ret. Time	m/z	Area%
1	2.328	TIC	100.000
Total			100.000

MS Spectrum

Line#:1 R.Time:----(Scan#:----)
MassPeaks:39
Spectrum Mode:Averaged 2.320-2.330(465-467) Base Peak:508(26537)
BG Mode:Calc Segment 1 - Event 1

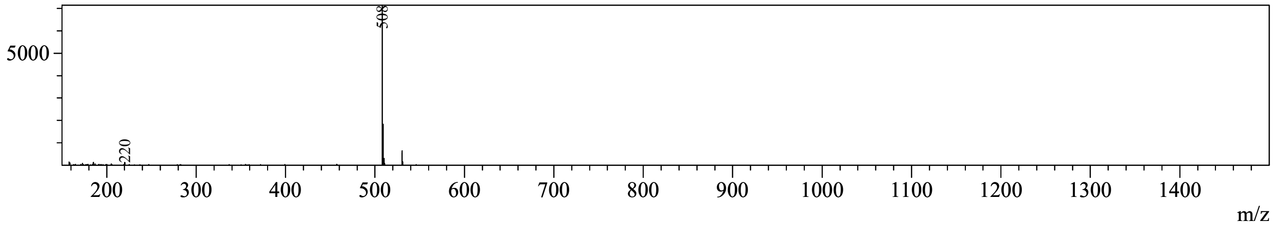

Figure S13. LC-MS (ESI)

Formula Predictor Report

Printed at 19.02.2026 14:13:58

Formula Predictor Result	**C23 H19 N9 O3 F2**
Mass	508.16532
Error Margin	60 ppm
DBE Range	Not Used
Electron Ions	Both configurations
HC Ratio	Not Used
Nitrogen Rule	Used

#	Score	Pred. (M)	Pred. m/z	Meas. m/z	Diff. (mDa)	Formulae (M)	Ion	Diff. (ppm)	Iso Score	DBE
1	91.64	507.15789	508.16517	508.16532	0.15	C23 H19 N9 O3 F2	[M+H]+	0.299	90.72	18.0

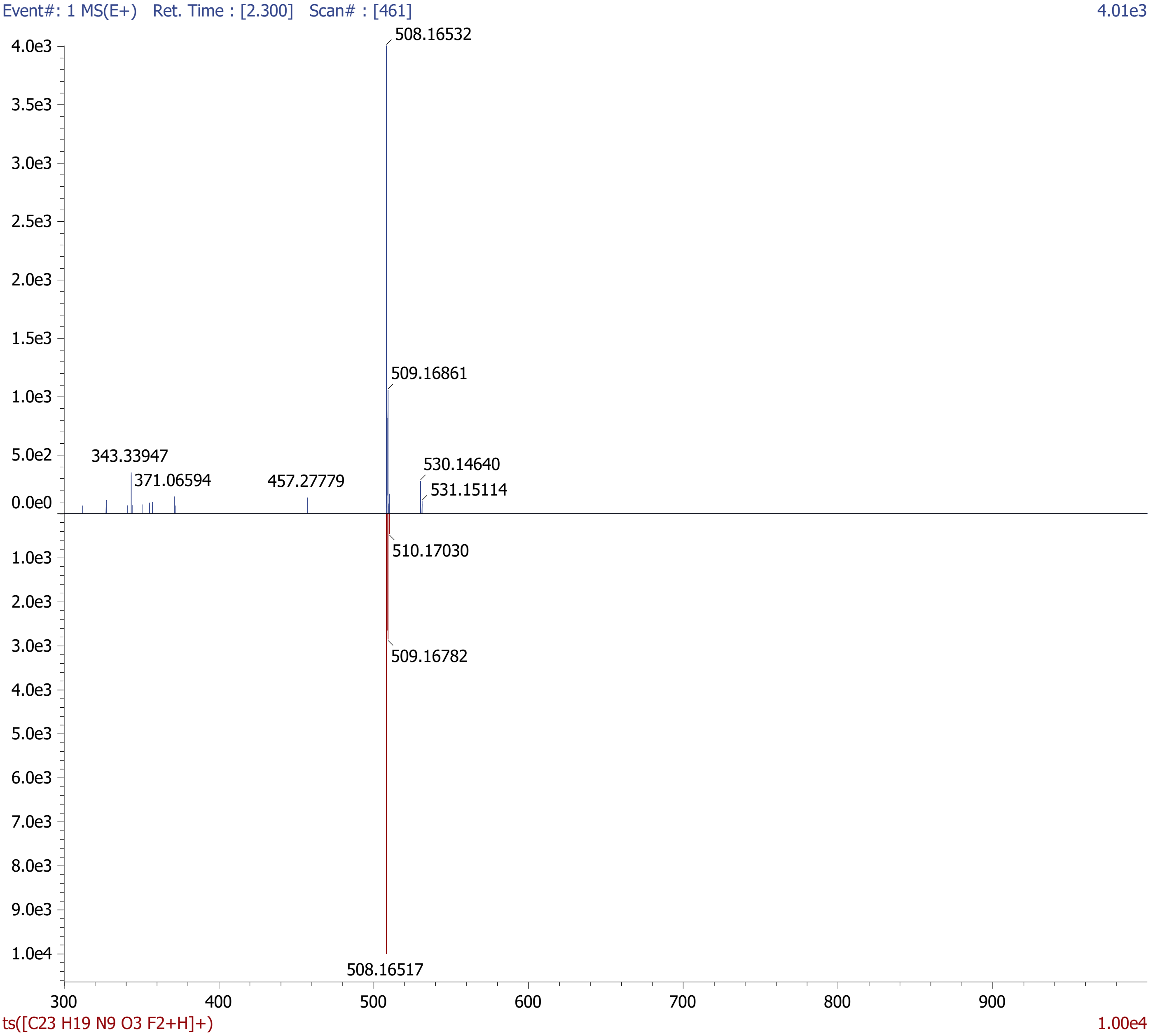

Figure S14. HRMS (ESI), calc/found m/z, Δ ppm

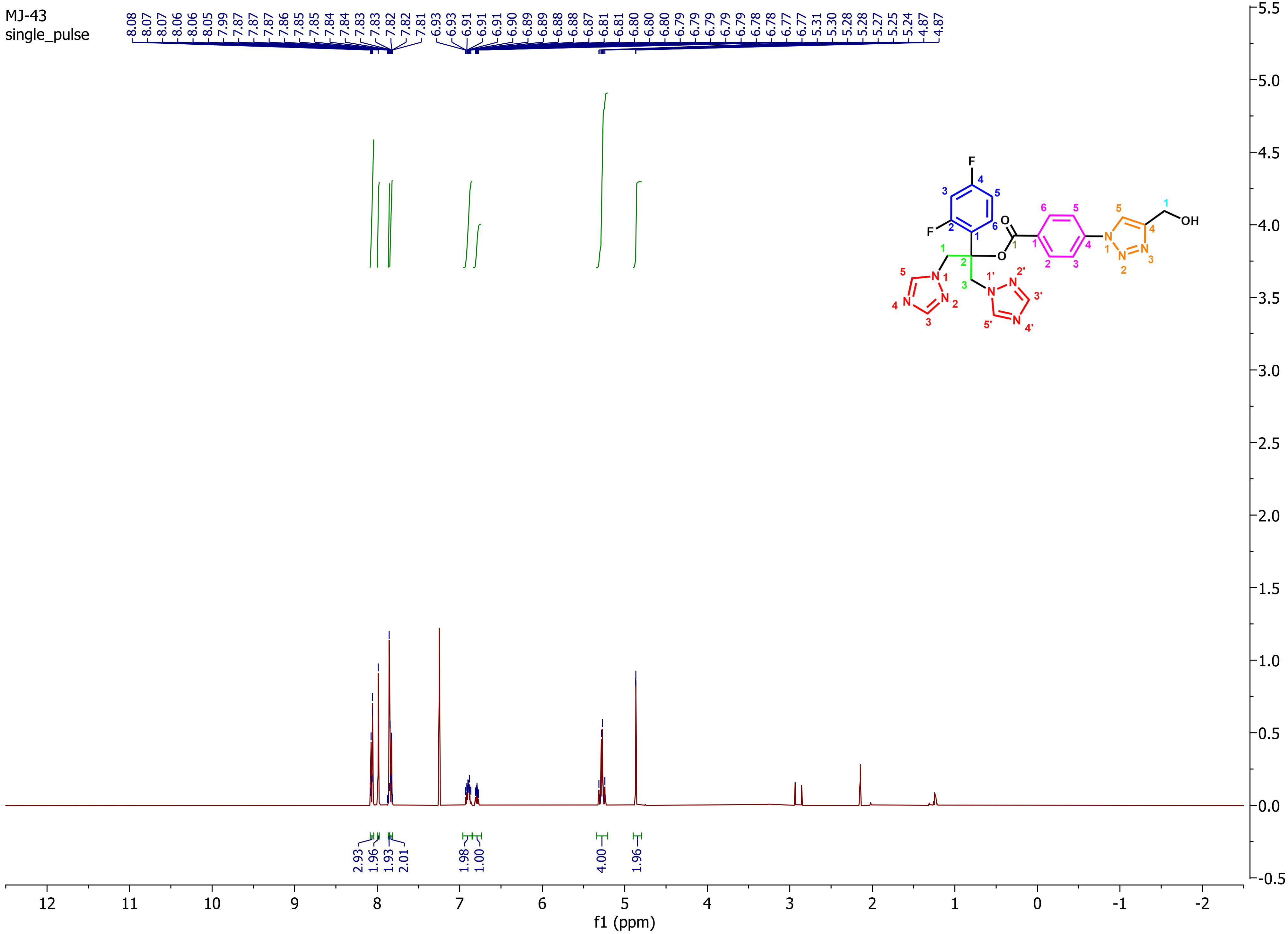

Figure S15. ^{1}HNMR (500 MHz, $CDCl_3$)

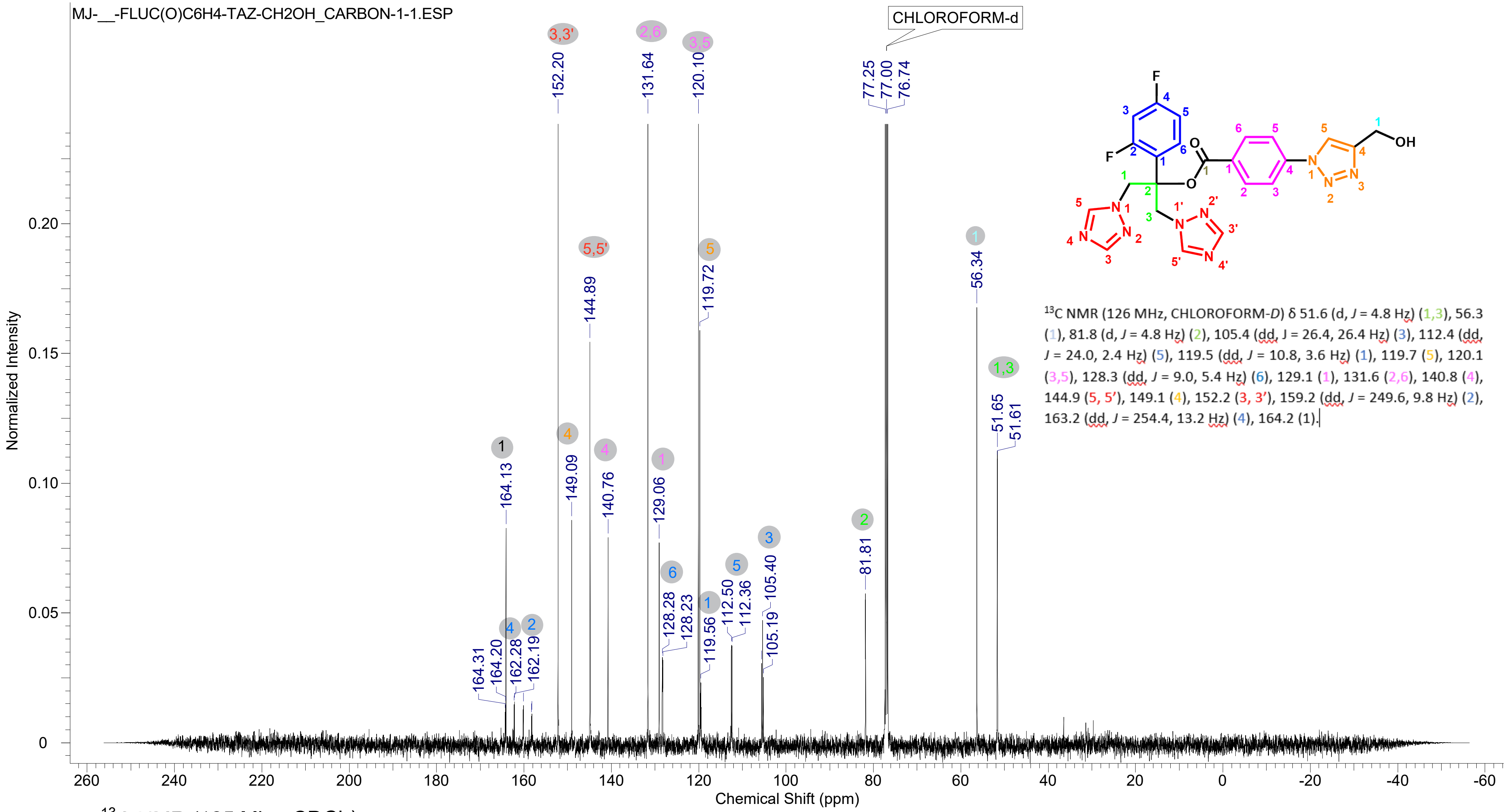

Figure S16. ^{13}C NMR (125 Mhz, $CDCl_3$)

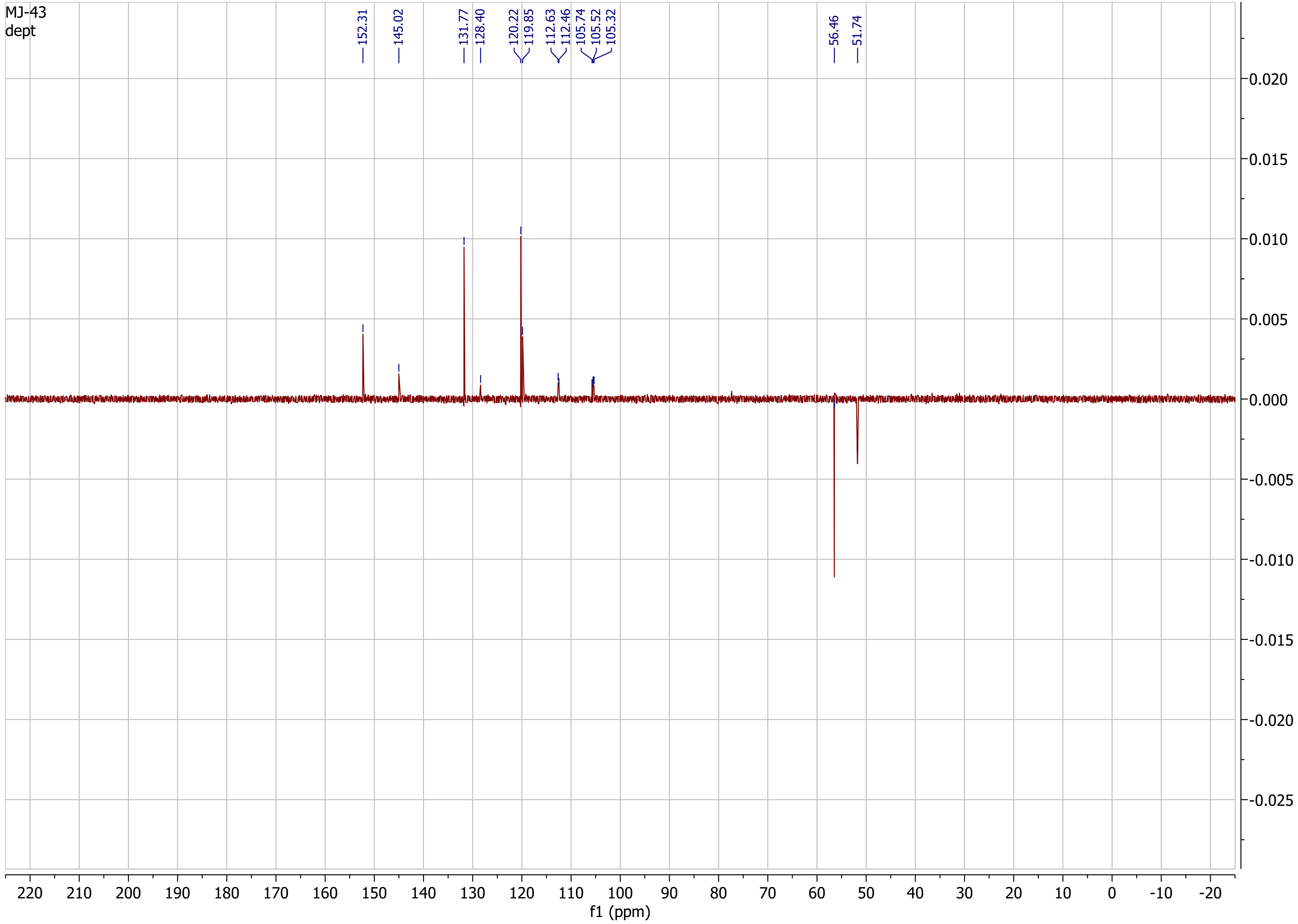

Figure S17. DEPT-135 (125 MHz, $CDCl_3$)

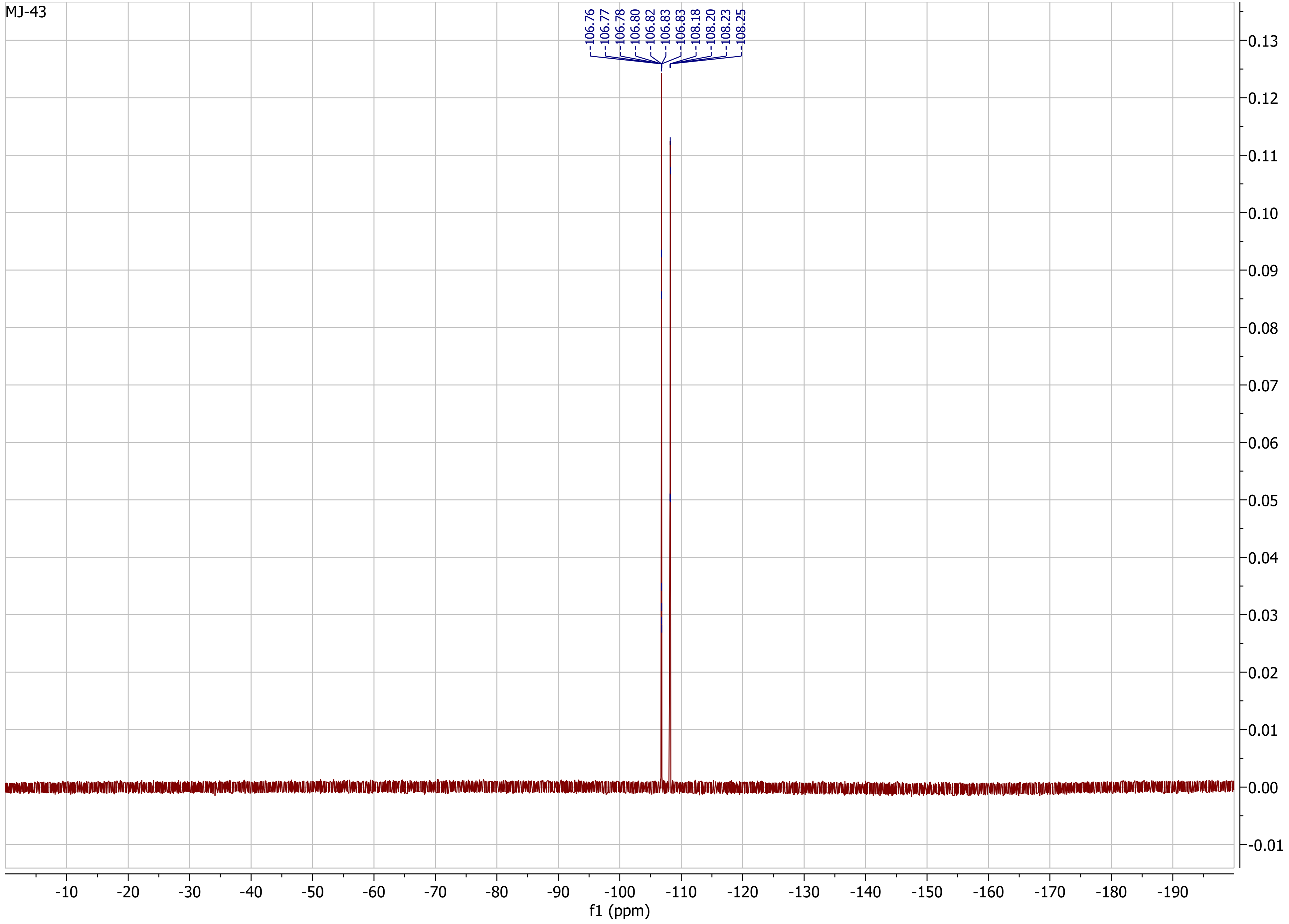

Figure S18. ^{19}F NMR (471 MHz, $CDCl_3$)

S4. 3b

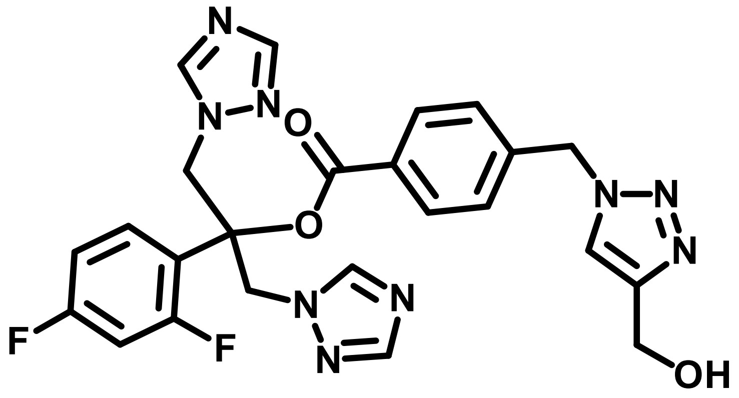

2-(2,4-difluorophenyl)-1,3-di(1H-1,2,4-triazol-1-yl)propan-2-yl 4-((4-(hydroxymethyl)-1H-1,2,3-triazol-1-yl)methyl)benzoate

Figure S19: LC-MS (ESI)

Figure S20: HRMS (ESI), calc/found m/z, Δ ppm

Figure S21: ^{1}H NMR (500 MHz, $CDCl_3$)

Figure S22: ^{13}C NMR (125 MHz, $CDCl_3$)

Figure S23: DEPT-135 (125 MHz, $CDCl_3$)

Figure S24: ^{19}F NMR (471 MHz, $CDCl_3$)

SHIMADZU LabSolutions

Analysis Report

Sample Name	: MJ-137		
Sample ID	:		
Data Filename	: MJ-137_MeOH_70-30m-03_(110-1500da)_2-12-2025_8.lcd		
Method Filename	: MeOH_70-30m-03_(110-1500da).lcm		
Batch Filename	: 2-12-2025.lcb		
Vial #	: 3-51	Sample Type	: Unknown
Injection Volume	: 0.2 uL		
Date Acquired	: 12/2/2025 5:12:15 PM	Acquired by	: System Administrator
Date Processed	: 12/2/2025 5:31:31 PM	Processed by	: System Administrator

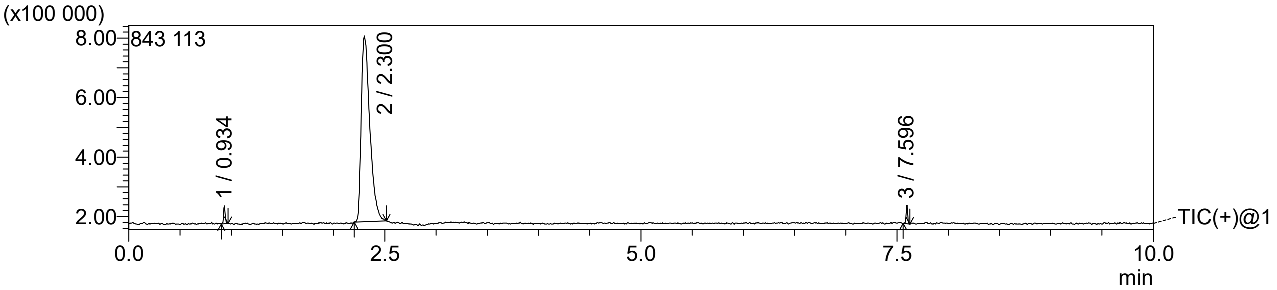

MASS Peak Table TIC

Peak#	Ret. Time	m/z	Area%
1	0.934	TIC	1.774
2	2.300	TIC	96.558
3	7.596	TIC	1.668
Total			100.000

MS Spectrum

Line#:1 R.Time:----(Scan#:----)
MassPeaks:177
Spectrum Mode:Averaged 0.925-0.935(186-188) Base Peak:388(11686)
BG Mode:Calc Segment 1 - Event 1

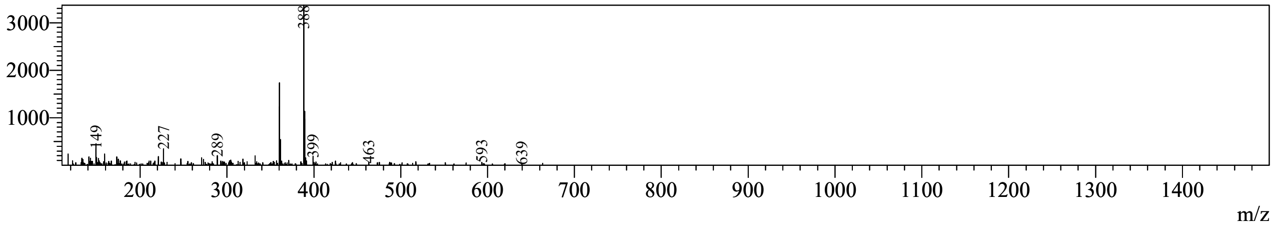

Line#:2 R.Time:----(Scan#:----)
MassPeaks:200
Spectrum Mode:Averaged 2.295-2.305(460-462) Base Peak:522(283235)
BG Mode:Calc Segment 1 - Event 1

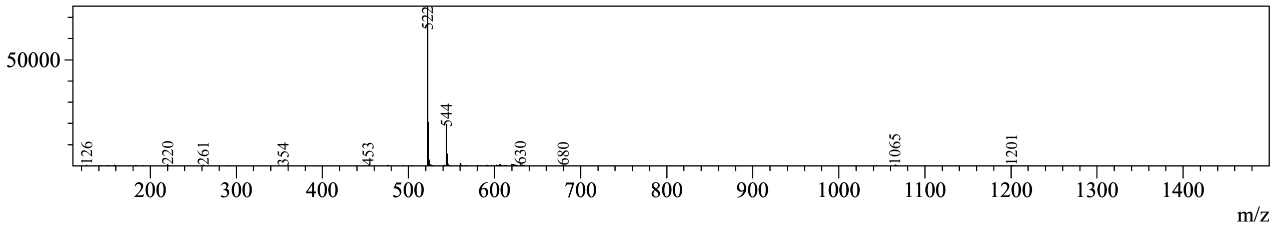

Line#:3 R.Time:----(Scan#:----)
MassPeaks:191
Spectrum Mode:Averaged 7.590-7.600(1519-1521) Base Peak:388(13247)
BG Mode:Calc Segment 1 - Event 1

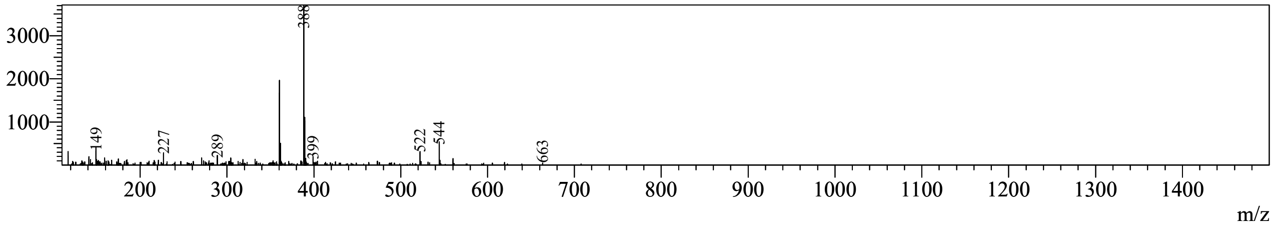

Figure S19. LC-MS (ESI)

Formula Predictor Report

Printed at 23.04.2026 14:32:51

Formula Predictor Result	**C24 H21 N9 O3 F2**
Mass	522.18145
Error Margin	30 ppm
DBE Range	Not Used
Electron Ions	Both configurations
HC Ratio	Not Used
Nitrogen Rule	Used

#	Score	Pred. (M)	Pred. m/z	Meas. m/z	Diff. (mDa)	Formulae (M)	Ion	Diff. (ppm)	Iso Score	DBE
5	74.95	521.17354	522.18082	522.18145	0.63	C24 H21 N9 O3 F2	[M+H]+	1.210	72.16	18.0

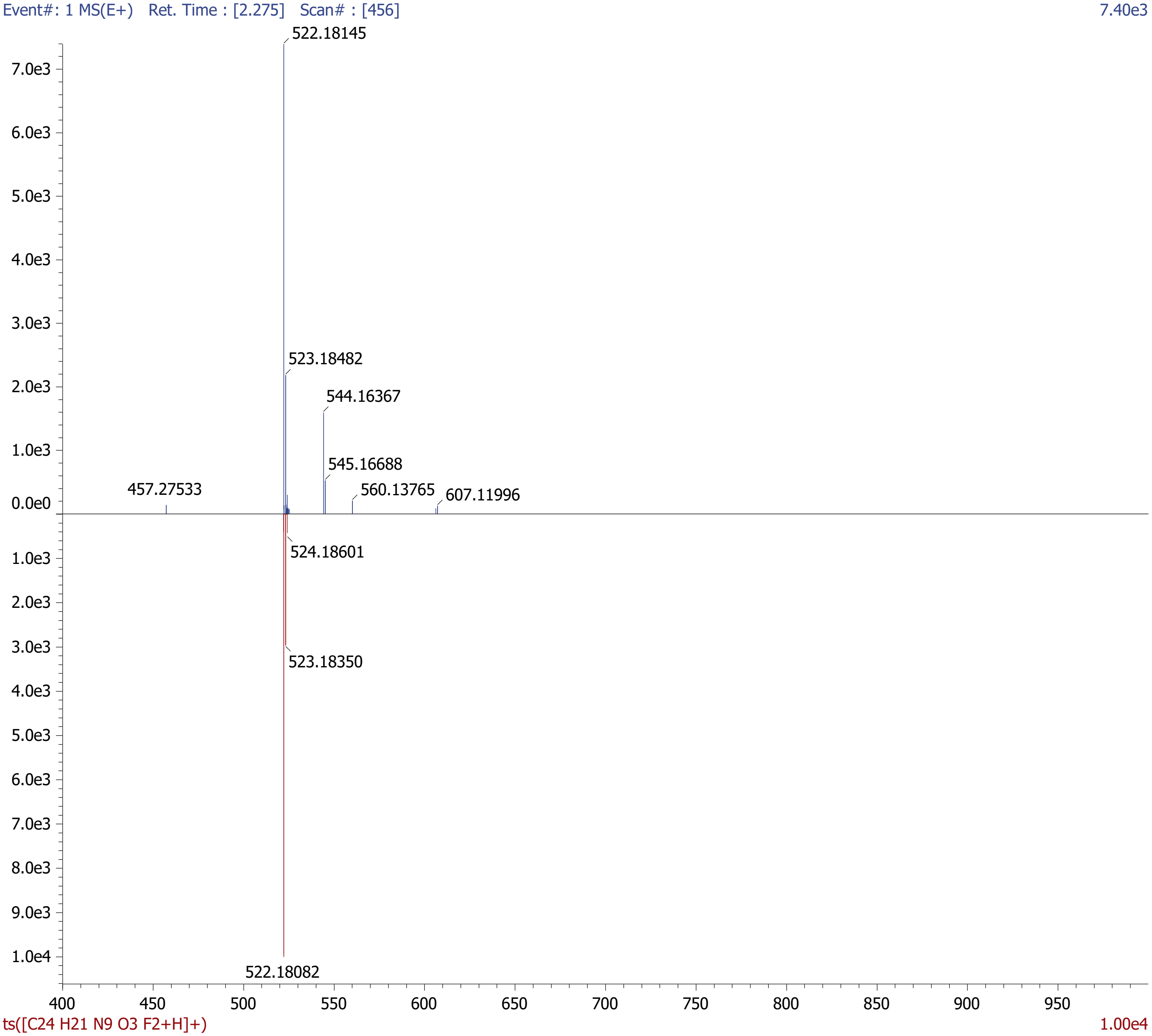

Figure S20. HRMS (ESI), calc/found m/z, Δ ppm

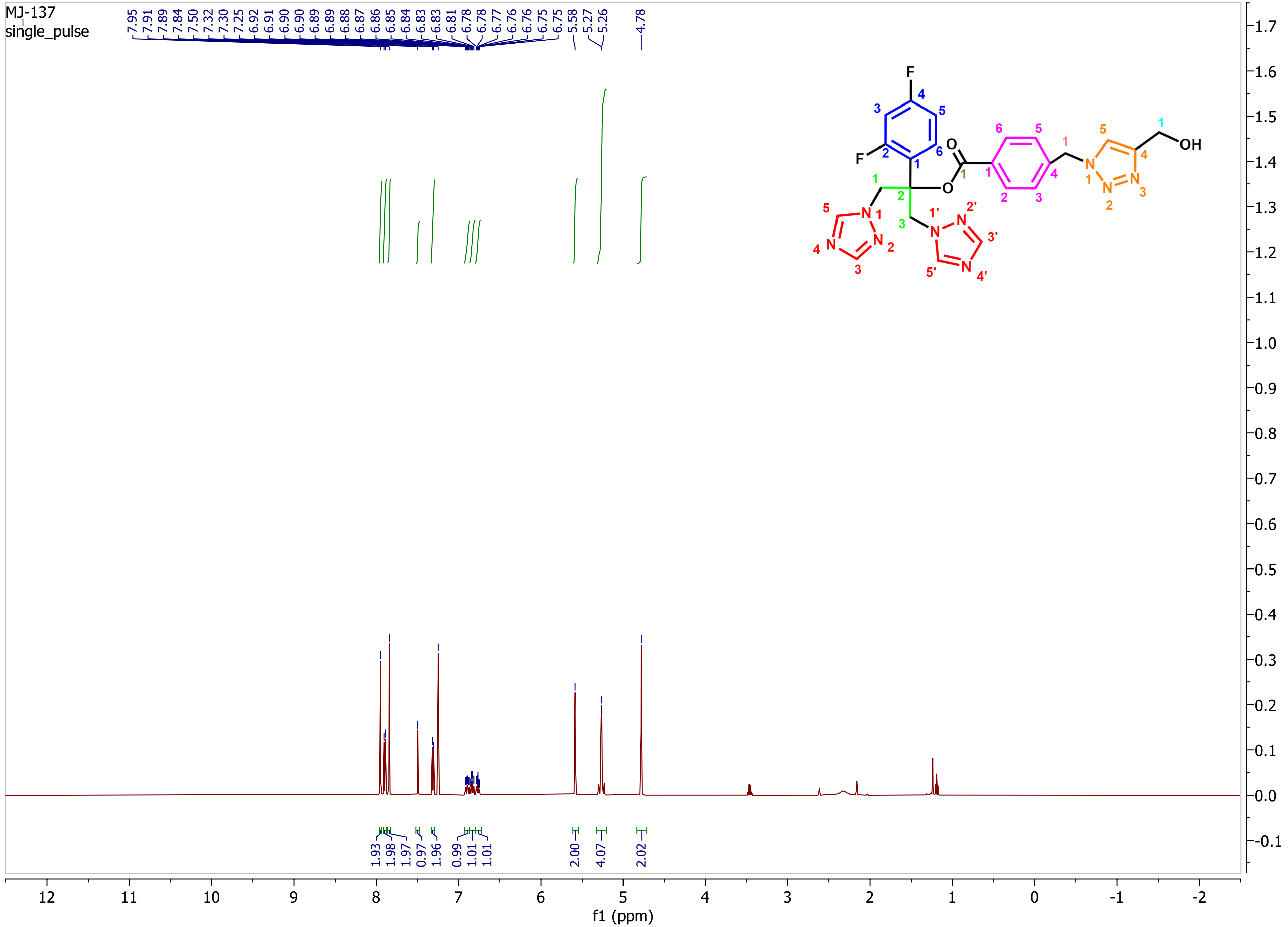

Figure S21. ^{1}HNMR (500 MHz, $CDCl_3$)

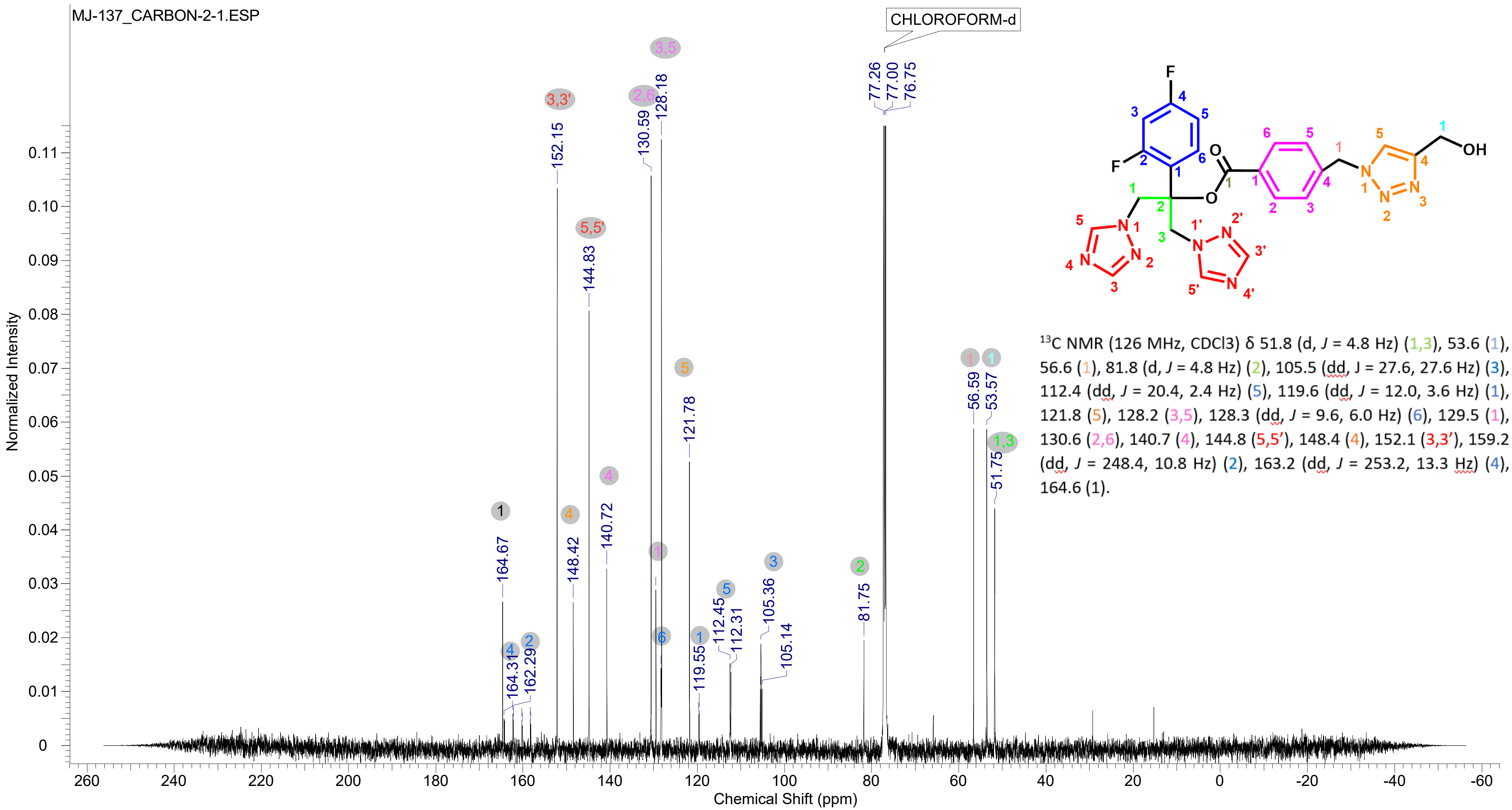

^{13}C NMR (126 MHz, CDCl3) δ 51.8 (d, *J* = 4.8 Hz) (1,3), 53.6 (1), 56.6 (1), 81.8 (d, *J* = 4.8 Hz) (2), 105.5 (dd, J = 27.6, 27.6 Hz) (3), 112.4 (dd, *J* = 20.4, 2.4 Hz) (5), 119.6 (dd, *J* = 12.0, 3.6 Hz) (1), 121.8 (5), 128.2 (3,5), 128.3 (dd, *J* = 9.6, 6.0 Hz) (6), 129.5 (1), 130.6 (2,6), 140.7 (4), 144.8 (5,5′), 148.4 (4), 152.1 (3,3′), 159.2 (dd, *J* = 248.4, 10.8 Hz) (2), 163.2 (dd, *J* = 253.2, 13.3 Hz) (4), 164.6 (1).

Figure S22. ^{13}C NMR (125 Mhz, $CDCl_3$)

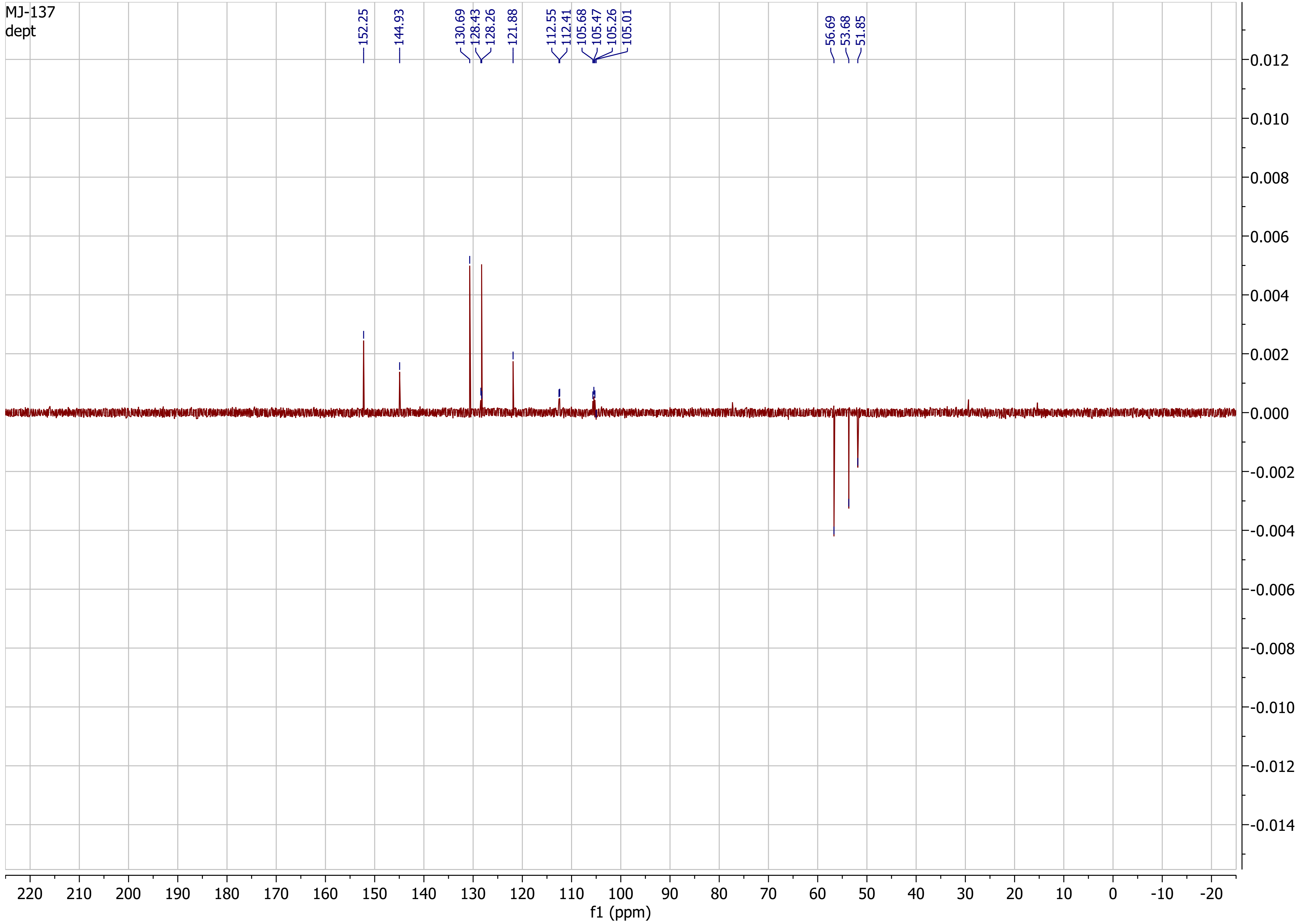

Figure S23. DEPT-135 (125 MHz, $CDCl_3$)

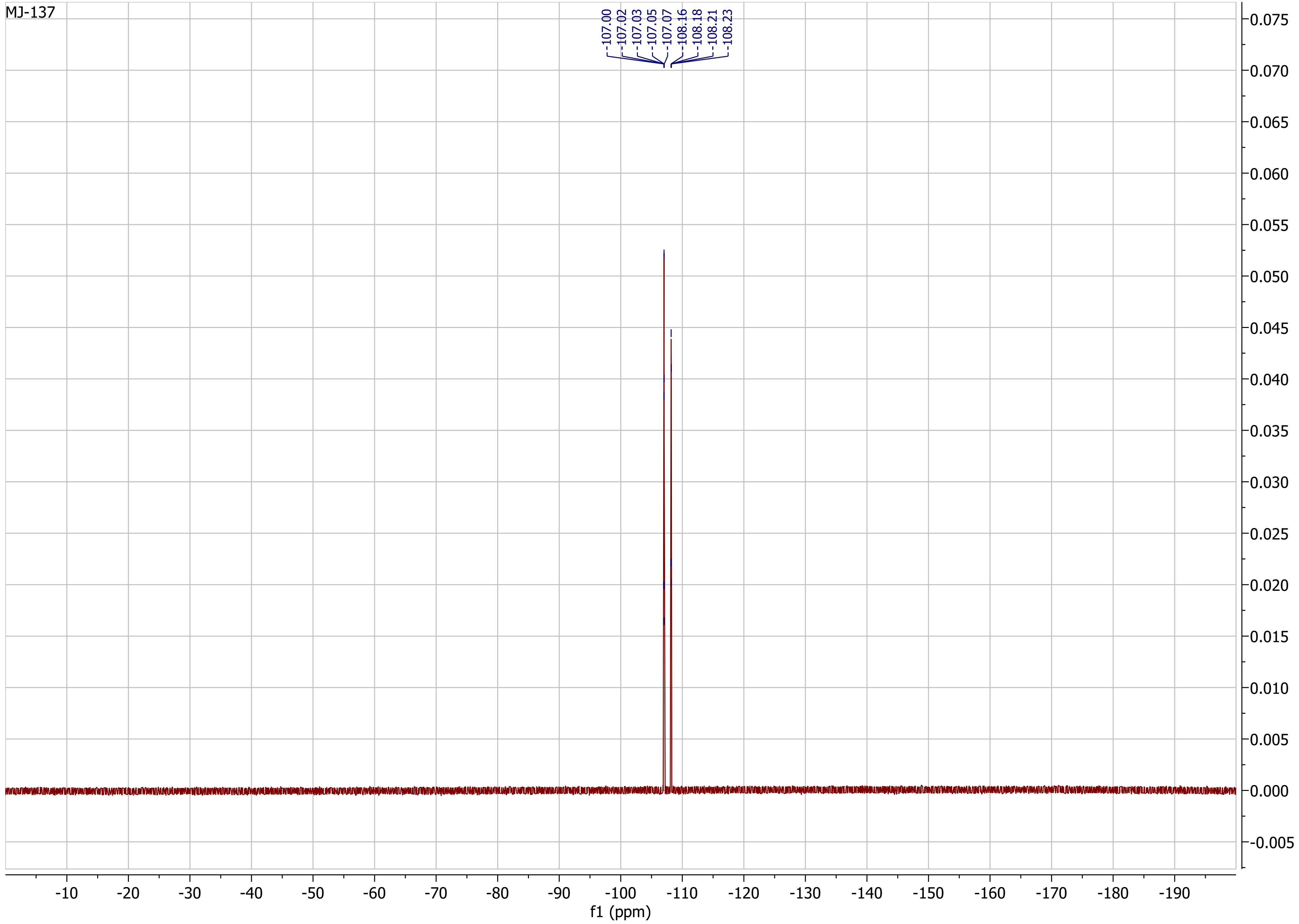

Figure S24. ^{19}F NMR (471 MHz, $CDCl_3$)

S5. 3c

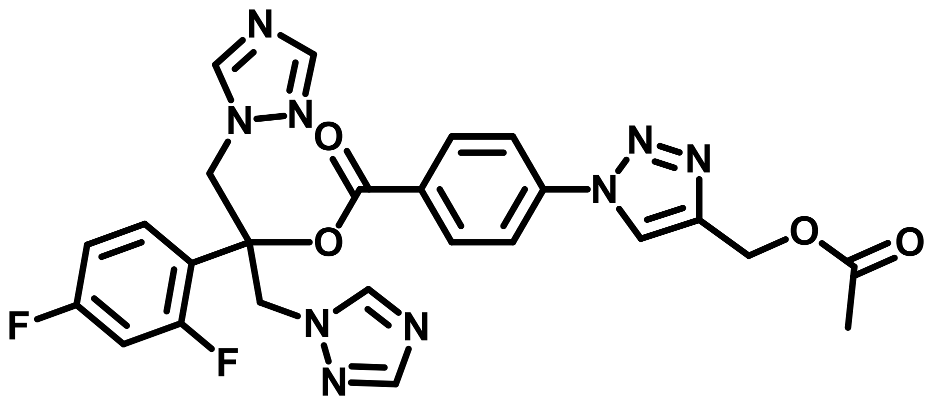

2-(2,4-difluorophenyl)-1,3-di(1H-1,2,4-triazol-1-yl)propan-2-yl 4-(4-(acetoxymethyl)-1H-1,2,3-triazol-1-yl)benzoate

Figure S25: LC-MS (ESI)

Figure S26: HRMS (ESI), calc/found m/z, Δ ppm

Figure S27: ^{1}H NMR (500 MHz, $CDCl_3$)

Figure S28: ^{13}C NMR (125 MHz, $CDCl_3$)

Figure S29: DEPT-135 (125 MHz, $CDCl_3$)

Figure S30: ^{19}F NMR (471 MHz, $CDCl_3$)

SHIMADZU LabSolutions

Analysis Report

Sample Name	: MJ-156		
Sample ID	:		
Data Filename	: MJ-156_MeOH_70-30m-03_(110-1500da)_13-01-2026_7.lcd		
Method Filename	: MeOH_70-30m-03_(110-1500da).lcm		
Batch Filename	: 13-01-2026.lcb		
Vial #	: 1-46	Sample Type	: Unknown
Injection Volume	: 0.1 uL		
Date Acquired	: 1/13/2026 4:02:44 PM	Acquired by	: System Administrator
Date Processed	: 1/13/2026 4:32:45 PM	Processed by	: System Administrator

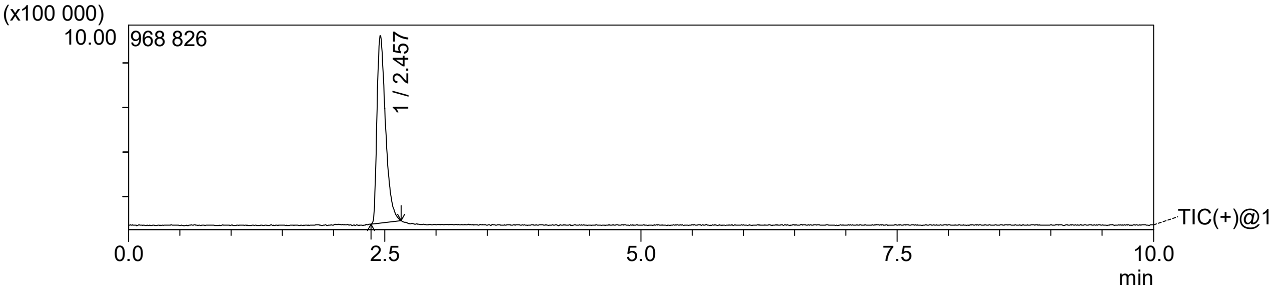

MASS Peak Table TIC

Peak#	Ret. Time	m/z	Area%
1	2.457	TIC	100.000
Total			100.000

MS Spectrum

Line#:1 R.Time:----(Scan#:----)
MassPeaks:174
Spectrum Mode:Averaged 2.450-2.460(491-493) Base Peak:550(403935)
BG Mode:Calc Segment 1 - Event 1

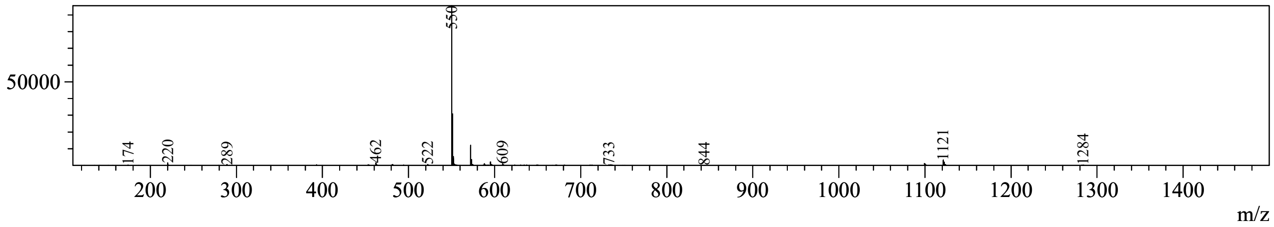

Figure S25. LC-MS (ESI)

Formula Predictor Report

Printed at 23.04.2026 12:20:44

Formula Predictor Result	**C25 H21 N9 O4 F2**
Mass	550.17570
Error Margin	30 ppm
DBE Range	Not Used
Electron Ions	Both configurations
HC Ratio	Not Used
Nitrogen Rule	Used

#	Score	Pred. (M)	Pred. m/z	Meas. m/z	Diff. (mDa)	Formulae (M)	Ion	Diff. (ppm)	Iso Score	DBE
1	99.61	549.16846	550.17573	550.17570	-0.03	C25 H21 N9 O4 F2	[M+H]+	-0.060	99.57	19.0

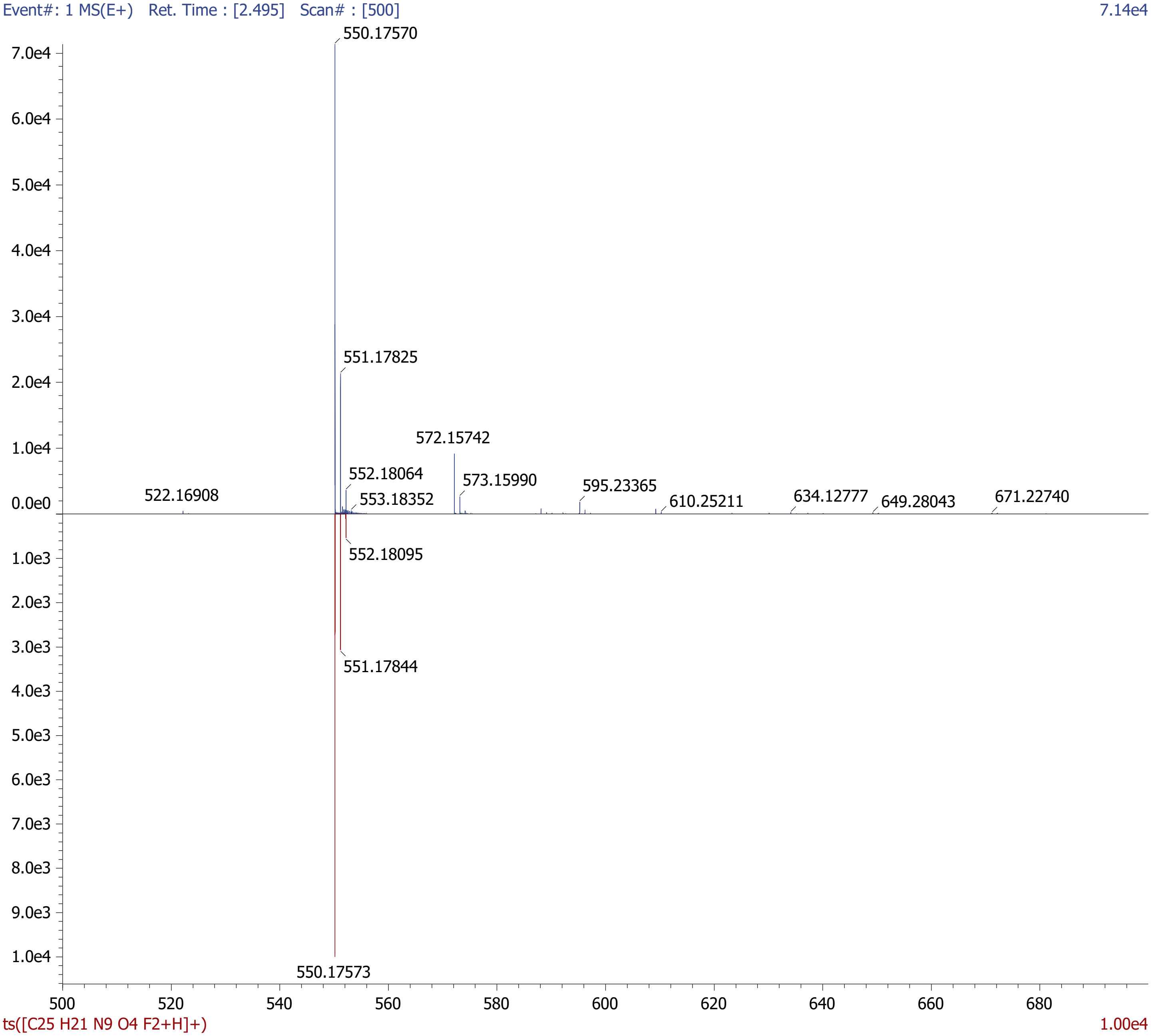

Figure S26. HRMS (ESI), calc/found m/z, Δ ppm

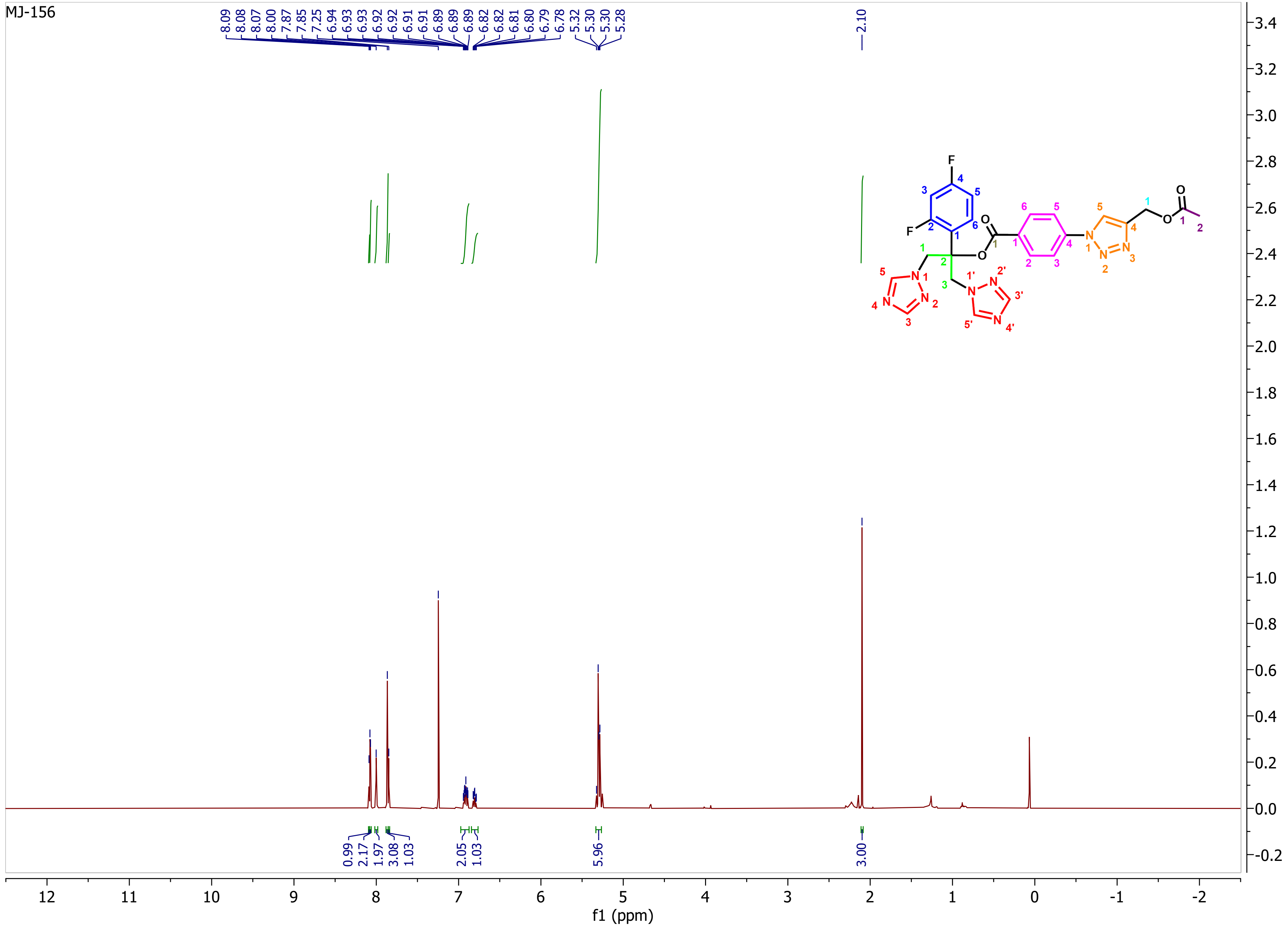

Figure S27. ¹HNMR (500 MHz, $CDCl_3$)

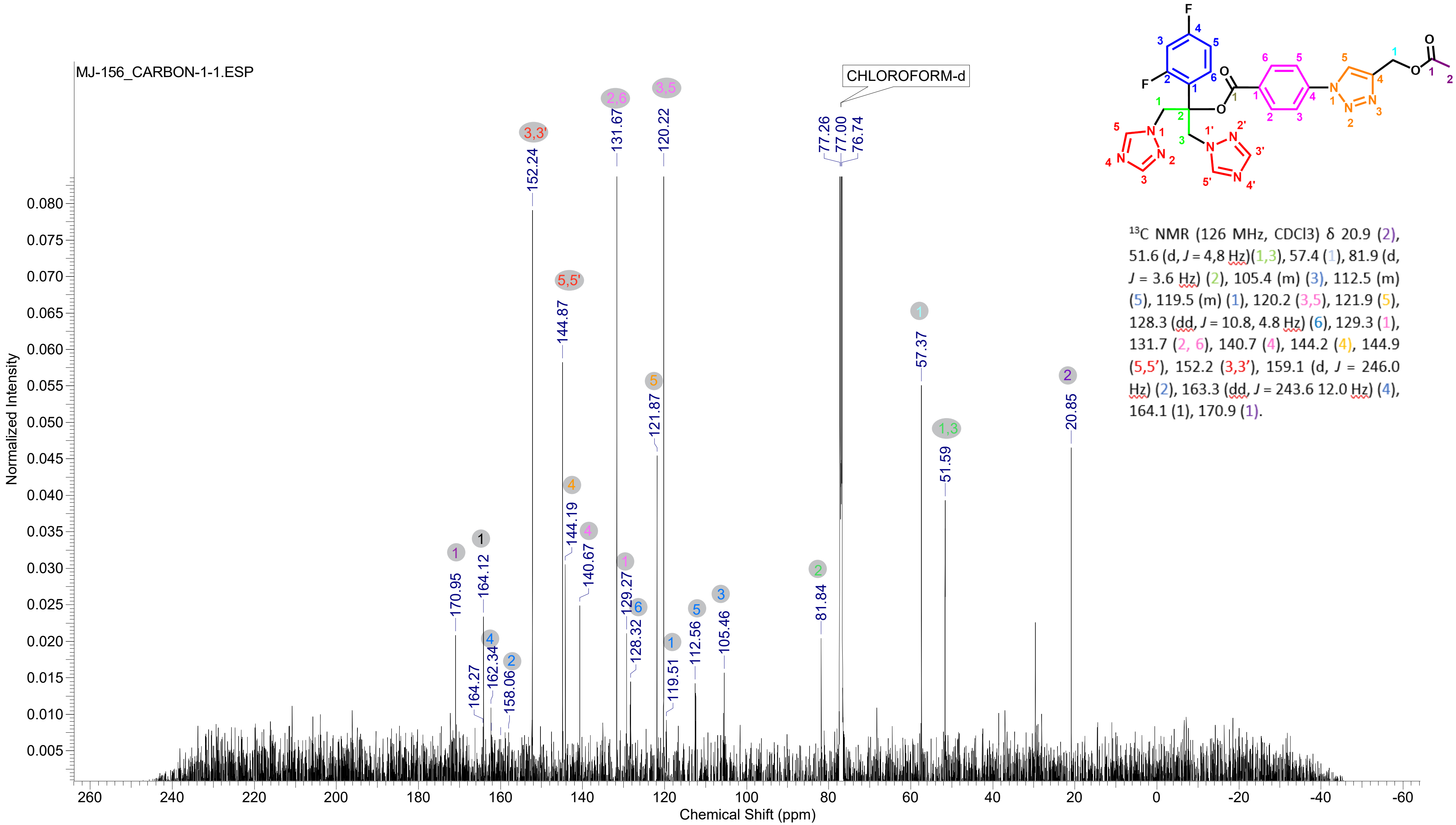

^{13}C NMR (126 MHz, CDCl3) δ 20.9 (2), 51.6 (d, J = 4,8 Hz)(1,3), 57.4 (1), 81.9 (d, J = 3.6 Hz) (2), 105.4 (m) (3), 112.5 (m) (5), 119.5 (m) (1), 120.2 (3,5), 121.9 (5), 128.3 (dd, J = 10.8, 4.8 Hz) (6), 129.3 (1), 131.7 (2, 6), 140.7 (4), 144.2 (4), 144.9 (5,5'), 152.2 (3,3'), 159.1 (d, J = 246.0 Hz) (2), 163.3 (dd, J = 243.6 12.0 Hz) (4), 164.1 (1), 170.9 (1).

Figure S28. 13C NMR (125 Mhz, $CDCl_3$)

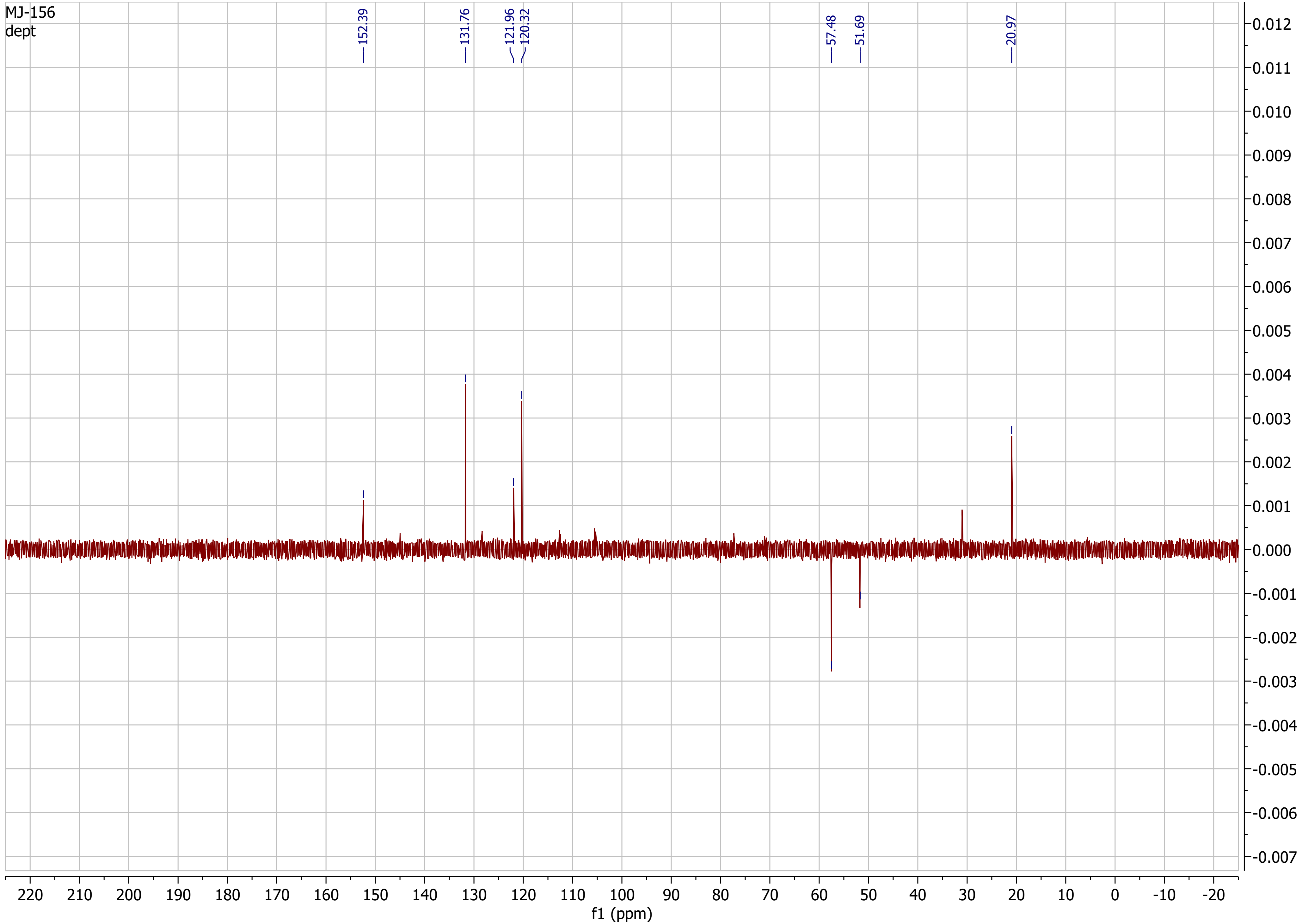

Figure S29. DEPT-135 (125 MHz, $CDCl_3$)

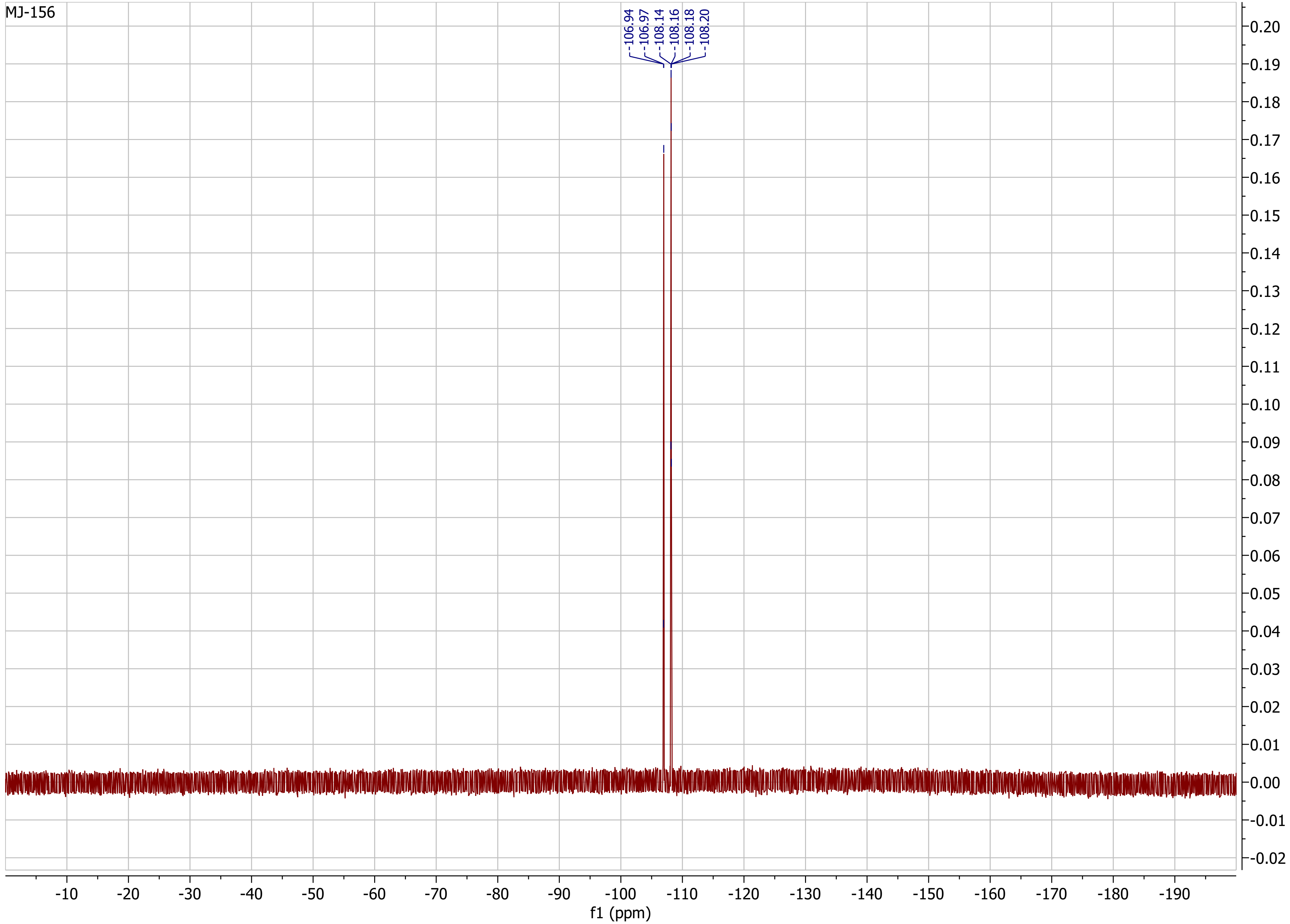

Figure S30. ^{19}F NMR (471 MHz, $CDCl_3$)

S6. 3d

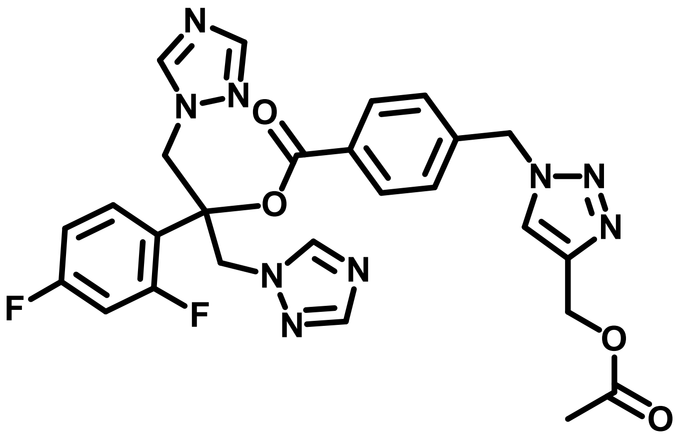

2-(2,4-difluorophenyl)-1,3-di(1H-1,2,4-triazol-1-yl)propan-2-yl 4-((4-(acetoxymethyl)-1H-1,2,3-triazol-1-yl)methyl)benzoate

Figure S31: LC-MS (ESI)

Figure S32: HRMS (ESI), calc/found m/z, Δ ppm

Figure S33: ^{1}H NMR (500 MHz, $CDCl_3$)

Figure S34: ^{13}C NMR (125 MHz, $CDCl_3$)

Figure S35: DEPT-135 (125 MHz, $CDCl_3$)

Figure S36: ^{19}F NMR (471 MHz, $CDCl_3$)

SHIMADZU LabSolutions

Analysis Report

Sample Name : MJ155
Sample ID :
Data Filename : MJ155_MeOH_70-15m-03_(150-1500da)_18-02-2026_9.lcd
Method Filename : MeOH_70-15m-03_(150-1500da).lcm
Batch Filename : 18-02-2026.lcb
Vial # : 3-13
Sample Type : Unknown
Injection Volume : 0.1 uL
Date Acquired : 2/18/2026 3:26:01 PM
Acquired by : System Administrator
Date Processed : 2/18/2026 3:41:01 PM
Processed by : System Administrator

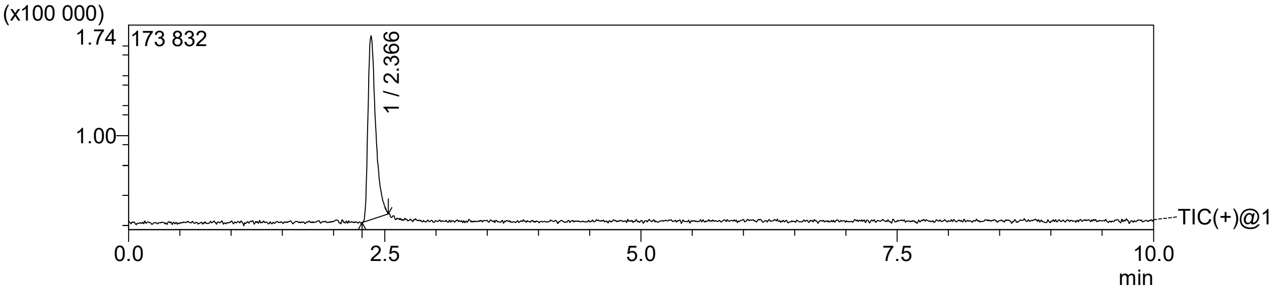

MASS Peak Table TIC

Peak#	Ret. Time	m/z	Area%
1	2.366	TIC	100.000
Total			100.000

MS Spectrum

Line#:1 R.Time:----(Scan#:----)
MassPeaks:68
Spectrum Mode:Averaged 2.360-2.370(473-475) Base Peak:564(48417)
BG Mode:Calc Segment 1 - Event 1

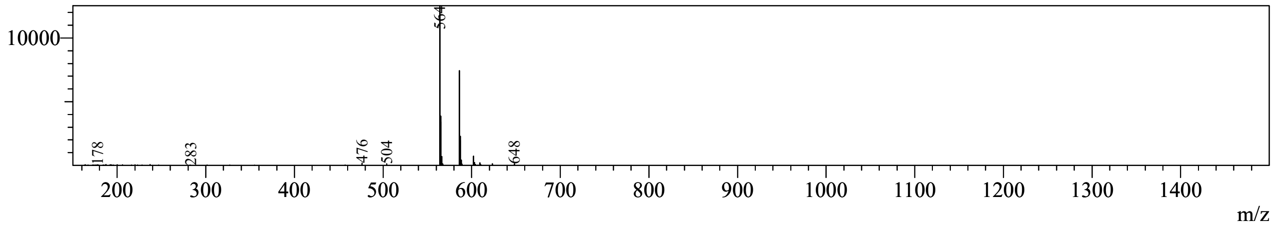

Figure S31. LC-MS (ESI)

Formula Predictor Report

Printed at 23.04.2026 12:18:07

Formula Predictor Result	**C26 H23 N9 O4 F2**
Mass	564.19155
Error Margin	30 ppm
DBE Range	Not Used
Electron Ions	Both configurations
HC Ratio	Not Used
Nitrogen Rule	Used

#	Score	Pred. (M)	Pred. m/z	Meas. m/z	Diff. (mDa)	Formulae (M)	Ion	Diff. (ppm)	Iso Score	DBE
1	98.54	563.18411	564.19138	564.19155	0.17	C26 H23 N9 O4 F2	[M+H]+	0.296	98.38	19.0

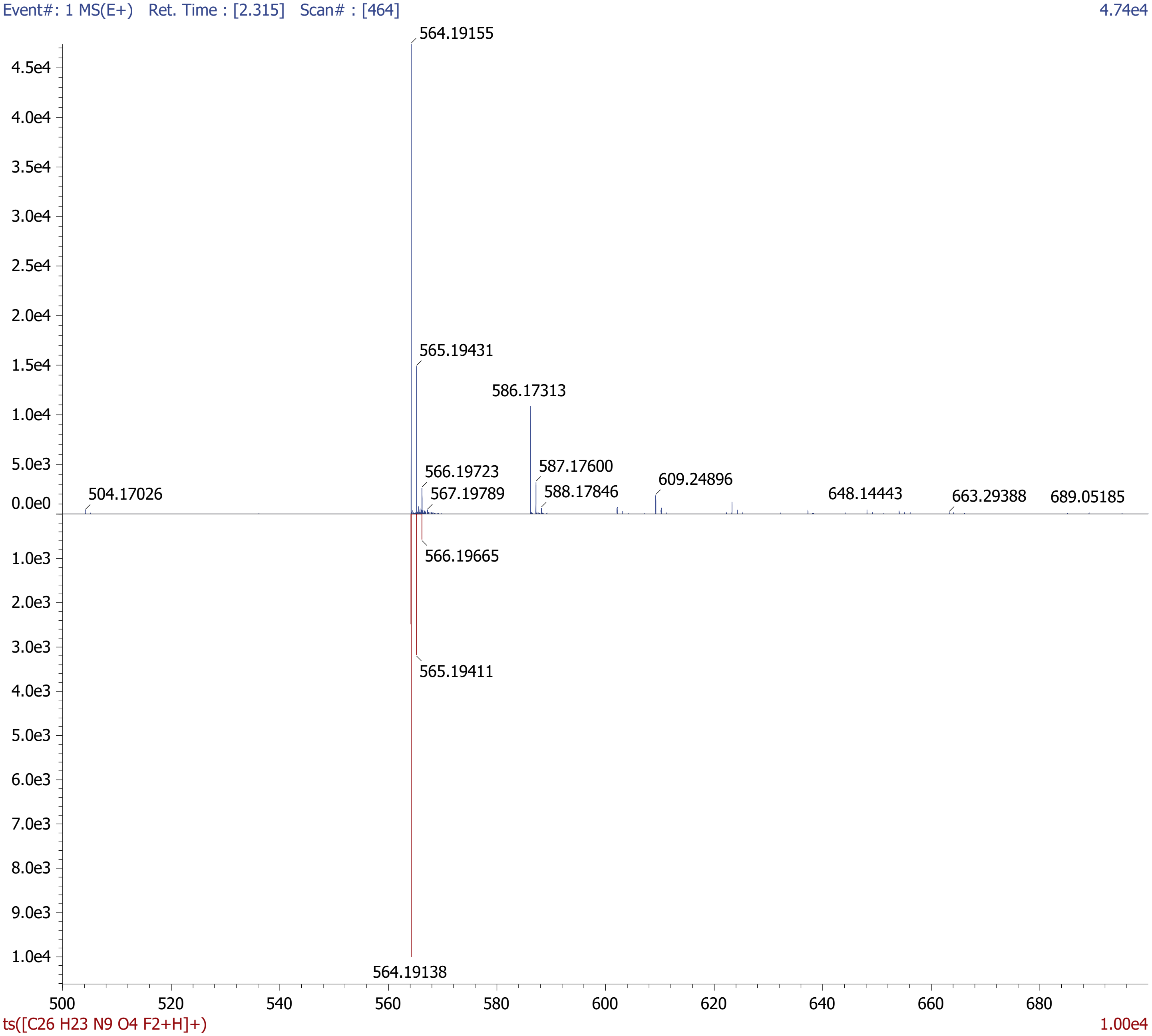

Figure S32. HRMS (ESI), calc/found m/z, Δ ppm

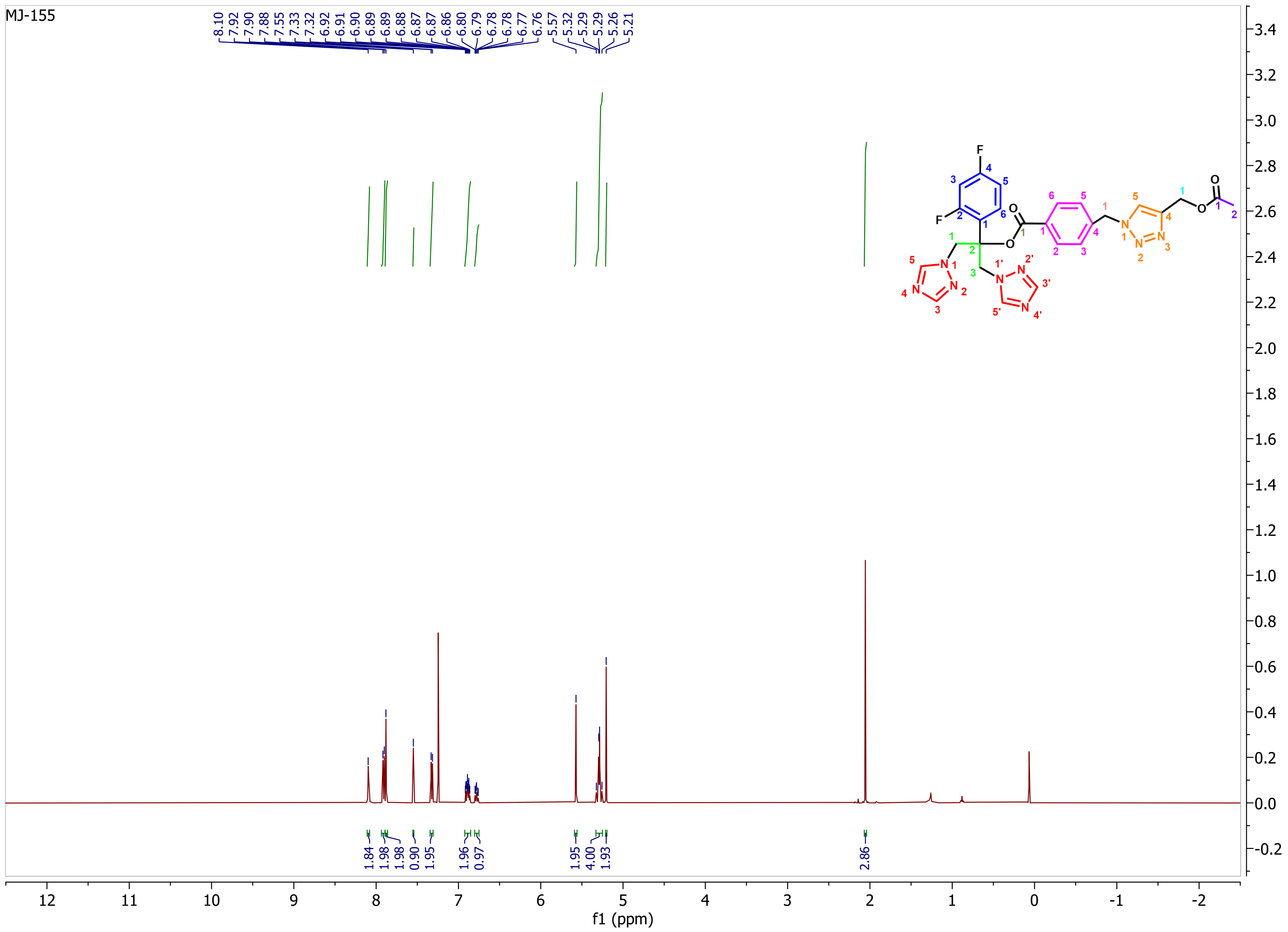

Figure S33. ^{1}HNMR (500 MHz, $CDCl_3$)

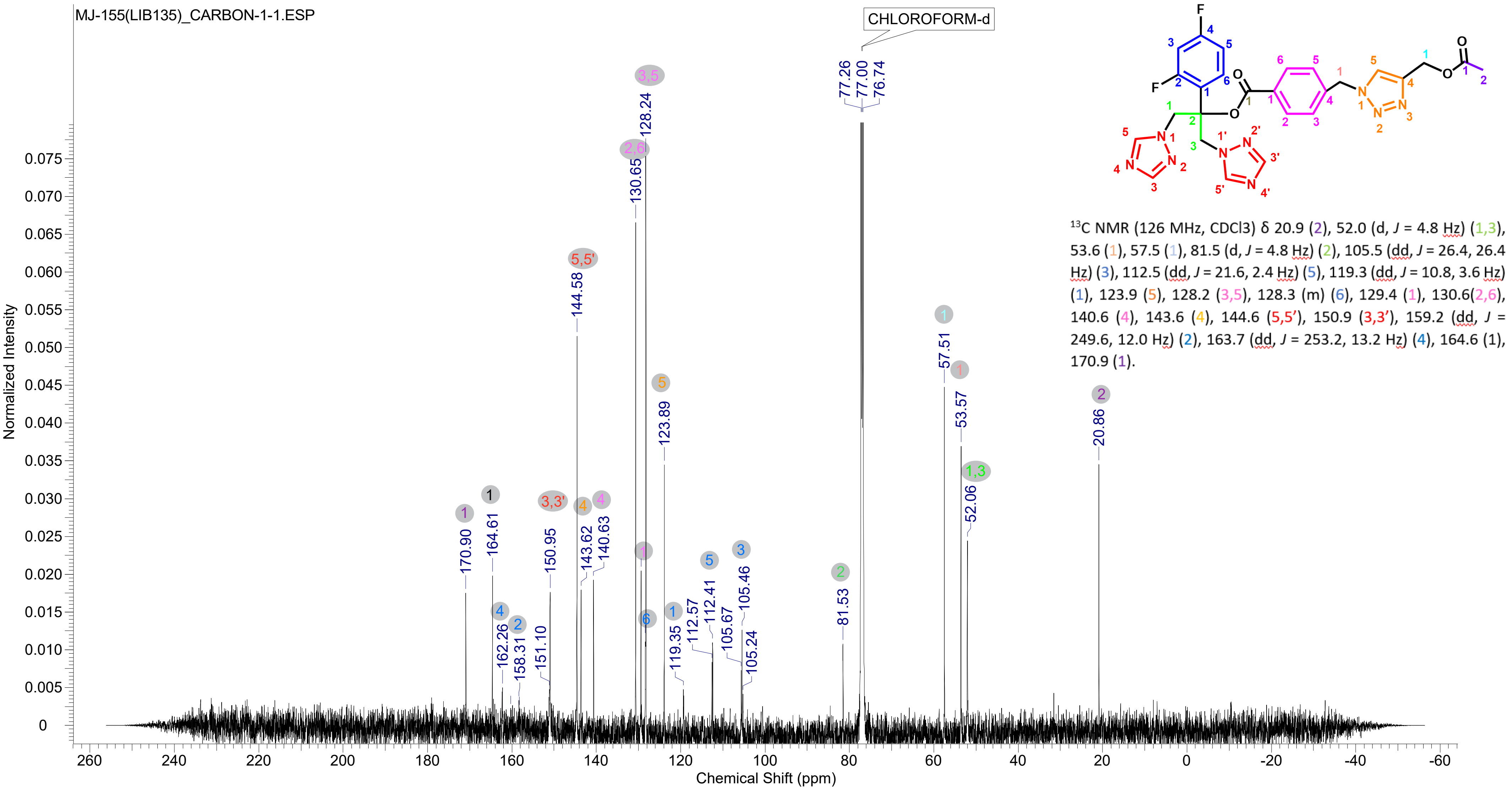

Figure S34. ^{13}C NMR (125 Mhz, $CDCl_3$)

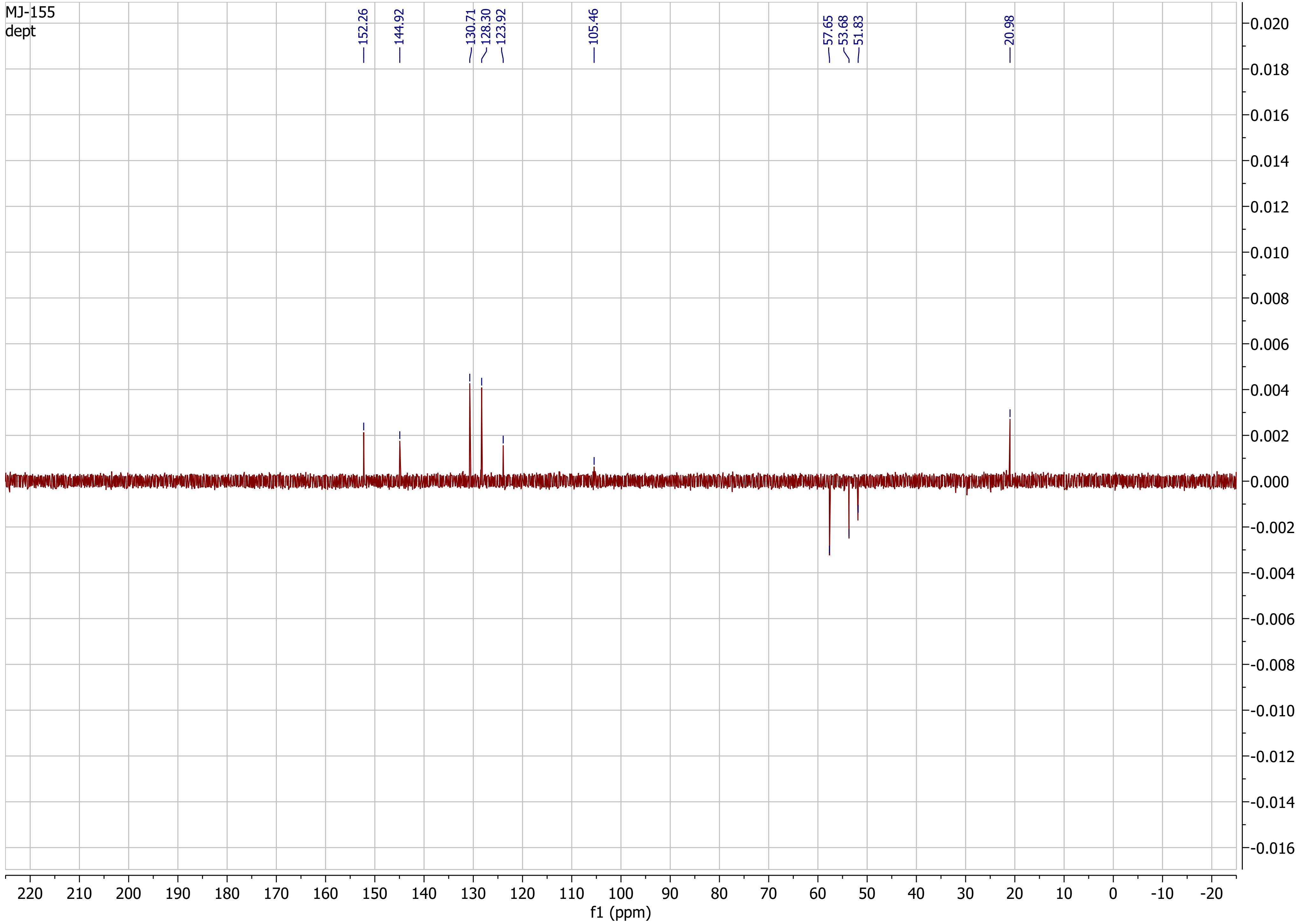

Figure S35. DEPT-135 (125 MHz, $CDCl_3$)

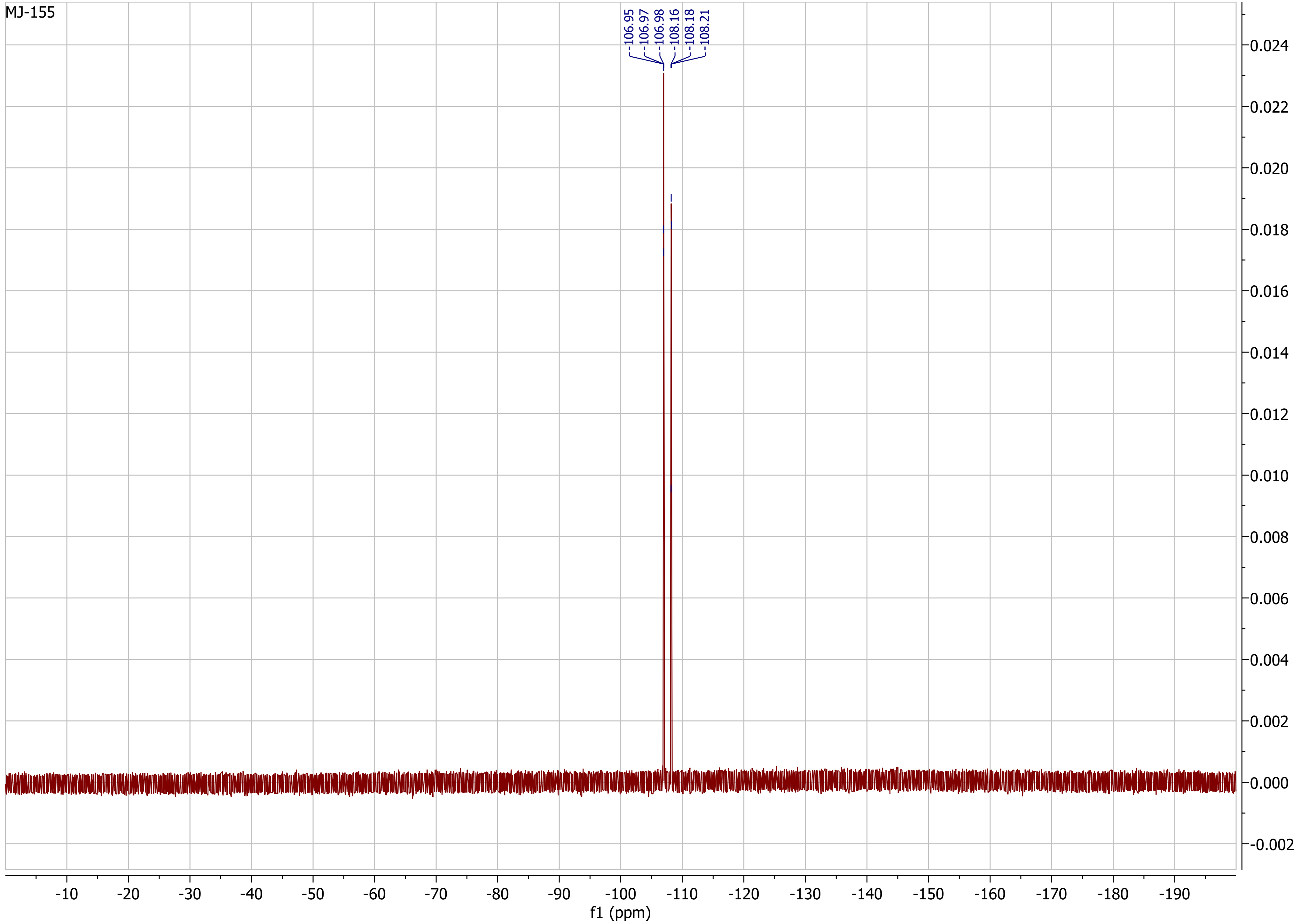

Figure S36. ^{19}F NMR (471 MHz, $CDCl_3$)

S7. 3e

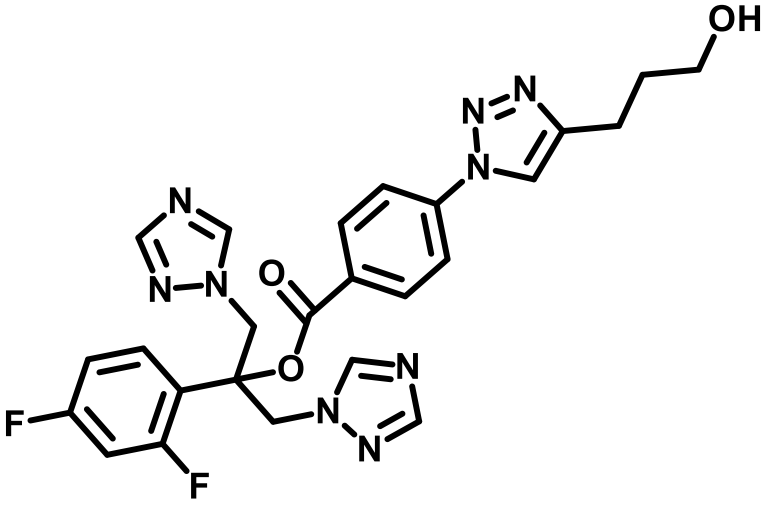

2-(2,4-difluorophenyl)-1,3-di(1H-1,2,4-triazol-1-yl)propan-2-yl 4-(4-(3-hydroxypropyl)-1H-1,2,3-triazol-1-yl)benzoate

Figure S37: LC-MS (ESI)

Figure S38: HRMS (ESI), calc/found m/z, Δ ppm

Figure S39: ^{1}H NMR (500 MHz, $CDCl_3$)

Figure S40: ^{13}C NMR (125 MHz, $CDCl_3$)

Figure S41: DEPT-135 (125 MHz, $CDCl_3$)

Figure S42: ^{19}F NMR (471 MHz, $CDCl_3$)

SHIMADZU LabSolutions

Analysis Report

Sample Name	: MJ44		
Sample ID	:		
Data Filename	: MJ44_MeOH_70-15m-03_(150-1500da)_18-02-2026_5.lcd		
Method Filename	: MeOH_70-15m-03_(150-1500da).lcm		
Batch Filename	: 18-02-2026.lcb		
Vial #	: 3-9	Sample Type	: Unknown
Injection Volume	: 0.1 uL		
Date Acquired	: 2/18/2026 2:24:17 PM	Acquired by	: System Administrator
Date Processed	: 2/18/2026 2:39:19 PM	Processed by	: System Administrator

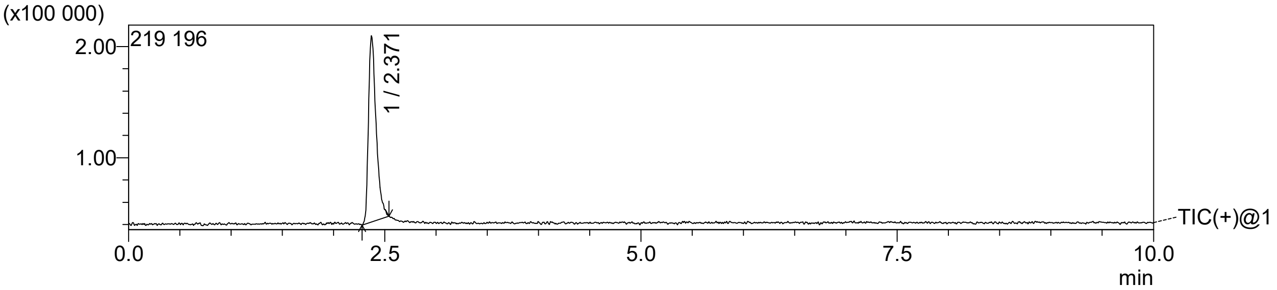

MASS Peak Table TIC

Peak#	Ret. Time	m/z	Area%
1	2.371	TIC	100.000
Total			100.000

MS Spectrum

Line#:1 R.Time:----(Scan#:----)
MassPeaks:79
Spectrum Mode:Averaged 2.365-2.375(474-476) Base Peak:536(91225)
BG Mode:Calc Segment 1 - Event 1

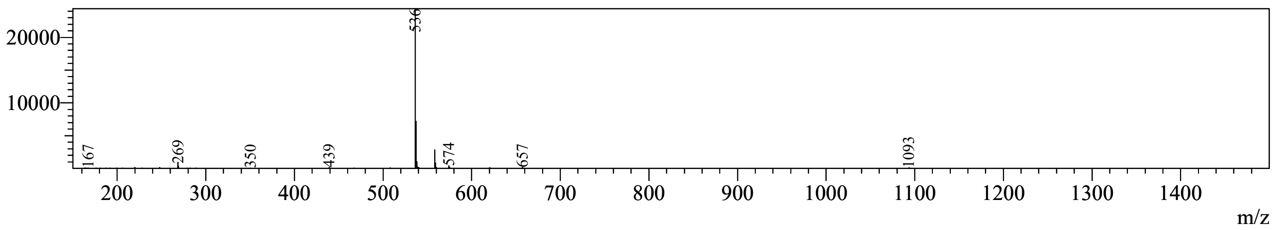

Figure S37. LC-MS (ESI)

Formula Predictor Report

Printed at 19.02.2026 14:15:08

Formula Predictor Result	**C25 H23 N9 O3 F2**
Mass	536.19664
Error Margin	60 ppm
DBE Range	Not Used
Electron Ions	Both configurations
HC Ratio	Not Used
Nitrogen Rule	Used

#	Score	Pred. (M)	Pred. m/z	Meas. m/z	Diff. (mDa)	Formulae (M)	Ion	Diff. (ppm)	Iso Score	DBE
1	95.42	535.18919	536.19647	536.19664	0.17	C25 H23 N9 O3 F2	[M+H]+	0.320	94.91	18.0

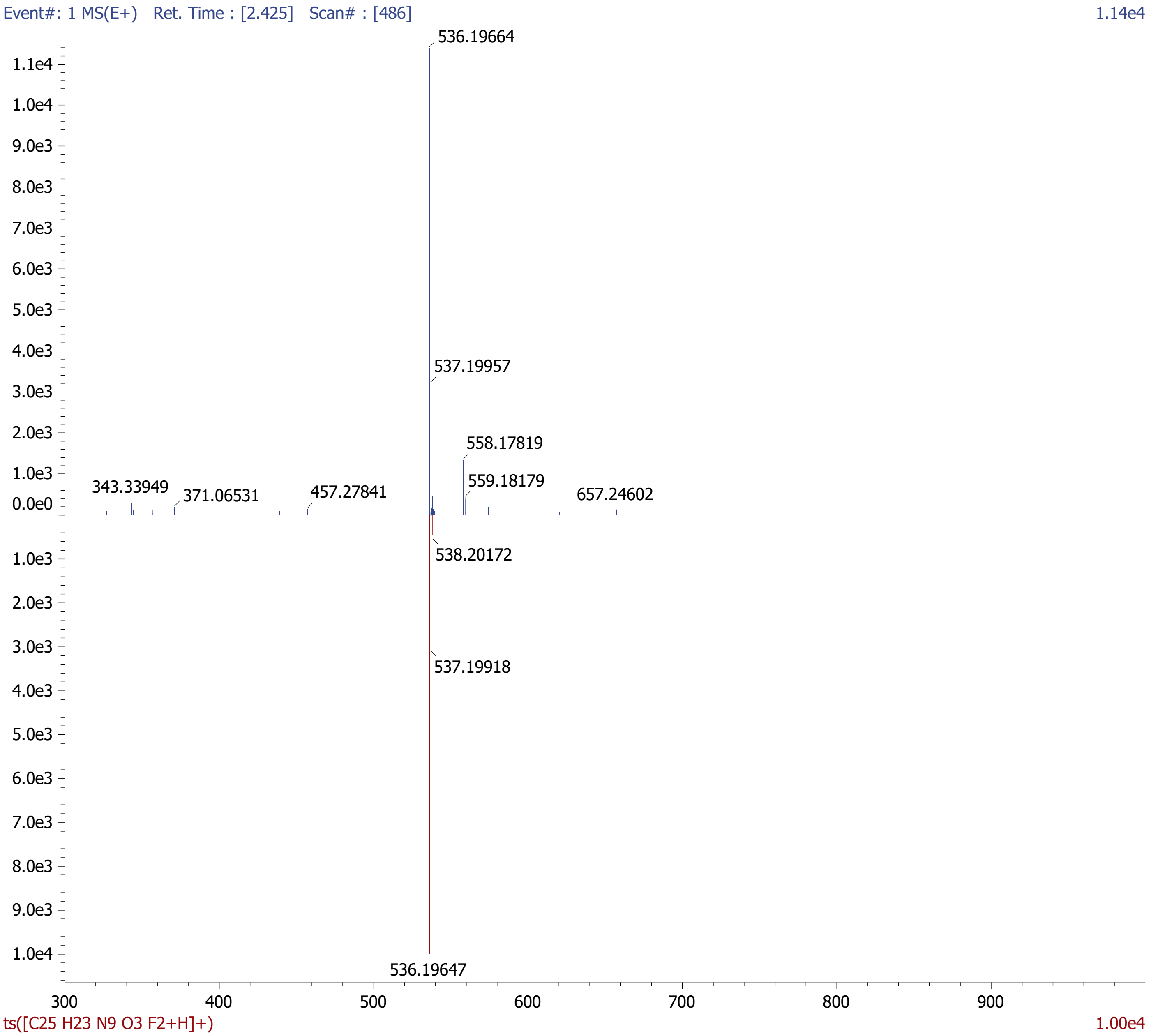

Figure S38. HRMS (ESI), calc/found m/z, Δ ppm

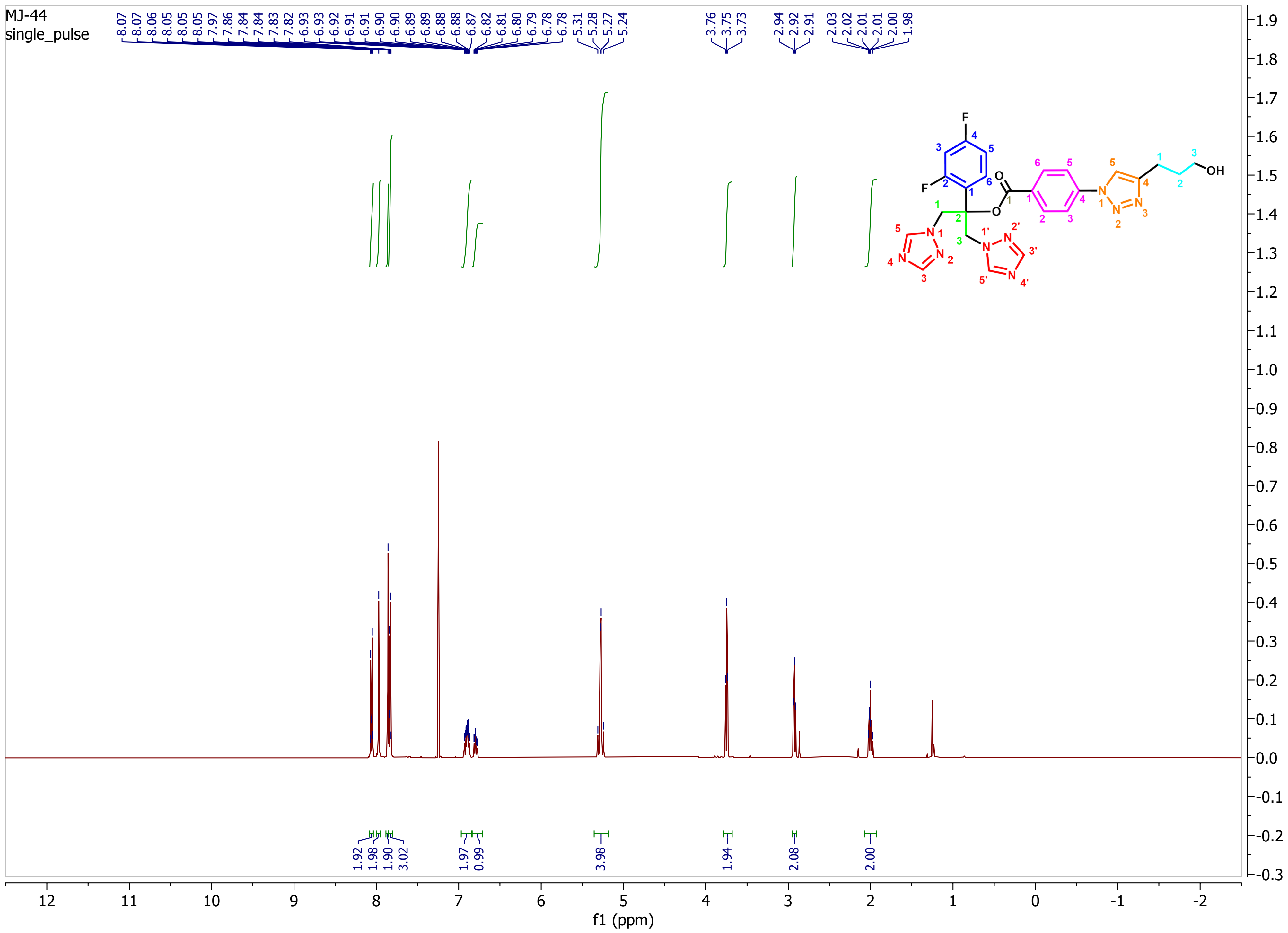

Figure S39. ^{1}HNMR (500 MHz, $CDCl_3$)

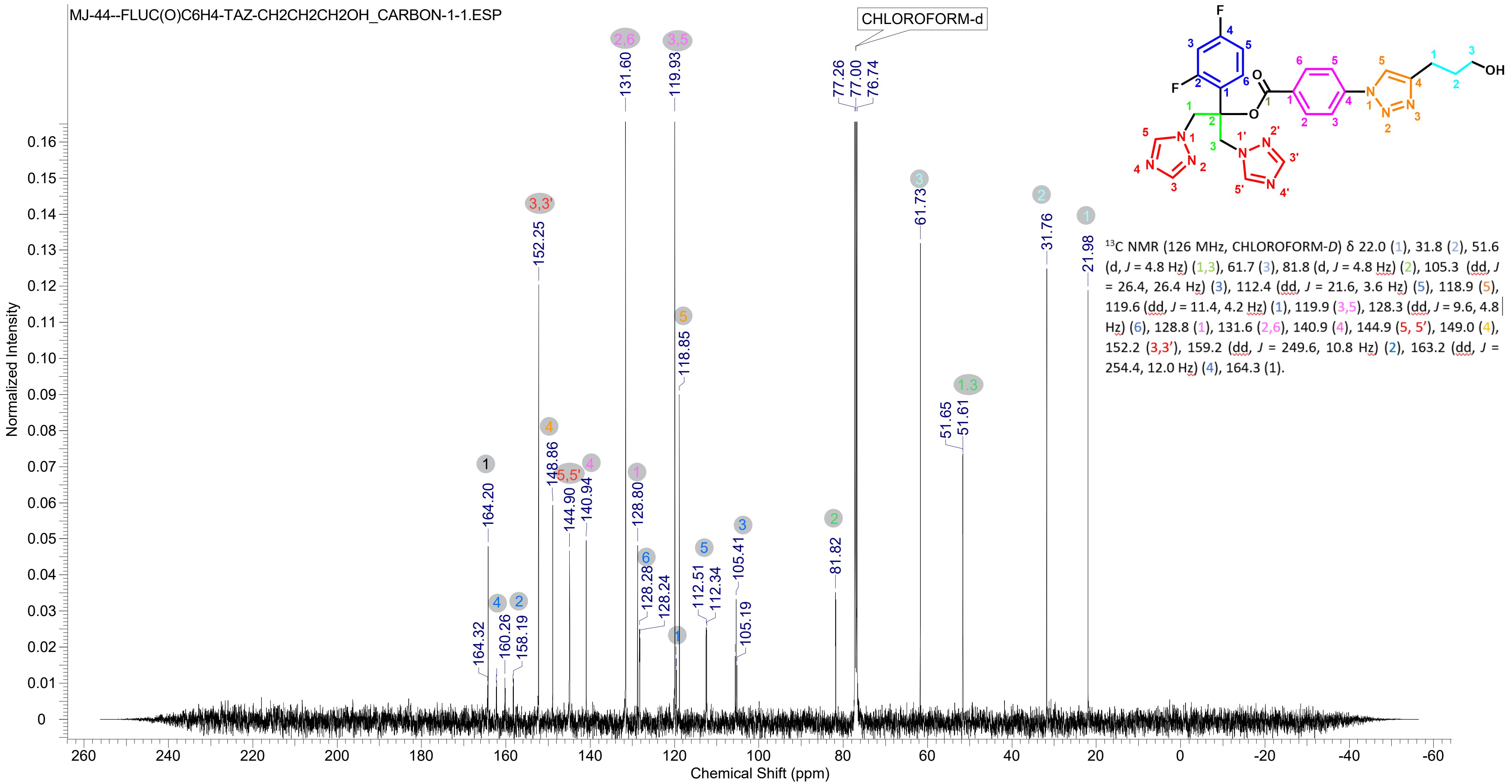

Figure S40. ^{13}C NMR (125 Mhz, $CDCl_3$)

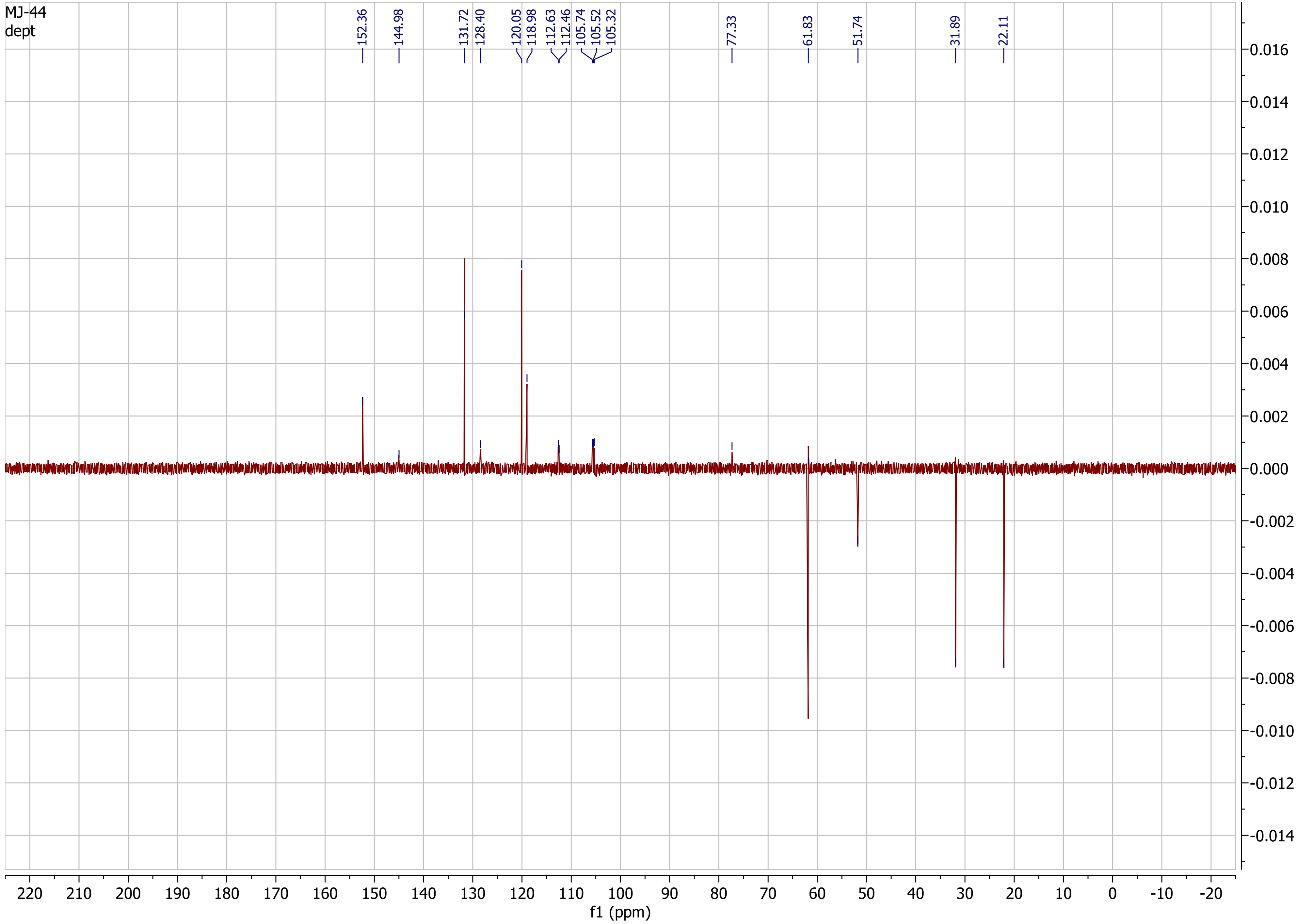

Figure S41. DEPT-135 (125 MHz, $CDCl_3$)

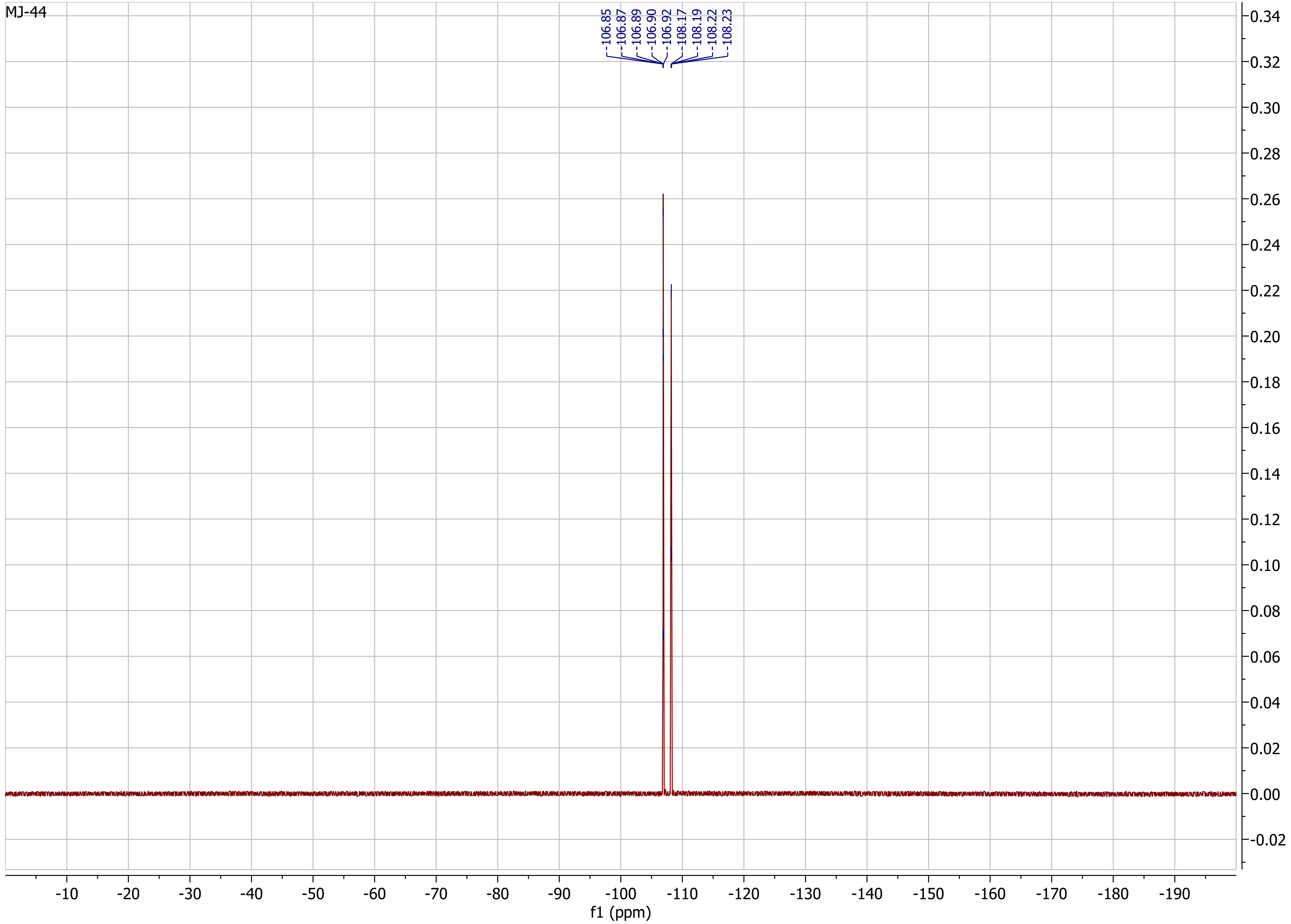

Figure S42. 19F NMR (471 MHz, $CDCl_3$)

S8. 3f

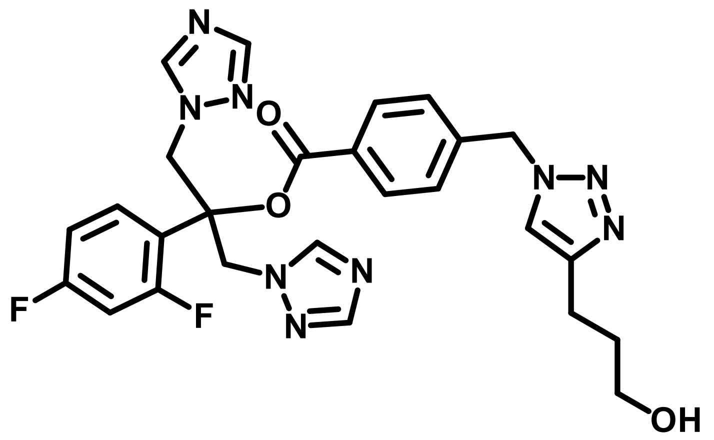

2-(2,4-difluorophenyl)-1,3-di(1H-1,2,4-triazol-1-yl)propan-2-yl 4-((4-(3-hydroxypropyl)-1H-1,2,3-triazol-1-yl)methyl)benzoate

Figure S43: LC-MS (ESI)

Figure S44: HRMS (ESI), calc/found m/z, Δppm

Figure S45: ^{1}H NMR (500 MHz, $CDCl_3$)

Figure S46: ^{13}C NMR (125 MHz, $CDCl_3$)

Figure S47: DEPT-135 (125 MHz, $CDCl_3$)

Figure S48: ^{19}F NMR (471 MHz, $CDCl_3$)

SHIMADZU LabSolutions

Analysis Report

Sample Name	: MJ136(v162)		
Sample ID	:		
Data Filename	: MJ136(v162)_MeOH_70-15m-03_(150-1500da)_18-02-2026_7.lcd		
Method Filename	: MeOH_70-15m-03_(150-1500da).lcm		
Batch Filename	: 18-02-2026.lcb		
Vial #	: 3-11	Sample Type	: Unknown
Injection Volume	: 0.1 uL		
Date Acquired	: 2/18/2026 2:55:10 PM	Acquired by	: System Administrator
Date Processed	: 2/18/2026 3:10:11 PM	Processed by	: System Administrator

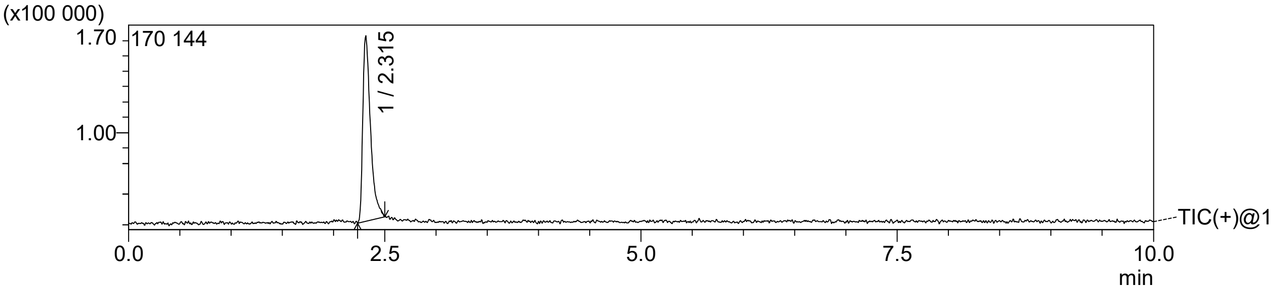

MASS Peak Table TIC

Peak#	Ret. Time	m/z	Area%
1	2.315	TIC	100.000
Total			100.000

MS Spectrum

Line#:1 R.Time:----(Scan#:----)
MassPeaks:62
Spectrum Mode:Averaged 2.305-2.315(462-464) Base Peak:550(62609)
BG Mode:Calc Segment 1 - Event 1

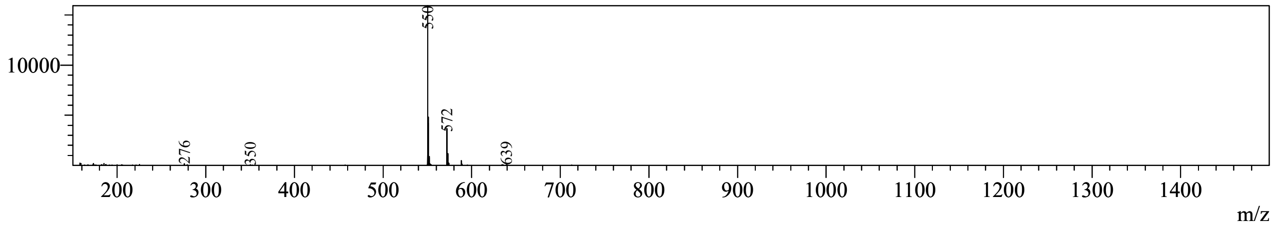

Figure S43. LC-MS (ESI)

Formula Predictor Report

Printed at 19.02.2026 14:20:38

Formula Predictor Result	**C26 H25 N9 O3 F2**
Mass	550.212506849
Error Margin	60 ppm
DBE Range	Not Used
Electron Ions	Both configurations
HC Ratio	Not Used
Nitrogen Rule	Used

#	Score	Pred. (M)	Pred. m/z	Meas. m/z	Diff. (mDa)	Formulae (M)	Ion	Diff. (ppm)	Iso Score	DBE
1	94.43	549.20484	550.21212	550.21251	0.39	C26 H25 N9 O3 F2	[M+H]+	0.706	93.82	18.0

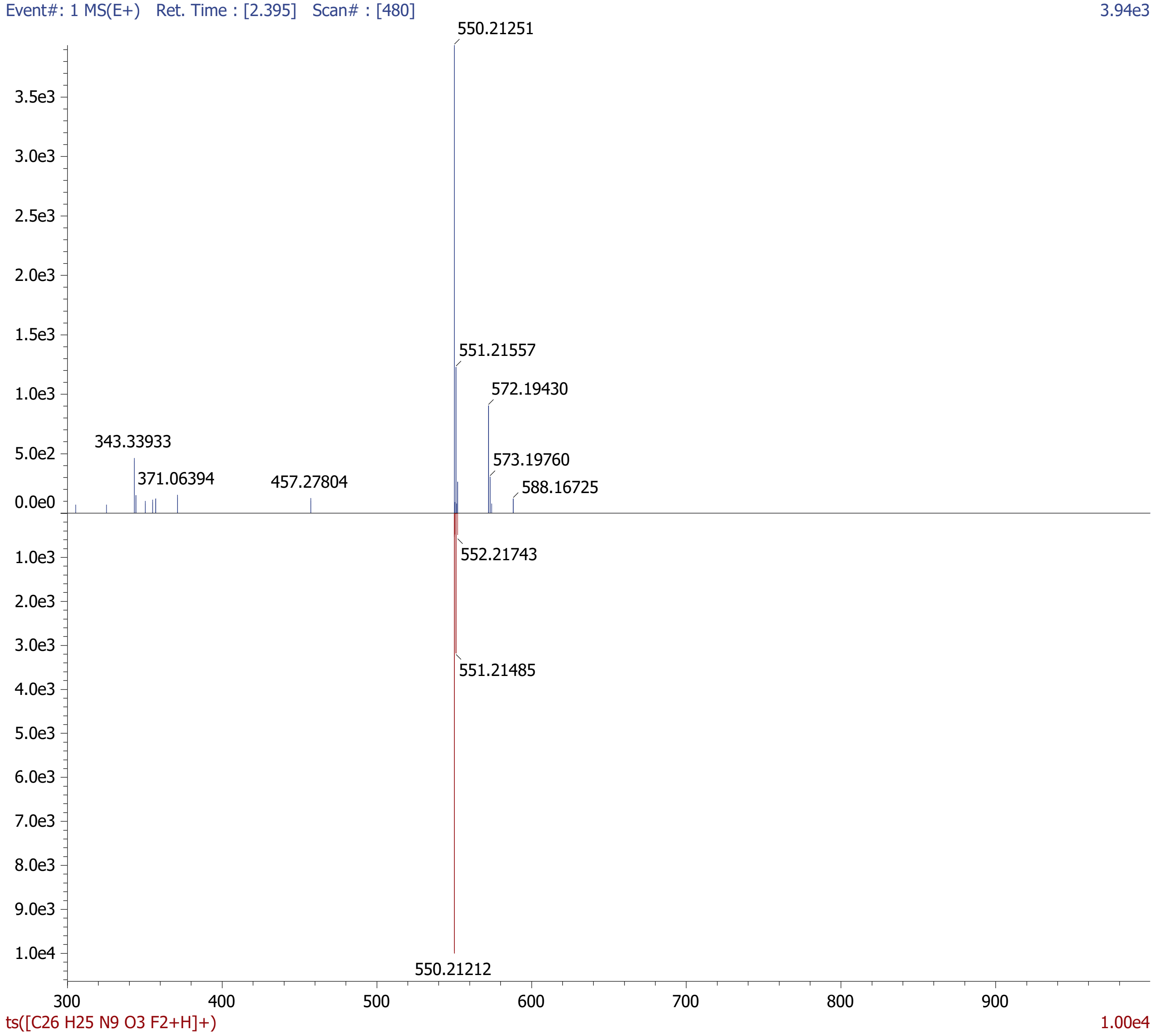

Figure S44. HRMS (ESI), calc/found m/z, Δ ppm

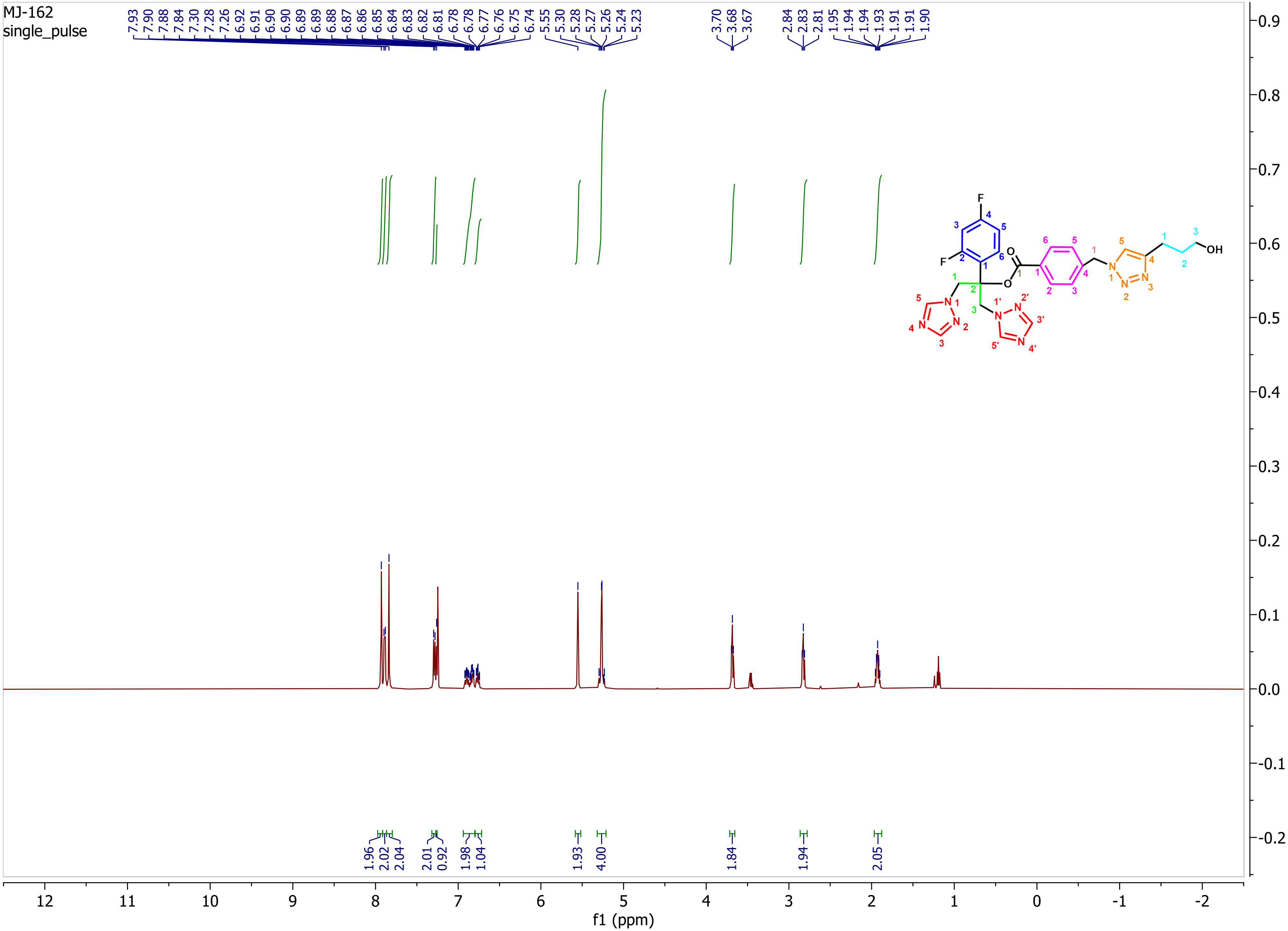

Figure S45. ^{1}HNMR (500 MHz, $CDCl_3$)

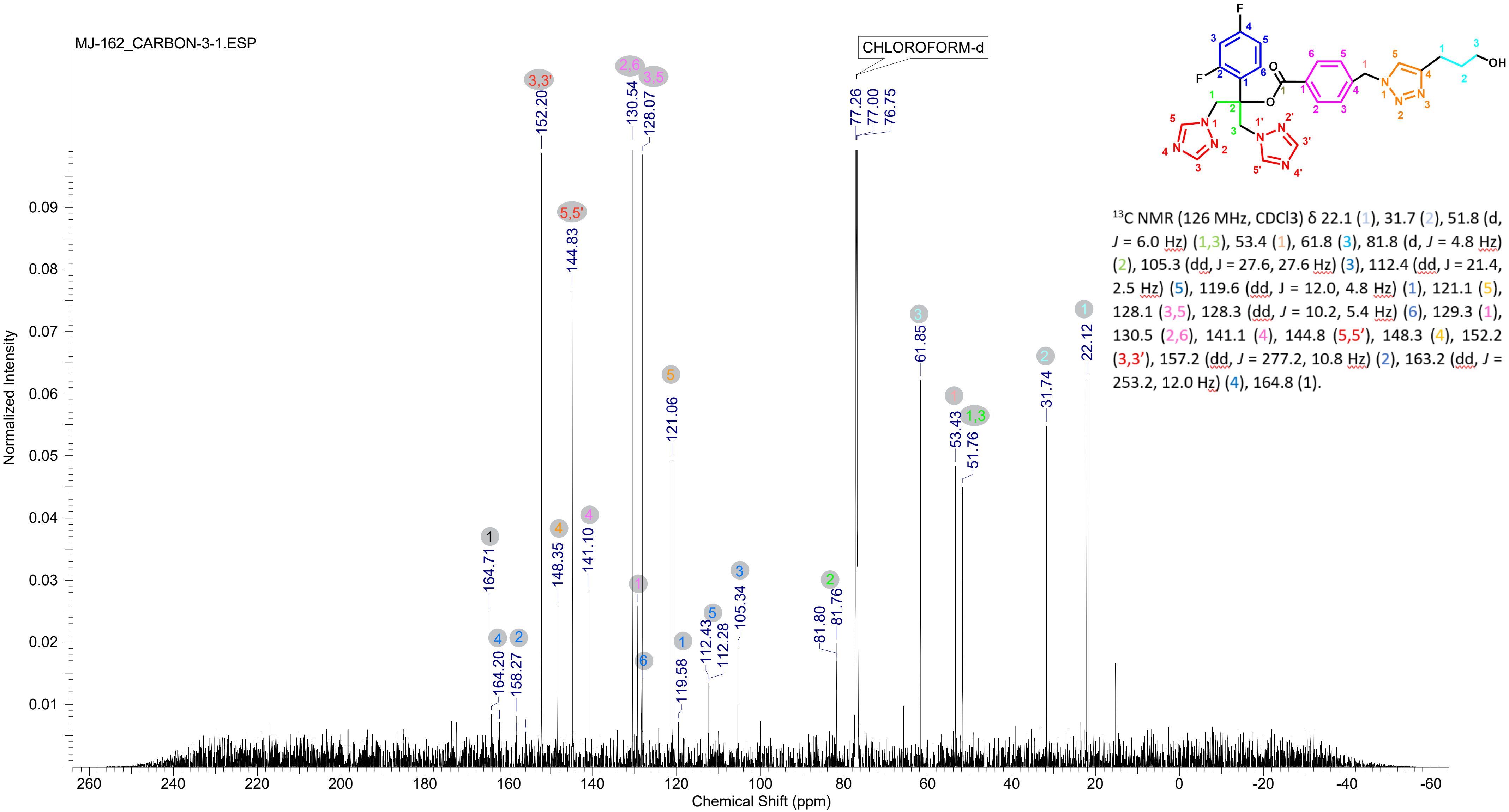

^{13}C NMR (126 MHz, CDCl3) δ 22.1 (1), 31.7 (2), 51.8 (d, J = 6.0 Hz) (1,3), 53.4 (1), 61.8 (3), 81.8 (d, J = 4.8 Hz) (2), 105.3 (dd, J = 27.6, 27.6 Hz) (3), 112.4 (dd, J = 21.4, 2.5 Hz) (5), 119.6 (dd, J = 12.0, 4.8 Hz) (1), 121.1 (5), 128.1 (3,5), 128.3 (dd, J = 10.2, 5.4 Hz) (6), 129.3 (1), 130.5 (2,6), 141.1 (4), 144.8 (5,5'), 148.3 (4), 152.2 (3,3'), 157.2 (dd, J = 277.2, 10.8 Hz) (2), 163.2 (dd, J = 253.2, 12.0 Hz) (4), 164.8 (1).

Figure S46. ^{13}C NMR (125 Mhz, $CDCl_3$)

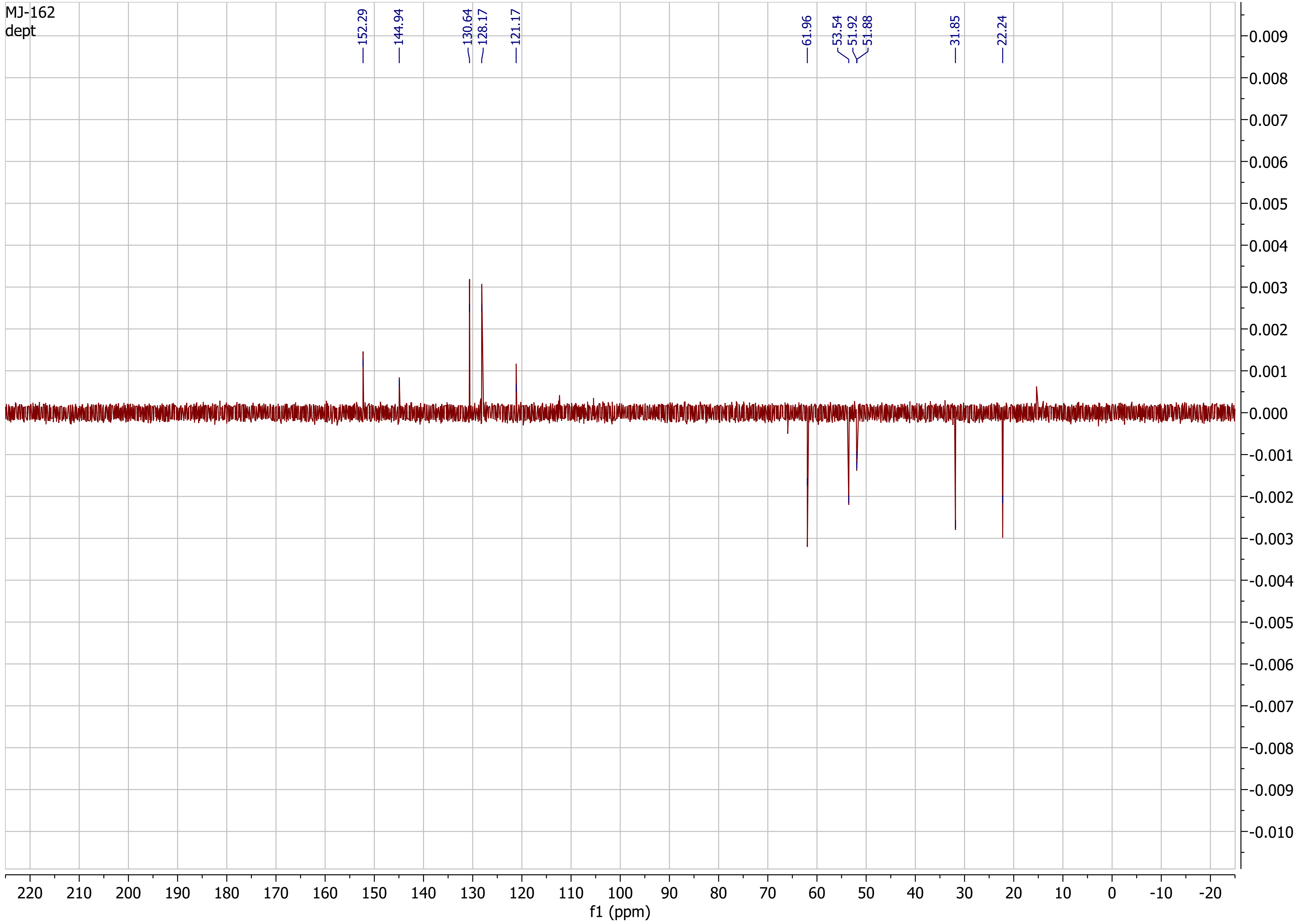

Figure S47. DEPT-135 (125 MHz, $CDCl_3$)

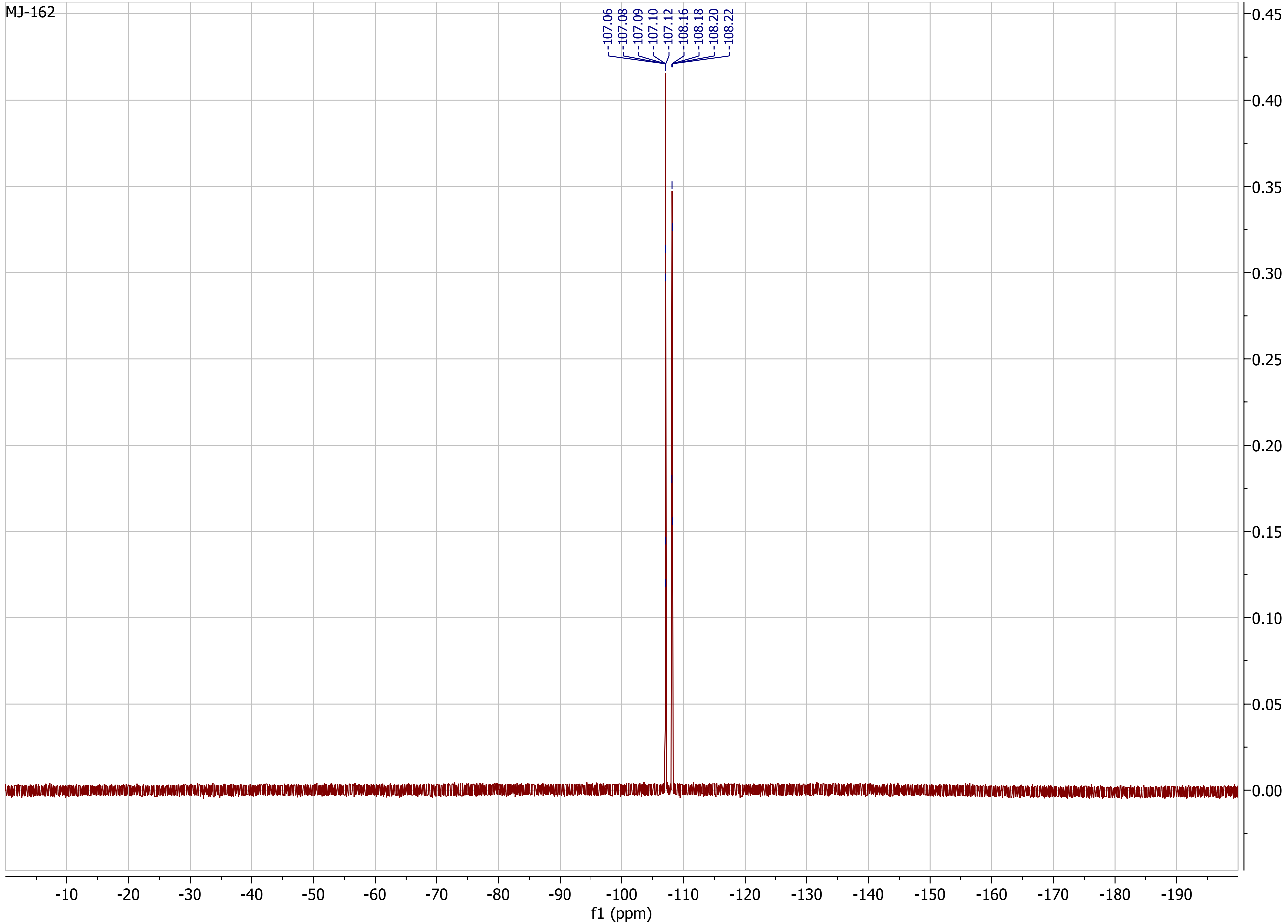

Figure S48. ^{19}F NMR (471 MHz, $CDCl_3$)

S9. 3g

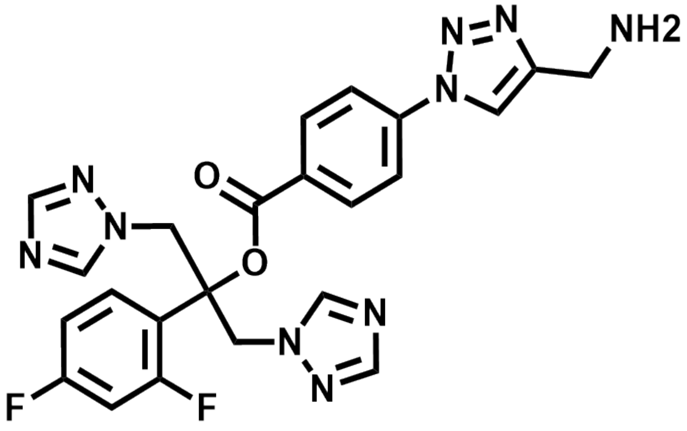

2-(2,4-difluorophenyl)-1,3-di(1H-1,2,4-triazol-1-yl)propan-2-yl 4-((4-(3-hydroxypropyl)-1H-1,2,3-triazol-1-yl)methyl)benzoate

Figure S49: LC-MS (ESI)

Figure S50: HRMS (ESI), calc/found m/z, Δ ppm

Figure S51: ^{1}H NMR (500 MHhz $CDCl_3$)

Figure S52: ^{13}C NMR (125 MHz, $CDCl_3$)

Figure S53: DEPT-135 (125 MHhz $CDCl_3$)

Figure S54: ^{19}F NMR (471 MHhz $CDCl_3$)

SHIMADZU LabSolutions

Analysis Report

Sample Name	: MJ-170		
Sample ID	:		
Data Filename	: MJ-170_MeOH_70-15m-03_(150-1500da)_05-02-2026_5.lcd		
Method Filename	: MeOH_70-15m-03_(150-1500da).lcm		
Batch Filename	: 05-02-2026.lcb		
Vial #	: 2-40	Sample Type	: Unknown
Injection Volume	: 0.1 uL		
Date Acquired	: 2/5/2026 5:29:08 PM	Acquired by	: System Administrator
Date Processed	: 2/5/2026 5:44:09 PM	Processed by	: System Administrator

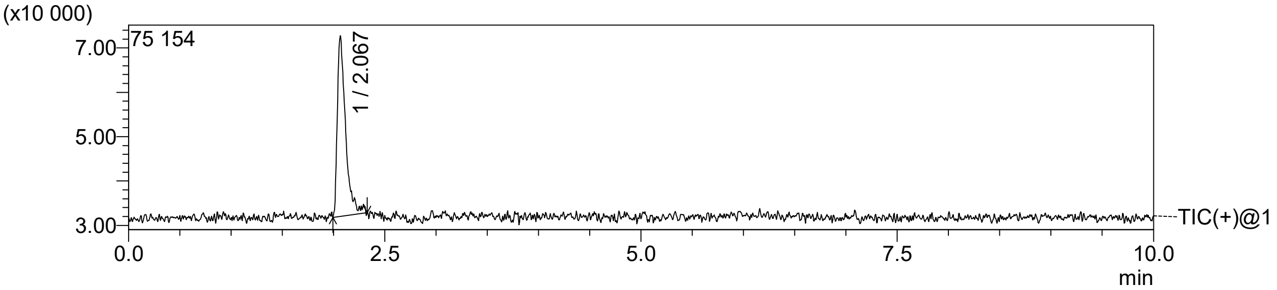

MASS Peak Table TIC

Peak#	Ret. Time	m/z	Area%
1	2.067	TIC	100.000
Total			100.000

MS Spectrum

Line#:1 R.Time:----(Scan#:----)
MassPeaks:37
Spectrum Mode:Averaged 2.060-2.070(413-415) Base Peak:507(26781)
BG Mode:Calc Segment 1 - Event 1

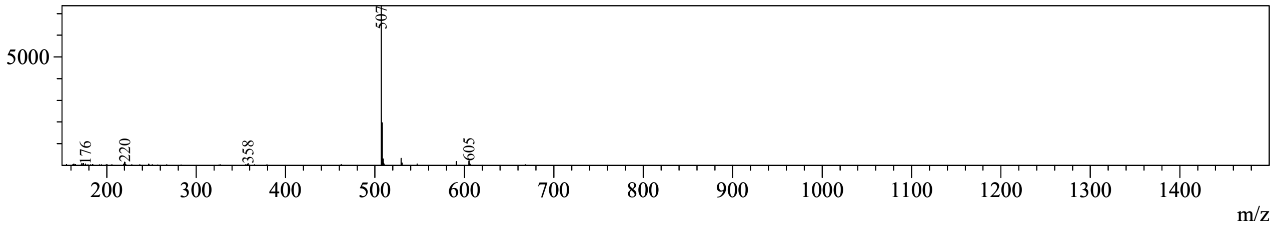

S49. LC-MS (ESI)

Formula Predictor Report

Printed at 06.02.2026 15:37:05

Formula Predictor Result	**C23 H20 N10 O2 F2**
Mass	507.18199
Error Margin	30 ppm
DBE Range	Not Used
Electron Ions	Both configurations
HC Ratio	Not Used
Nitrogen Rule	Used

#	Score	Pred. (M)	Pred. m/z	Meas. m/z	Diff. (mDa)	Formulae (M)	Ion	Diff. (ppm)	Iso Score	DBE
2	72.36	506.17388	507.18115	507.18199	0.84	C23 H20 N10 O2 F2	[M+H]+	1.651	69.29	18.0

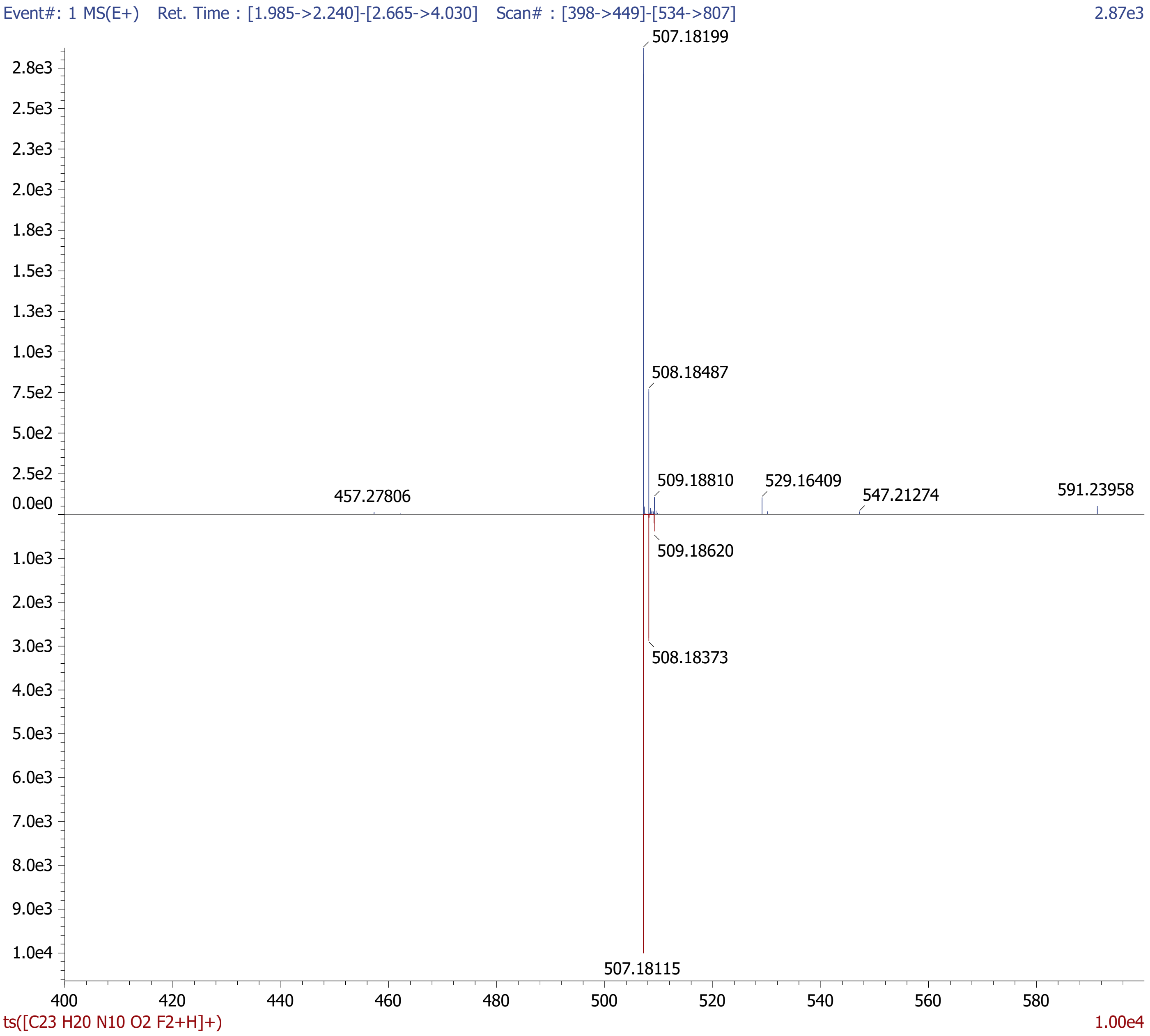

Figure S50. HRMS (ESI), calc/found m/z, Δ ppm

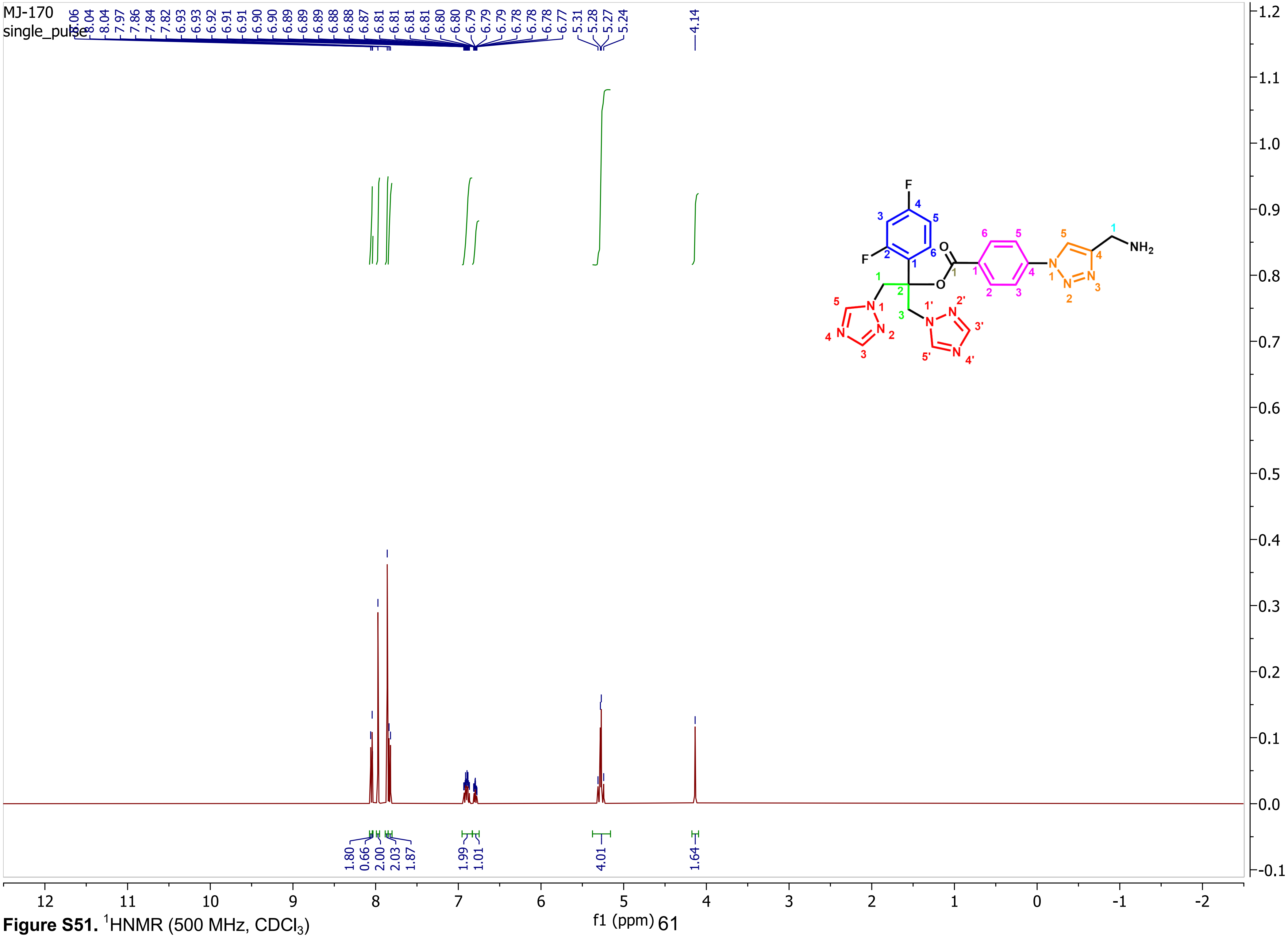

Figure S51. ^{1}HNMR (500 MHz, $CDCl_3$)

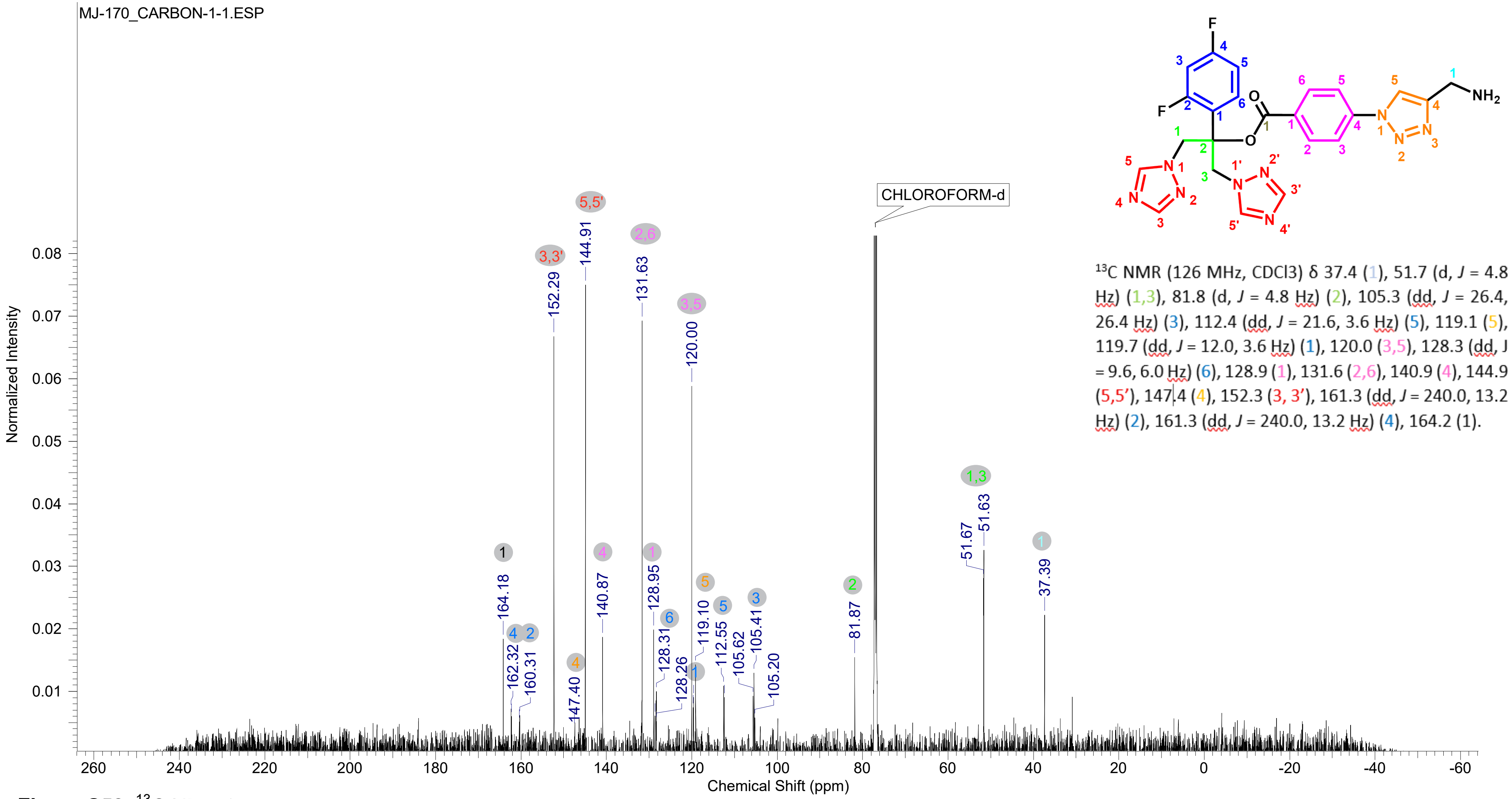

Figure S52. ^{13}C NMR (125 Mhz, $CDCl_3$)

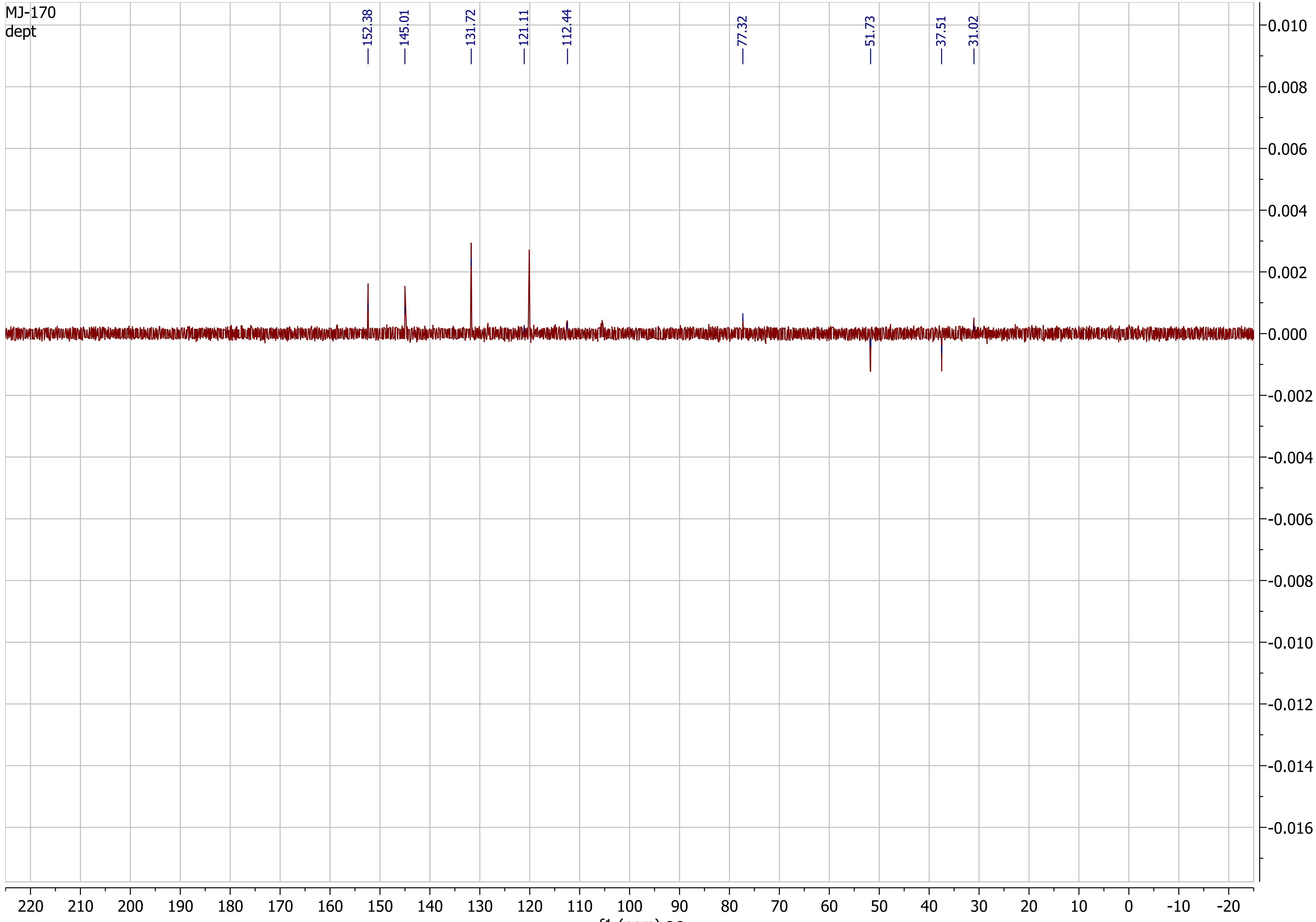

Figure S53. DEPT-135 (125 MHz, $CDCl_3$)

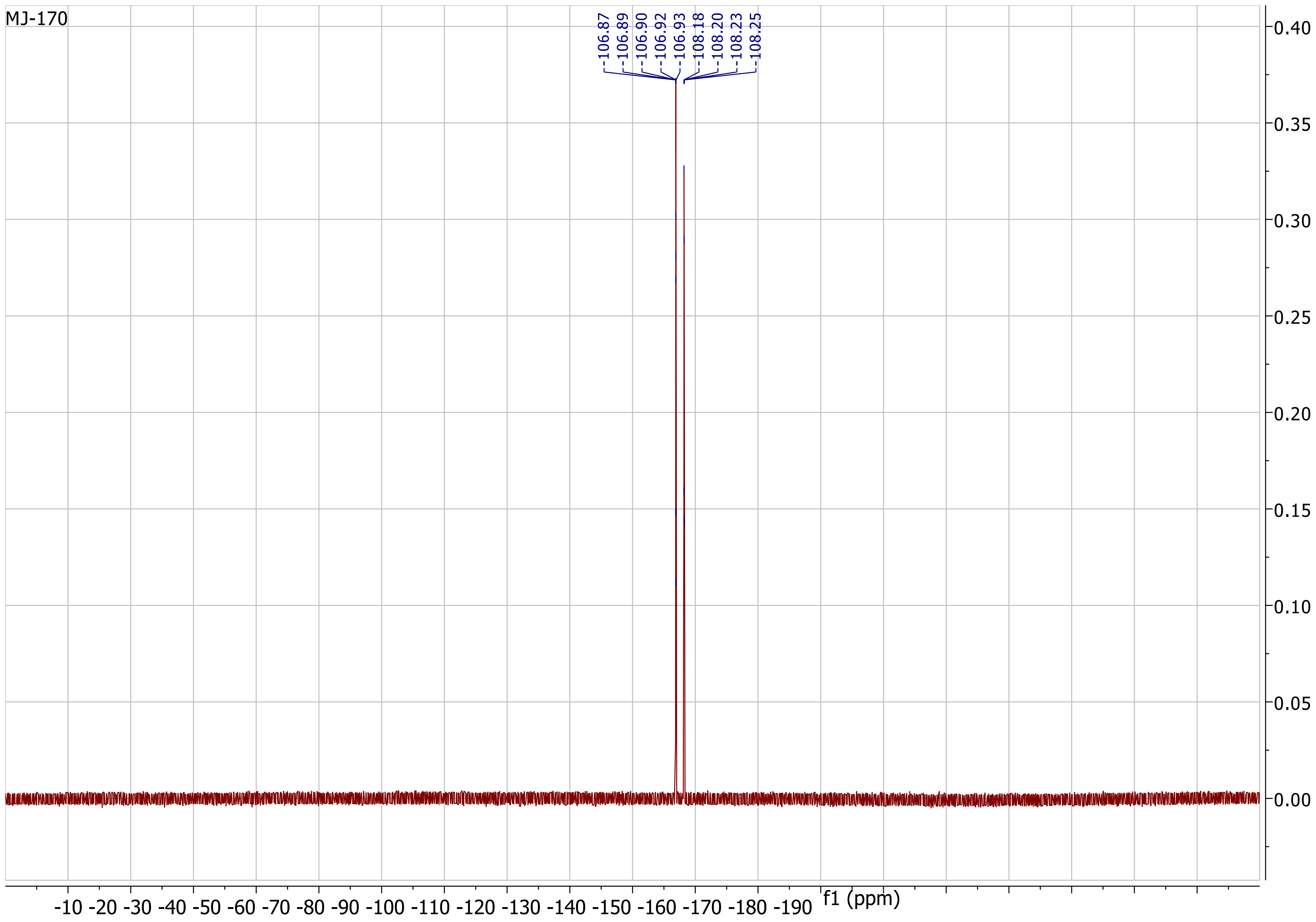

Figure S54. ^{19}F NMR (471 MHz, $CDCl_3$)

S9. 3g

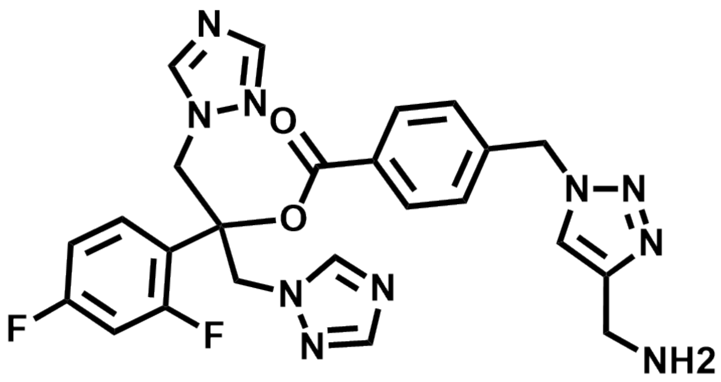

2-(2,4-difluorophenyl)-1,3-di(1H-1,2,4-triazol-1-yl)propan-2-yl 4-(4-(aminomethyl)-1H-1,2,3-triazol-1-yl)benzoate

Figure S55: LC-MS (ESI)
Figure S56: HRMS (ESI), calc/found m/z, Δ ppm SSP
Figure S57: ^{1}H NMR (500 MHz, $CDCl_3$)
Figure S58: ^{13}C NMR (125 MHz, $CDCl_3$)
Figure S59: DEPT-135 (125 MHz, $CDCl_3$)
Figure S60:^{19}F NMR (471 MHz, $CDCl_3$

SHIMADZU LabSolutions

Analysis Report

Sample Name : MJ-171
Sample ID :
Data Filename : MJ-171_MeOH_70-15m-03_(150-1500da)_05-02-2026_6.lcd
Method Filename : MeOH_70-15m-03_(150-1500da).lcm
Batch Filename : 05-02-2026.lcb
Vial # : 2-41
Sample Type : Unknown
Injection Volume : 0.1 uL
Date Acquired : 2/5/2026 5:44:33 PM
Acquired by : System Administrator
Date Processed : 2/5/2026 5:59:34 PM
Processed by : System Administrator

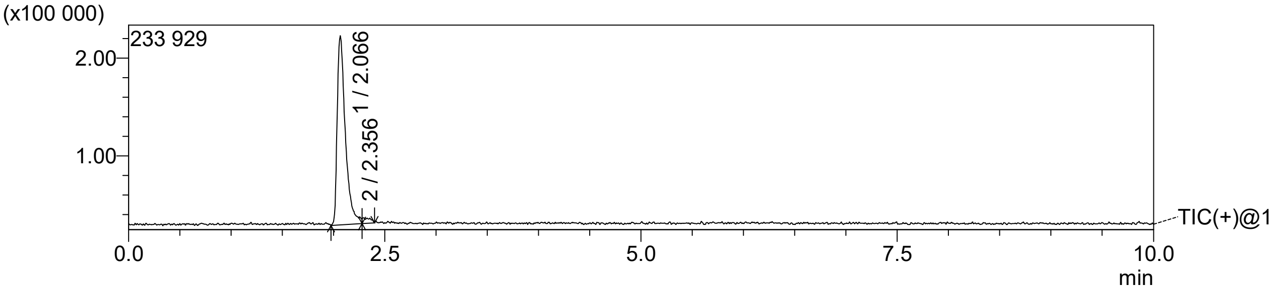

MASS Peak Table TIC

Peak#	Ret. Time	m/z	Area%
1	2.066	TIC	97.266
2	2.356	TIC	2.734
Total			100.000

MS Spectrum

Line#:1 R.Time:----(Scan#:----)
MassPeaks:95
Spectrum Mode:Averaged 2.060-2.070(413-415) Base Peak:521(104756)
BG Mode:Calc Segment 1 - Event 1

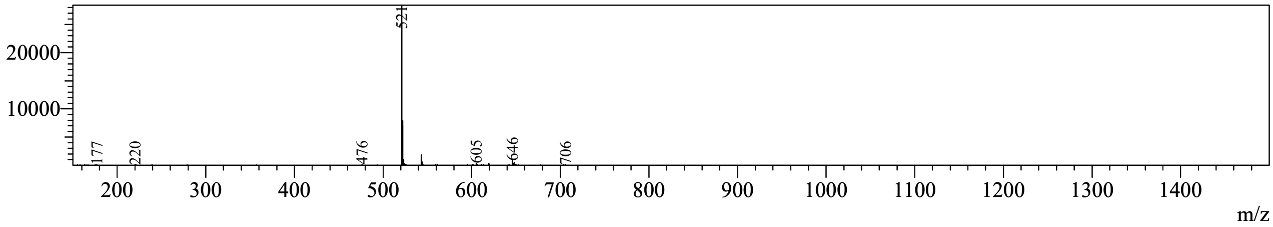

Line#:2 R.Time:----(Scan#:----)
MassPeaks:36
Spectrum Mode:Averaged 2.350-2.360(471-473) Base Peak:603(948)
BG Mode:Calc Segment 1 - Event 1

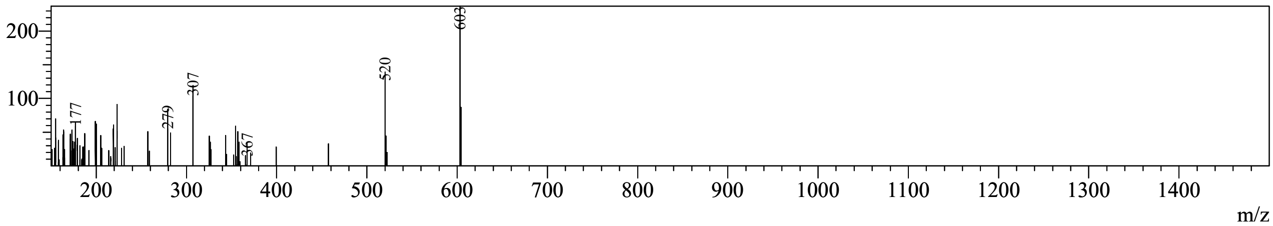

S55. LC-MS (ESI)

Formula Predictor Report

Printed at 06.02.2026 15:39:21

Formula Predictor Result	**C24 H22 N10 O2 F2**
Mass	521.19770
Error Margin	30 ppm
DBE Range	Not Used
Electron Ions	Both configurations
HC Ratio	Not Used
Nitrogen Rule	Used

#	Score	Pred. (M)	Pred. m/z	Meas. m/z	Diff. (mDa)	Formulae (M)	Ion	Diff. (ppm)	Iso Score	DBE
2	72.82	520.18953	521.19680	521.19770	0.90	C24 H22 N10 O2 F2	[M+H]+	1.721	69.81	18.0

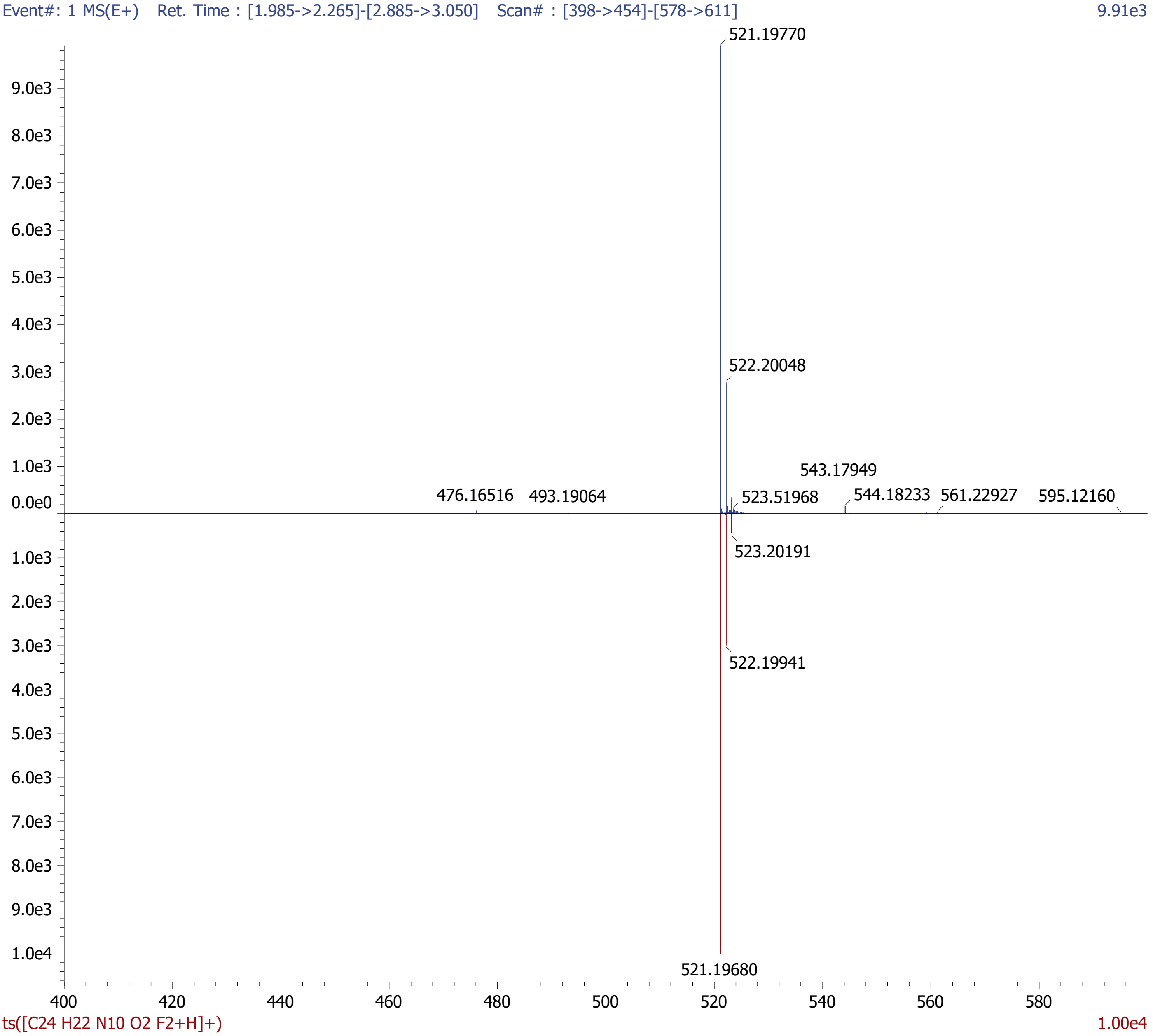

Figure S56. HRMS (ESI), calc/found m/z, Δ ppm

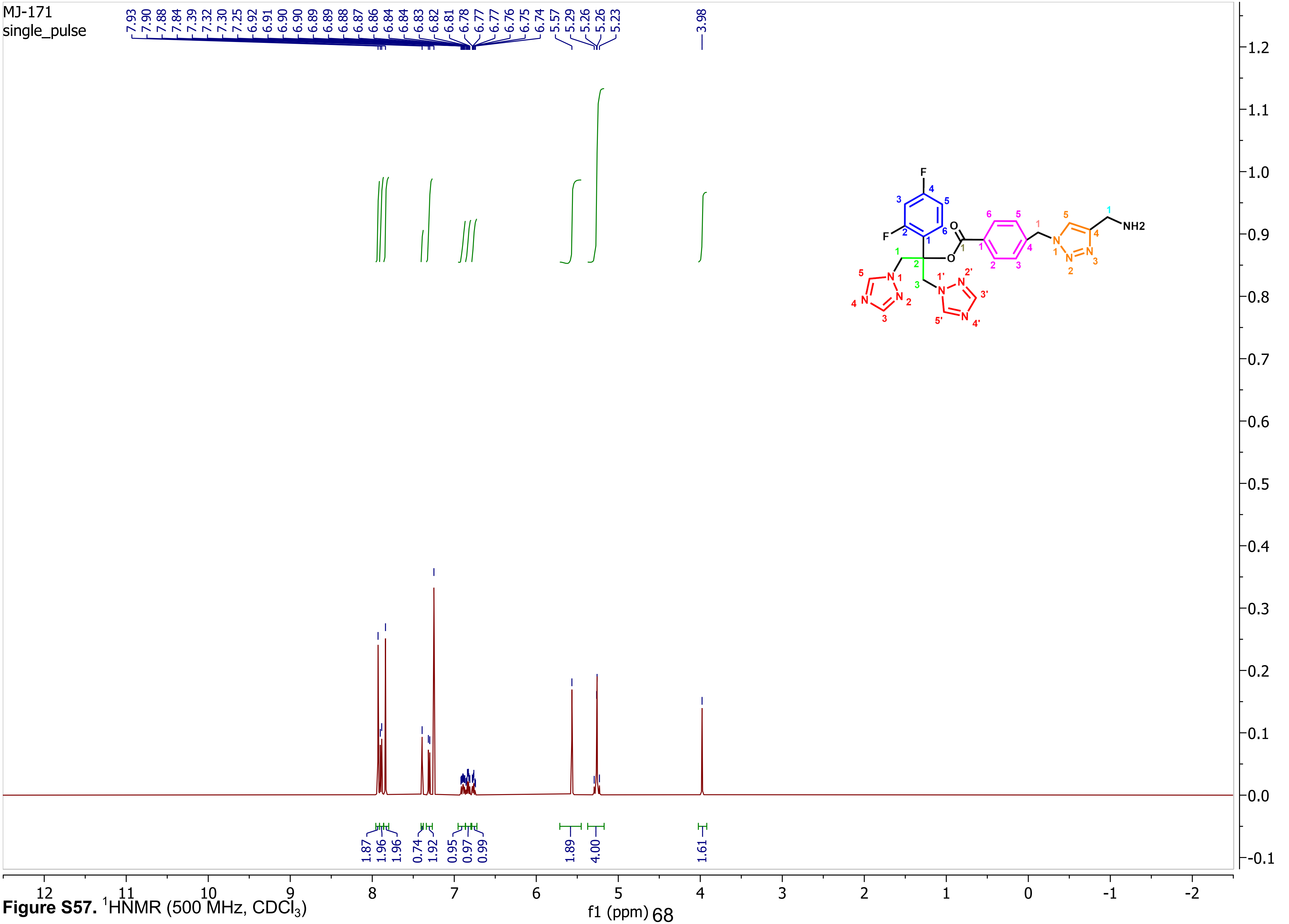

Figure S57. ^{1}HNMR (500 MHz, $CDCl_3$)

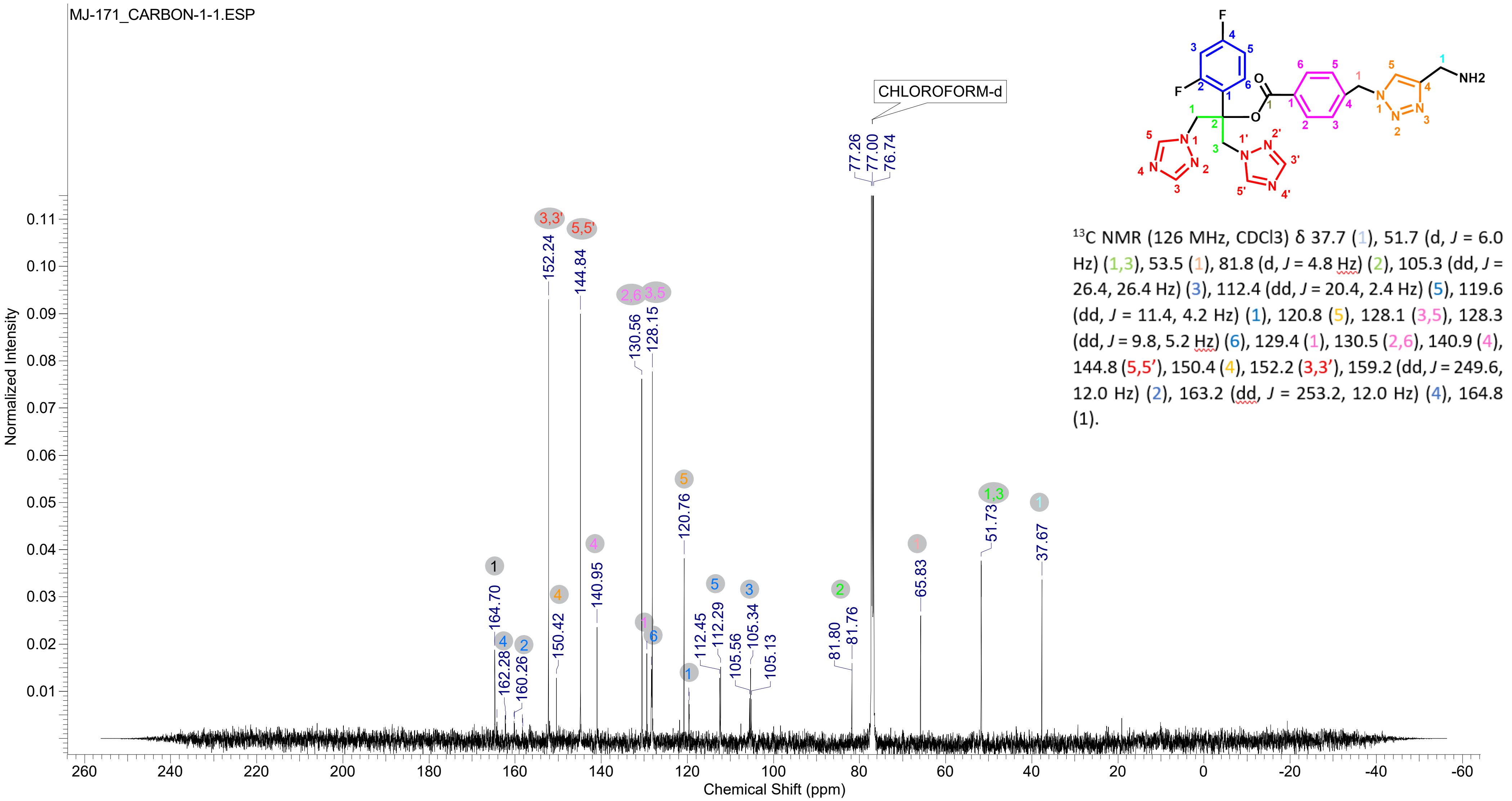

^{13}C NMR (126 MHz, CDCl3) δ 37.7 (1), 51.7 (d, *J* = 6.0 Hz) (1,3), 53.5 (1), 81.8 (d, *J* = 4.8 Hz) (2), 105.3 (dd, *J* = 26.4, 26.4 Hz) (3), 112.4 (dd, *J* = 20.4, 2.4 Hz) (5), 119.6 (dd, *J* = 11.4, 4.2 Hz) (1), 120.8 (5), 128.1 (3,5), 128.3 (dd, *J* = 9.8, 5.2 Hz) (6), 129.4 (1), 130.5 (2,6), 140.9 (4), 144.8 (5,5'), 150.4 (4), 152.2 (3,3'), 159.2 (dd, *J* = 249.6, 12.0 Hz) (2), 163.2 (dd, *J* = 253.2, 12.0 Hz) (4), 164.8 (1).

Figure S58. ^{13}C NMR (125 Mhz, $CDCl_3$)

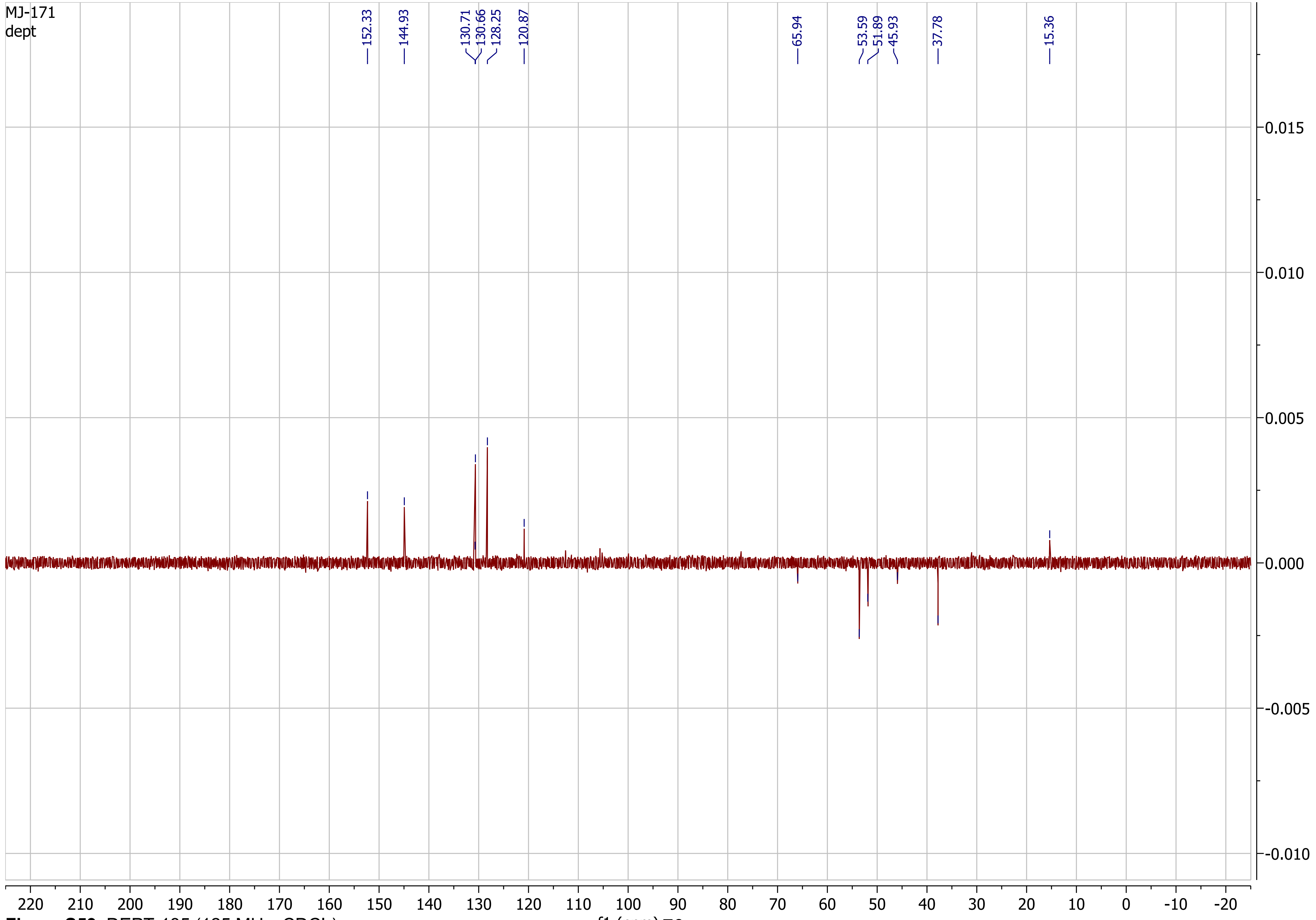

Figure S59. DEPT-135 (125 MHz, $CDCl_3$)

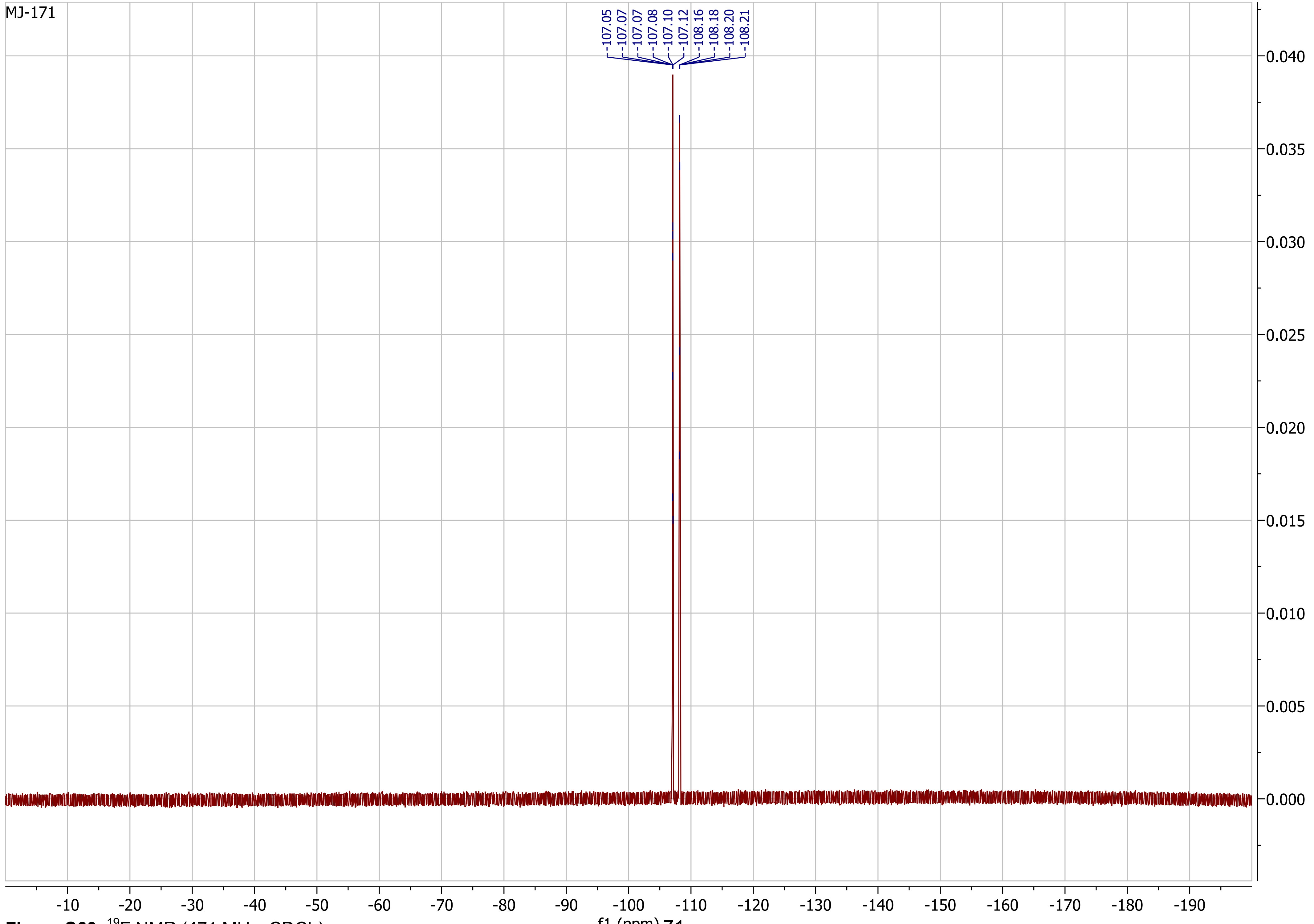

Figure S60. ^{19}F NMR (471 MHz, $CDCl_3$)

S9. 4-azidobenzoic acid

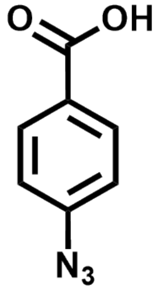

4-azidobenzoic acid

Figure S61: ^{1}H NMR (500 MHz,DMSO-d_6)

Figure S62: ^{13}C NMR (125 Mhz, DMSO-d_6)

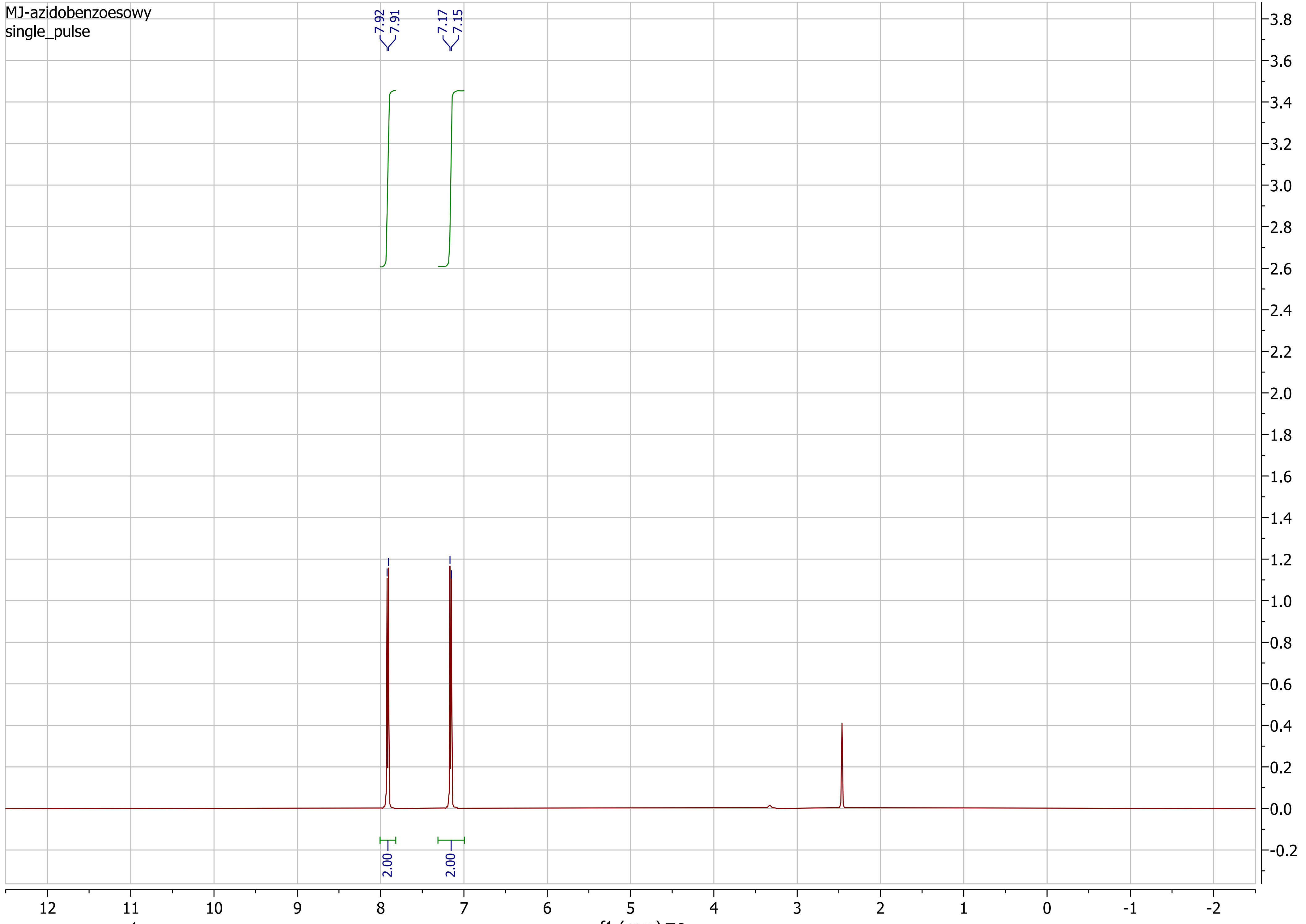

Figure S61. ^{1}H NMR 4-azidobenzoic acid

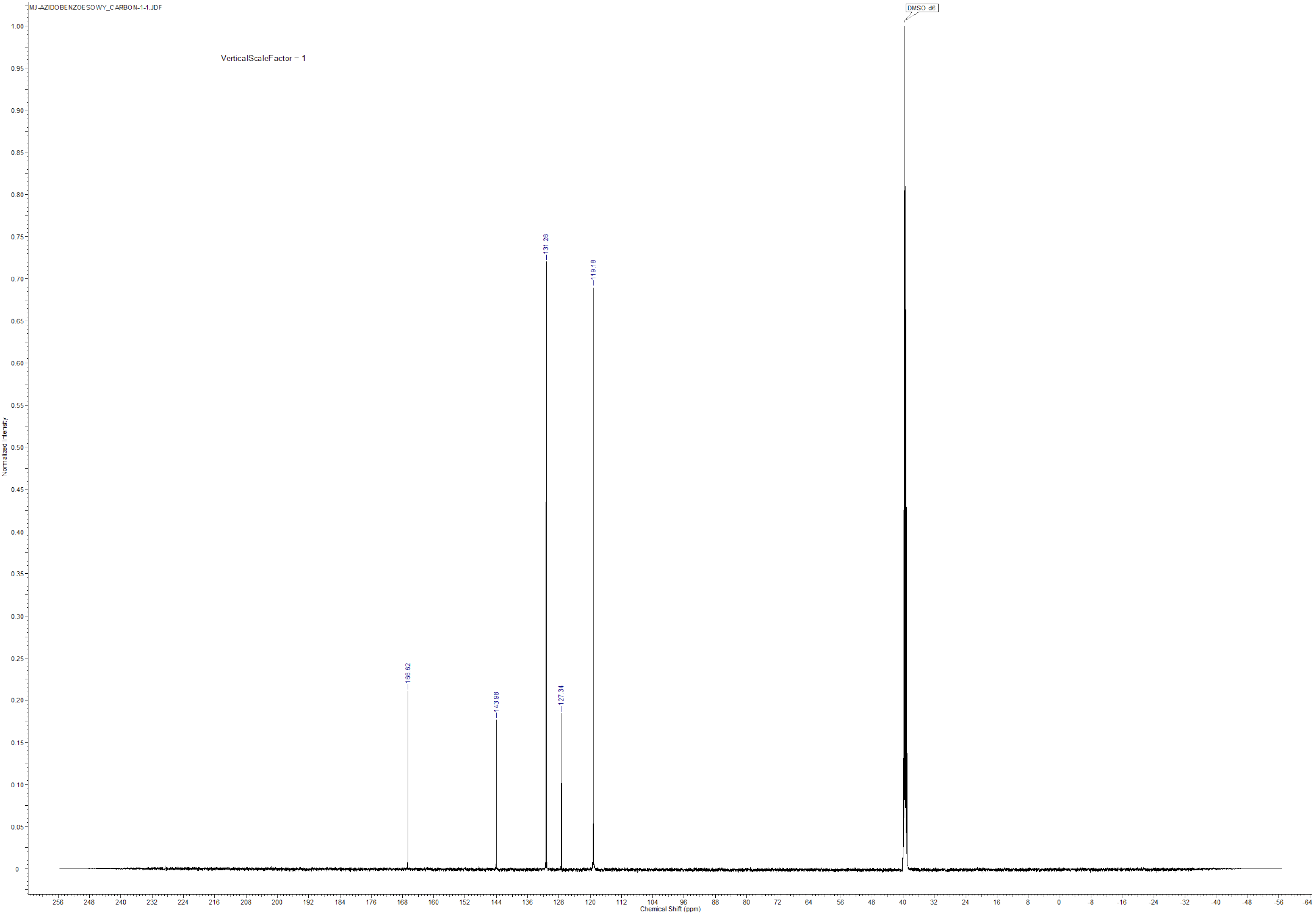

Figure S62. ^{13}C NMR 4-azido benzoic acid

S10. 4-(azidomethyl)benzoic acid

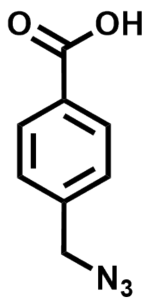

4-(azidomethyl)benzoic acid

Figure S63: ^{1}H NMR (500 MHz, $CDCl_3$)

Figure S64: ^{13}C NMR (125 Mhz, $CDCl_3$)

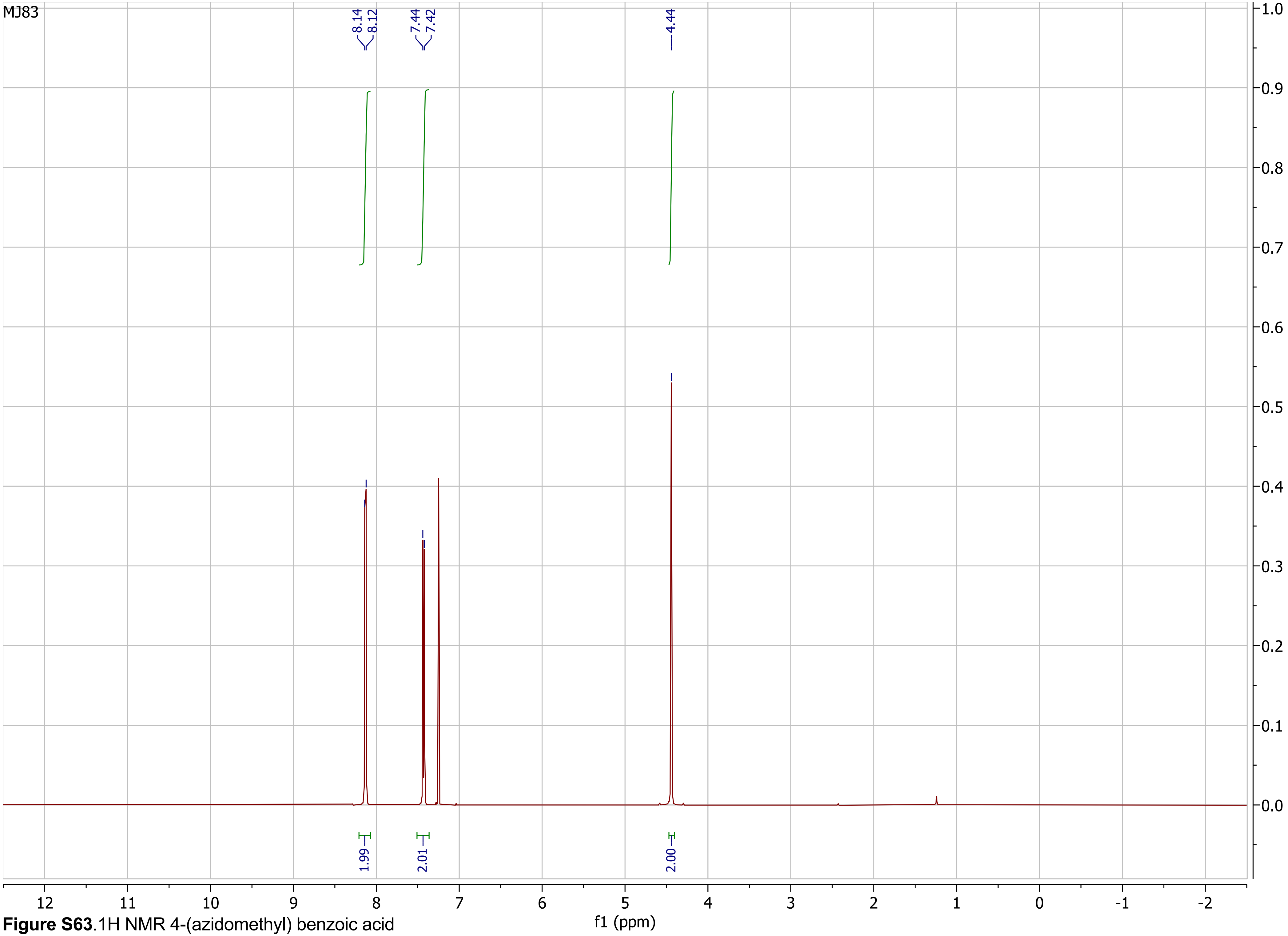

Figure S63.1H NMR 4-(azidomethyl) benzoic acid

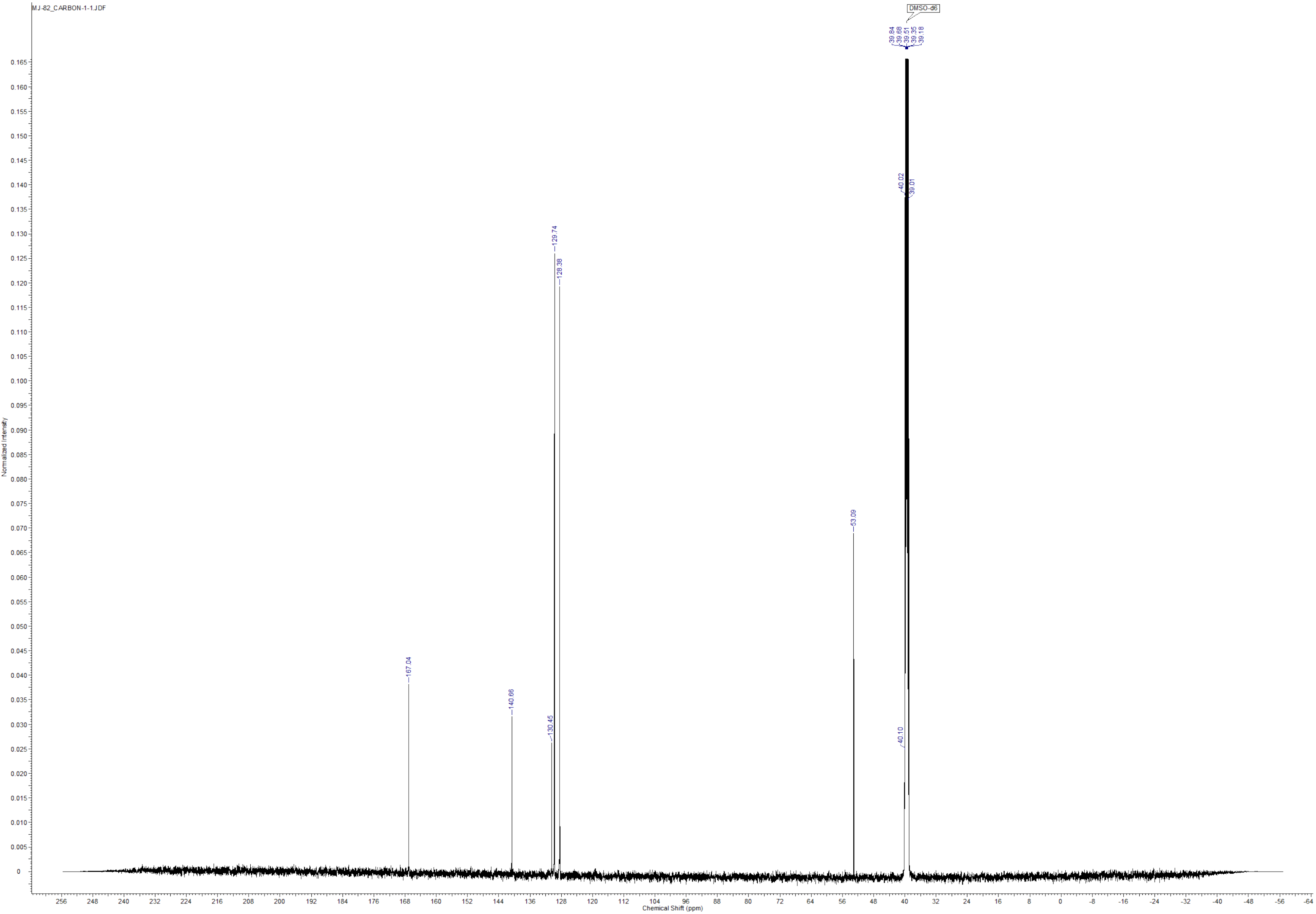

Figure S64.^{13}C NMR 4-(azidomethyl) benzoic acid

S11. Copper(II) palmitate

$Cu(C_{15}H_{31}COO)_2$

Figure S65: IR spectra

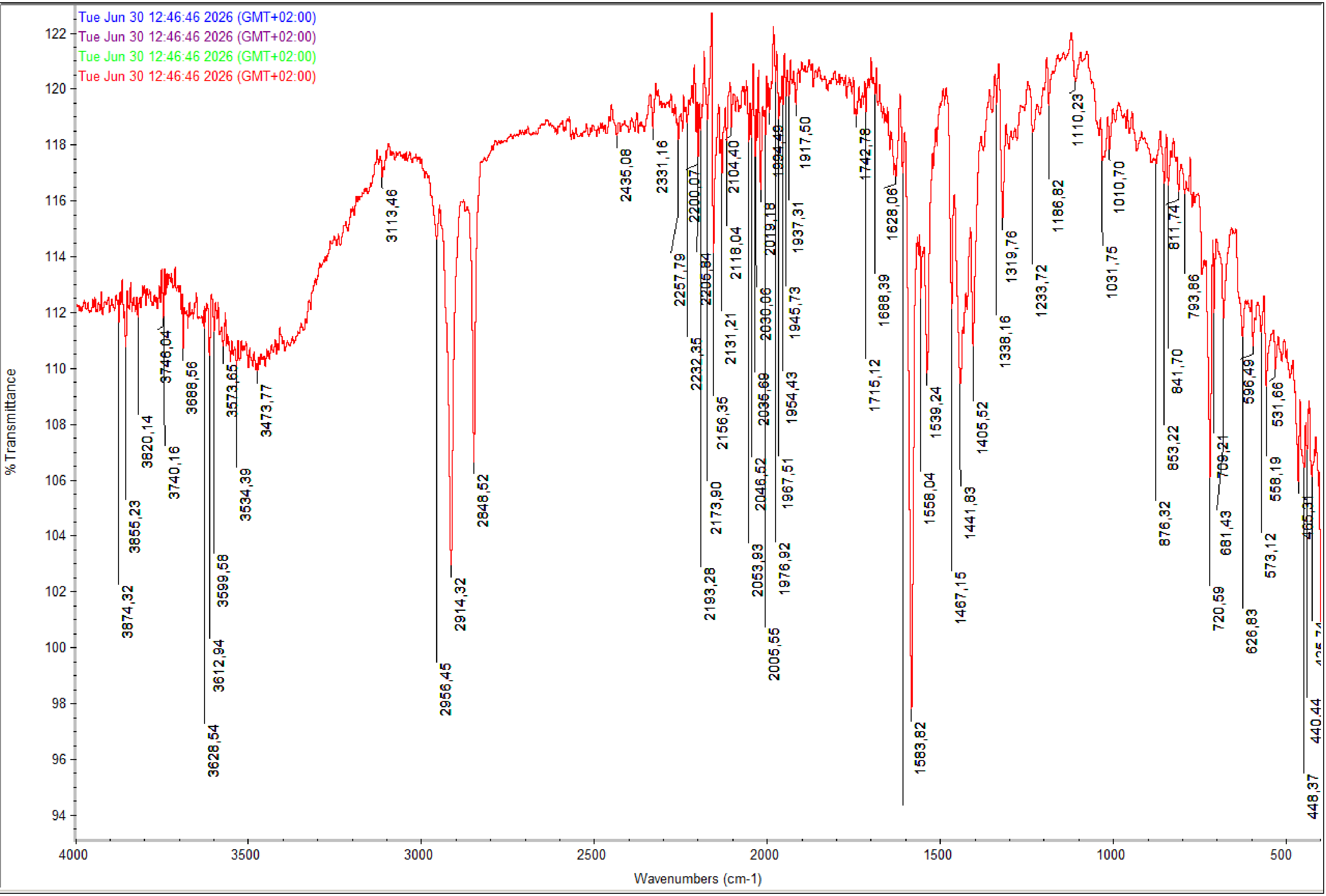

Figure S65. IR spectra Copper(II) palmitate
